# Atypical PI3Ks coordinate chemotaxis, signaling dynamics and multicellular development in *Dictyostelium*

**DOI:** 10.1242/jcs.265041

**Published:** 2026-10-02

**Authors:** Laith Bahlouli, Erik Zhang, Eric Jung, Taylor Brentjens, Elizabeth A. Rose, Harrison Drebin, Benjamin Edwards, Isabella Wischik, Alyssa B. Huynh, William Callahan, Jacqueline M. Shen, Susanna Li, Kevin Zhangxu, Marc Edwards

**Affiliations:** Department of Biology, Amherst College, Amherst, MA 01002, USA

**Keywords:** *Dictyostelium*, Cell migration, PI3K, Signaling, Chemotaxis

## Abstract

Phosphoinositide 3-kinase (PI3K) signaling regulates protrusion, polarity, membrane uptake and multicellular development in *Dictyostelium discoideum*, but these functions have been interpreted largely through canonical class I PI3Ks and phosphatidylinositol (3,4,5)-trisphosphate [PI(3,4,5)P_3_] production. This framework does not fully explain how PI3K-dependent pathways restrain Ras activity, organize relay signaling or coordinate the transition from single-cell migration to multicellular aggregation. Here, we show that three atypical PI3K-family enzymes, PikF, PikG and PikH, define genetically separable functions within this broader PI3K signaling network. PikF attenuates Ras–phosphoinositide–actin signaling, limiting protrusive activity so that chemotactic responses remain spatially and temporally constrained. PikG is required for aggregation and supports adenylyl cyclase A-dependent cAMP relay, coupling cellular polarity to the collective signaling needed for streaming and multicellular development. PikH separates uptake from these chemotactic and developmental functions by supporting efficient phagocytosis, with little effect on acute cAMP-stimulated signaling. Together, these findings expand the *Dictyostelium* PI3K framework beyond a class I PI(3,4,5)P_3_-centered pathway and identify atypical PI3Ks as specialized regulators of signal attenuation, cAMP relay organization and membrane uptake.

## Teaser

**Summary:** Atypical PI3Ks diversify *Dictyostelium* signaling by separating Ras attenuation, ACA-dependent aggregation and phagocytic uptake into distinct functional branches.

## INTRODUCTION

Cell migration underlies a wide range of physiological and pathological processes, including multicellular development, immune surveillance, wound healing and cancer metastasis (Ridley et al., 2003; Garcia and Parent, 2008; Friedl and Wolf, 2010). Directed migration depends on coordinated signaling and cytoskeletal remodeling, which together generate polarized cell states characterized by spatially confined protrusive activity at the front and contractile activity at the rear (Swaney et al., 2015; Yamada and Sixt, 2019). In amoeboid cells ranging from *Dictyostelium discoideum* to neutrophils, front–back domains can emerge even in the absence of external gradients, consistent with models in which polarity is governed by excitable signal-transduction networks shaped by coupled positive- and negative-feedback loops (Devreotes et al., 2017; Pal et al., 2019). In *Dictyostelium*, starvation-induced development uses these polarity and excitability networks to build a multicellular structure from a field of individual cells (Loomis, 2014). Periodic cyclic adenosine monophosphate (cAMP) signaling provides both a communication system between cells and a navigational cue that engages the polarity machinery within each cell (Hashimura et al., 2019). In response, cells polarize, migrate and stream toward aggregation centers, forming multicellular mounds that undergo morphogenesis to generate fruiting bodies (Dormann and Weijer, 2003; Kimmel and Parent, 2003; Weijer, 2004).

Phosphoinositide signaling is a central component of amoeboid polarity networks. In *Dictyostelium* and neutrophil chemotaxis, Ras activation, PI3K activity and phosphatidylinositol (3,4,5)-trisphosphate [PI(3,4,5)P_3_] accumulation are associated with front-state signaling, whereas PTEN, myosin II and phosphatidylinositol 3,4-bisphosphate [PI(3,4)P_2_]-enriched domains are associated with complementary rear-state organization (Charest and Firtel, 2006; Swaney et al., 2010; Lam et al., 2012; Li et al., 2021). Together, these opposing front- and rear-state modules form a partially redundant signaling system that regulates protrusion dynamics, inhibitory control and actin organization. Although PI(3,4,5)P_3_ is enriched at the front of cells during chemotaxis, work in *Dictyostelium* and neutrophils has shown that PI3K–PI(3,4,5)P_3_ signaling is not the sole determinant of directional sensing or migration (Ferguson et al., 2007; Hoeller and Kay, 2007). Instead, PI3K–PI(3,4,5)P_3_ signaling contributes to the biasing, amplification and persistence of protrusive activity, shaping how pseudopods are generated, split, selected and maintained during migration (Postma et al., 2004; Andrew and Insall, 2007). These same properties become central during development, when protrusive behavior and polarity must be coordinated for cAMP-mediated aggregation (Dormann et al., 2002; Weijer, 2004).

Despite this broad functional scope, *Dictyostelium* PI3K signaling has largely been interpreted through the lens of canonical PI(3,4,5)P_3_-generating class I enzymes. This framework is grounded in genetic studies linking *Dictyostelium* class I PI3Ks to chemoattractant-stimulated PI(3,4,5)P_3_ production, chemotactic signaling and PI3K-dependent macropinocytosis (Funamoto et al., 2002; Hoeller and Kay, 2007; Hoeller et al., 2013). Similarly, perturbing PI(3,4,5)P_3_ turnover through loss of the phosphatases PTEN and Dd5P4 disrupts the timing and spatial organization of phosphoinositide signaling across processes ranging from migration to phagocytosis and macropinocytosis (Iijima and Devreotes, 2002; Loovers et al., 2007). However, several observations remain difficult to explain from a class I PI3K-centered framework alone. These include the timing of protrusive signaling during Ras–PI3K excitability, PI3K-dependent control of adenylyl cyclase A (ACA) activation and adaptation, and the organization of PI(3,4)P_2_-enriched rear and endocytic membrane domains (Postma et al., 2004; Comer and Parent, 2006; Li et al., 2018, 2021).

The source and spatial organization of PI(3,4)P_2_ are especially difficult to reconcile with a strictly class I PI3K-centered model. Although PI(3,4)P_2_ has often been viewed as a product of PI(3,4,5)P_3_ turnover by inositol 5-phosphatases, including SHIP-family enzymes in mammalian cells and the OCRL homolog Dd5P4 in *Dictyostelium*, it has emerged as a spatially organized signaling lipid in its own right (Loovers et al., 2007; Nishio et al., 2007; Hawkins and Stephens, 2016). PI(3,4)P_2_ has been implicated in endocytic and phagocytic membrane remodeling, cytoskeletal regulation, Ras-dependent polarity and rear-state identity (Loovers et al., 2007; Posor et al., 2013; Hawkins and Stephens, 2016; Li et al., 2021). Recent work in *Dictyostelium* showed that PI(3,4)P_2_ persists at the plasma membrane after pharmacological inhibition of PI3K-driven PI(3,4,5)P_3_ production and continues to accumulate at rear membranes and macropinosomes in PI3KA and PI3KB double-null cells (Li et al., 2021). These observations suggest that a population of PI(3,4)P_2_ is generated independently of canonical class I PI3K-mediated PI(3,4,5)P_3_ production, potentially through alternative phosphoinositide-conversion routes involving phosphatidylinositol 4-phosphate (PI4P) and/or phosphatidylinositol 3-phosphate (PI3P) (Posor et al., 2013; Hawkins and Stephens, 2016; Wallroth and Haucke, 2018; Li et al., 2021).

The persistence of PI(3,4)P_2_ outside canonical class I PI(3,4,5)P_3_ production shifts attention to non-class I PI3K-family enzymes as potential contributors to *Dictyostelium* polarity and development. Class III PI3Ks generate PI3P, whereas metazoan class II PI3Ks can generate PI(3,4)P_2_ from PI4P and regulate endocytic membrane remodeling (Balla, 2013; Posor et al., 2013; Hawkins and Stephens, 2016; Wallroth and Haucke, 2018; Gulluni et al., 2019). In *Dictyostelium*, the class III branch is represented by the Vps34-like kinase PikE (also known as DdPIK5), which has been linked to protein trafficking, growth and multicellular development (Zhou et al., 1995). However, *Dictyostelium* appears to lack canonical class II PI3Ks (Engelman et al., 2006; Brown and Auger, 2011), creating an unresolved gap between PI(3,4)P_2_-dependent behaviors and the enzymes currently known to generate or remodel this lipid. This gap motivates the broader question of whether additional, atypical PI3K-family enzymes help account for PI3K-dependent control of protrusion, uptake, signaling dynamics and developmental coordination that cannot be fully explained by the canonical class I PI(3,4,5)P_3_ pathway.

Here, we identify and characterize three atypical PI3Ks – PikF, PikG and PikH – as regulators of PI3K-dependent behavior in *Dictyostelium*. Comparative sequence analysis and phylogenetic placement suggest that these enzymes diverge from canonical class I PI3Ks at activation-loop features predicted to influence phosphoinositide substrate engagement (Pirola et al., 2001). Functionally, these enzymes define distinct, non-redundant PI3K-dependent modules: PikH supports efficient phagocytic uptake, PikF limits the duration and spatial organization of protrusive signaling, and PikG is required for endogenous cAMP oscillations and aggregation-stage coordination. Together, these findings expand the landscape of PI3K signaling in *Dictyostelium* and identify atypical PI3Ks as regulators of membrane uptake, chemotaxis and developmental coordination beyond the canonical class I framework.

## RESULTS

### PikF, PikG and PikH are atypical PI3K-family enzymes distinct from canonical class I PI3Ks

To determine how PI3K signaling is diversified beyond canonical class I output in *Dictyostelium*, we analyzed PikF, PikG and PikH using comparative sequence analysis and cell biological assays. Alignment of the PI3K activation loop region showed that PikF, PikG and PikH lack the positively charged residue predicted to stabilize the 5′ phosphate of PI(4,5)P_2_, distinguishing them from canonical class I PI3Ks in both humans and *Dictyostelium* (Fig. 1A,B). Phylogenetic analysis similarly separated PikF and PikG from the class I-like PikA/PikB/PikC clade, whereas PikH clustered closer to Vps34/class III-like PI3Ks (Fig. 1C). Comparative sequence analysis suggested that PikF, PikG and PikH represent atypical PI3Ks with signaling outputs distinct from those of canonical class I enzymes.

**Fig. 1.**
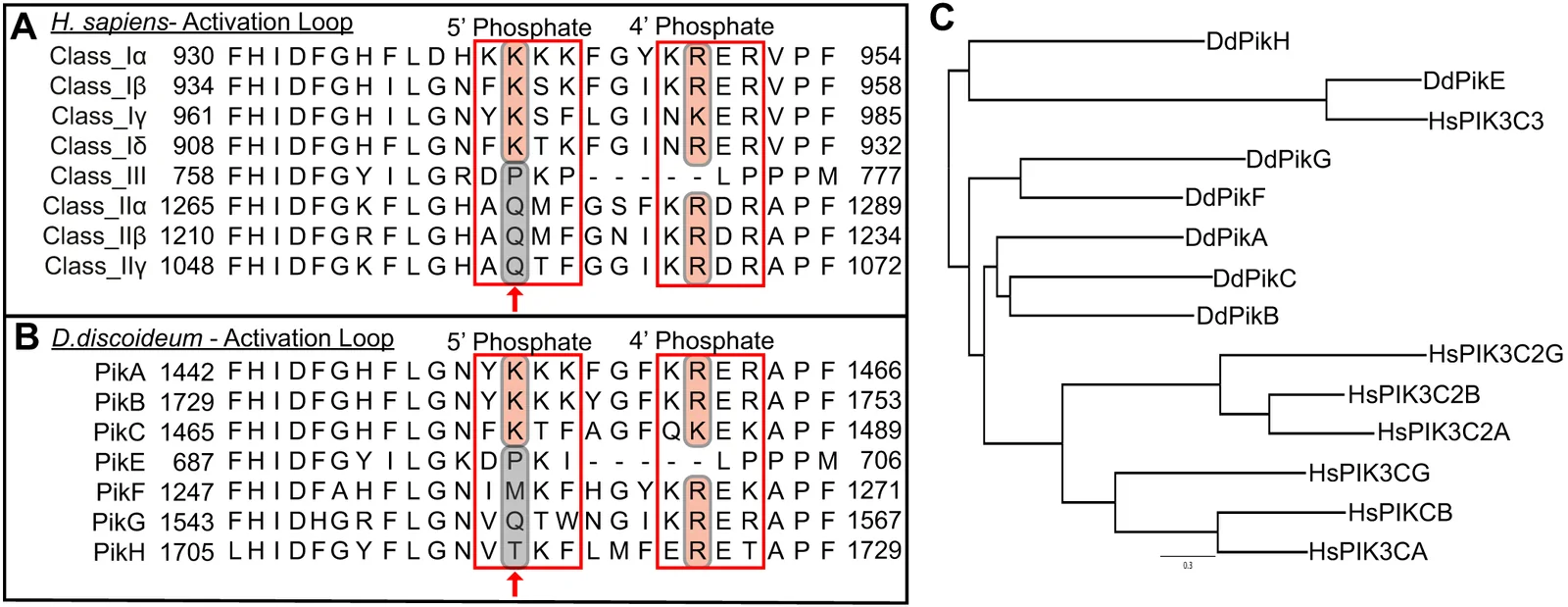
**Activation-loop sequence alignment and guide-tree comparison of human and *Dictyostelium* PI3Ks.** (A,B) Alignment of activation-loop sequences from human (A) and *Dictyostelium discoideum* (B) PI3Ks. Residues in the putative 4′ and 5′ phosphate-stabilizing basic boxes are highlighted in red. Arrows indicate the conserved lysine present in class I PI3Ks but absent from human class II and III PI3Ks, and *Dictyostelium* PikE–PikH. (C) Maximum-likelihood guide tree generated from human and *D. discoideum* PI3K activation-loop sequences. The scale bar indicates evolutionary distance. The PikF, PikG and PikH group separates from the canonical *Dictyostelium* class I PI3Ks PikA–PikC, consistent with divergence in activation-loop features that distinguish class I from non-class I PI3Ks.

### PikF and PikG regulate vegetative motility, but loss of PikF uniquely disrupts chemotactic directionality

PI3Ks regulate multiple actin-dependent processes, so we first tested whether PikF, PikG and PikH contribute to motility in vegetative cells. We performed time-lapse motility experiments by widefield imaging vegetative-stage Ax2 wild-type, *pikF^−^*, *pikG^−^* and *pikH^−^* cells (Hoeller and Kay, 2007) for 1 h, followed by single-cell tracking. *pikH^−^* cells behaved most similarly to Ax2 cells, whereas *pikF^−^* and *pikG^−^* cells exhibited less persistent trajectories (Fig. 2A–D). Quantification confirmed pronounced reductions in both speed and persistence in *pikF^−^* and *pikG^−^* cells, with median values reduced by approximately 40% for speed and 50% for persistence relative to those of Ax2 in both mutants (Fig. 2E,F). Kruskal–Wallis analysis confirmed significant reductions in speed and persistence for *pikF^−^* and *pikG^−^* cells, whereas *pikH^−^* cells did not differ significantly from Ax2 cells.

**Fig. 2.**
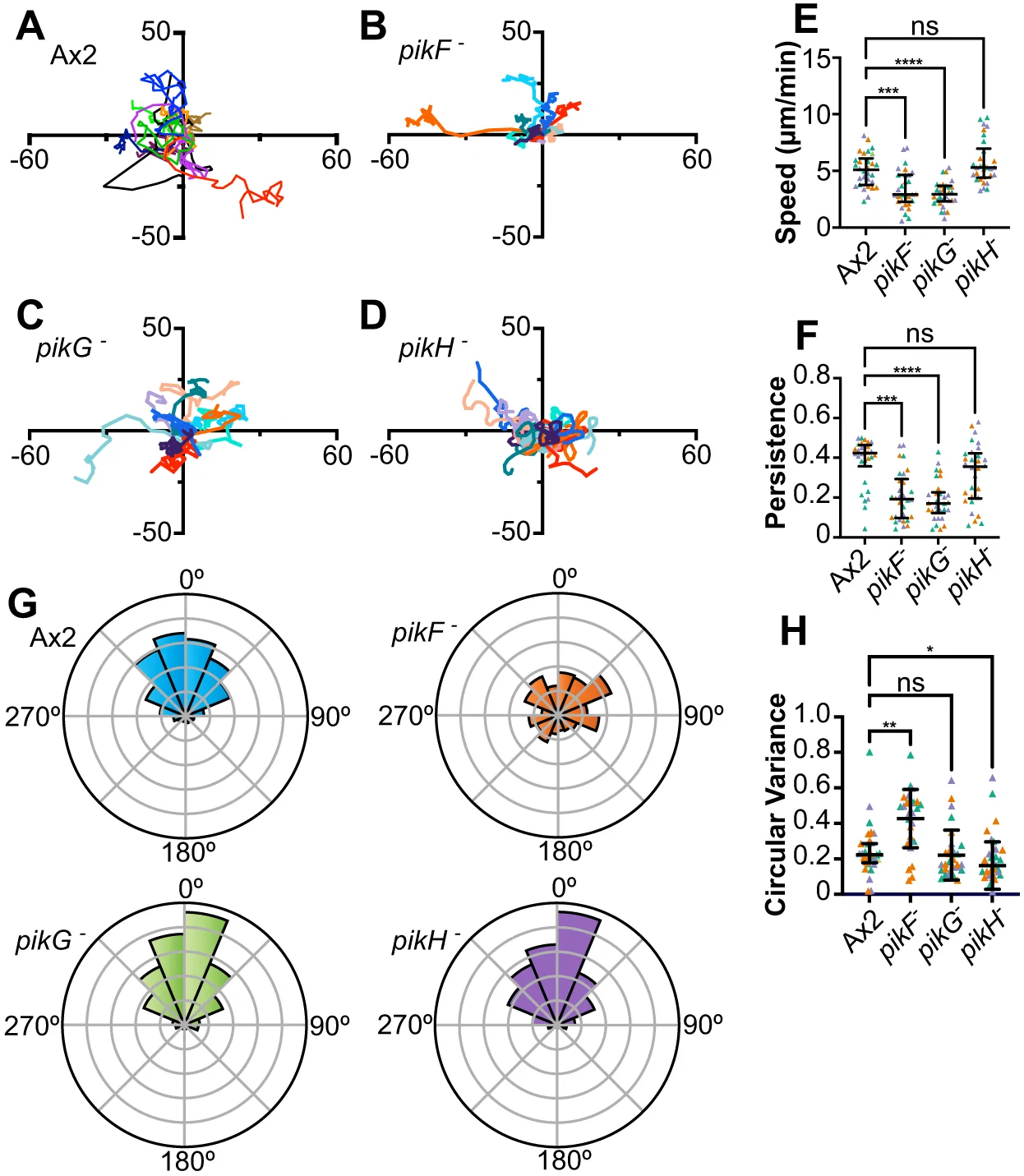
**Atypical PI3Ks differentially regulate random migration and chemotactic accuracy in *Dictyostelium*.** (A–D) Random-migration tracks of representative Ax2, *pikF*^−^, *pikG*^−^ and *pikH*^−^ cells. Ten cells per strain were tracked at 2-min intervals for 60 min and plotted from a common origin; axes are in μm. (E,F) Cell speed (E) and persistence (F) during random migration. Each point represents one cell; *n*=30 cells per strain from three biological replicates, distinguished by color. Bars indicate median and the interquartile range (IQR). (G) Rose plots showing the protrusion angle θ between each cell step and an ideal path toward the cAMP-filled microcapillary. Bars show the percentage of steps within each angular bin; each radial increment represents five percentage points. Radial-axis labels were omitted for clarity. For each strain, 100 cells across three biological replicates were analyzed, with 100 steps per cell. (H) Circular variance for the indicated strains. *n*=30 cells per strain from three biological replicates, distinguished by color. Bars indicate median and IQR. For E, F and H, significance was assessed by Kruskal–Wallis test with Dunn's multiple-comparisons test. n.s., not significant; *P<0.01; **P<0.001; ***P<0.0001; ****P<0.00001.

We then asked whether these motility defects also altered chemotaxis toward an exogenous cAMP signal. Cells were exposed to a microcapillary containing 10 μM cAMP and imaged by time-lapse microscopy. To quantify chemotactic accuracy, pseudopod angles were measured relative to a hypothetical straight-line path toward the needle, with smaller angles indicating closer alignment to the gradient. These angles were then plotted as angular distributions for each strain (Fig. 2G). Ax2 cells showed a strong bias toward the cAMP source, with pseudopods tightly clustered within 45° of the needle axis (Fig. 2G; Fig. S1A; Movie 1). By contrast, *pikF^−^* cells displayed broader pseudopod angle distributions and more meandering trajectories toward the needle, often through wide-angle lateral steps (Fig. 2G; Fig. S1B; Movie 1). This defect paralleled the reduced persistence observed during vegetative motility (Fig. 2B,F). *pikG^−^* and *pikH^−^* cells showed angular distributions more similar to those of Ax2 (Fig. 2G; Movies 2 and 3), despite the reduced speed and persistence observed for *pikG^−^* cells during random migration (Fig. 2C,E,F). Quantification of circular variance confirmed a significant increase in directional dispersion of pseudopod angles in *pikF^−^* cells but not in *pikG^−^* or *pikH^−^* cells, relative to those of Ax2 cells (Fig. 2H).

### *pikF^−^* and *pikG^−^* produce separable defects in aggregation and morphogenesis

Chemotaxis drives aggregation-stage streaming and multicellular development, raising the possibility that the migration defects in *pikF^−^* and *pikG^−^* cells alter developmental progression. We examined development on non-nutrient agar over 48 h using brightfield time-lapse imaging (Fig. 3A, Movie 4). *pikH^−^* cells developed similarly to Ax2 cells, forming mounds, slugs and fruiting bodies at approximately 8, 16 and 24 h, respectively, consistent with previous reports (Weijer, 2004) (Fig. 3A–C; Fig. S2A; Movie 4).

**Fig. 3.**
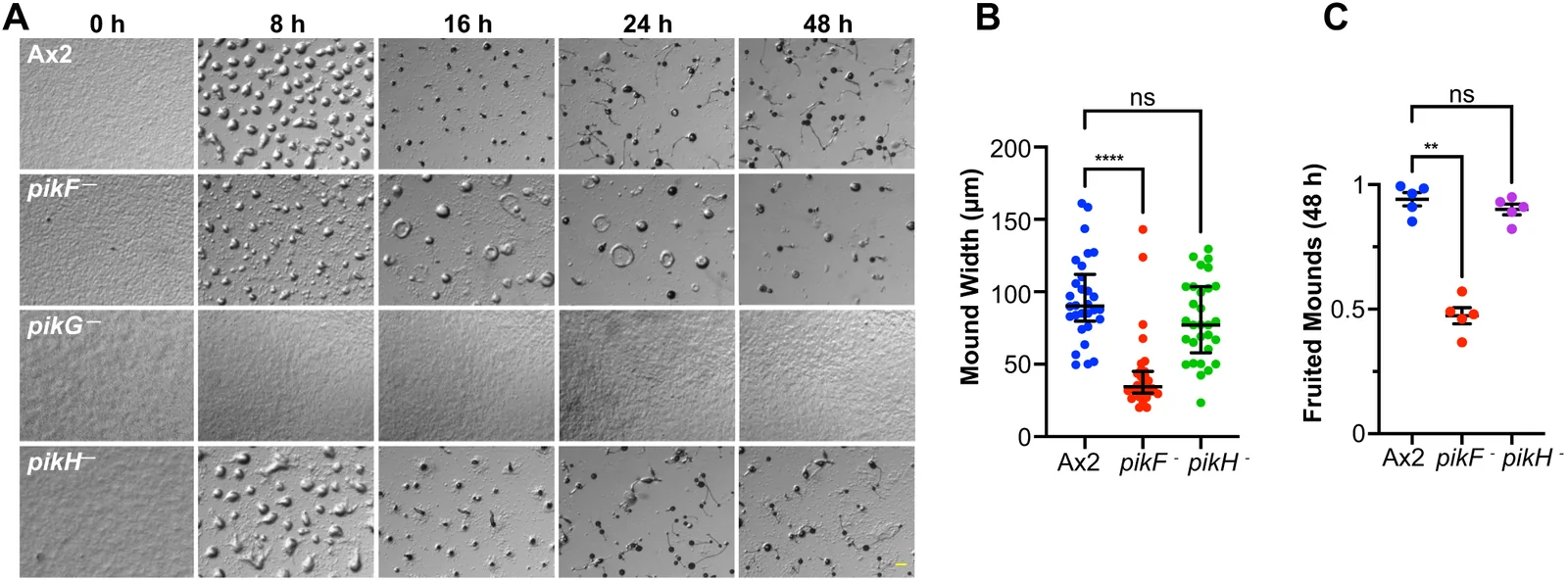
**PikF and PikG differentially regulate multicellular development in *Dictyostelium*.** (A) Representative developmental time course of Ax2, *pikF*^−^, *pikG*^−^ and *pikH*^−^ cells on non-nutrient agar. Images were acquired over 48 h at 7.3× magnification. *n*=6 independent experiments. Scale bar: 500 μm. (B) Quantification of mound width. *n*=30 mounds per strain from three biological replicates. Bars indicate median and IQR. (C) Fraction of mounds reaching the fruiting body stage by 48 h. Approximately 30 mounds were scored per plate across five biological replicates. Bars show mean±s.e.m. *pikF*^−^ cells showed delayed development with abnormal mounds and slugs, whereas pikG^−^ cells failed to complete development. *pikH*^−^ development resembled that of Ax2. Significance was assessed by Kruskal–Wallis test. n.s., not significant; **P<0.01; ****P<0.0001.

In contrast, *pikF^−^* cells showed delayed and abnormal development. Mound formation was delayed by 1–2 h relative to that for Ax2, and the mounds that formed were significantly smaller (Fig. 3A,B; Fig. S2A; Movie 4). Subsequent morphogenesis was also disrupted: *pikF^−^* cells formed small rotating structures instead of normal slugs around 20 h, and fewer than 50% of mounds reached the fruiting body stage by 48 h, whereas greater than 95% of Ax2 aggregates did so by the same time point (Fig. 3A,C; Fig. S2B; Movie 4). *pikG^−^* cells showed the most severe developmental defect, failing to initiate streaming or aggregation even after 48 h (Fig. 3A; Fig. S2A; Movie 4). These data separate the delayed, abnormal morphogenesis of *pikF^−^* cells from the more severe aggregation-stage block in *pikG^−^* cells.

### PikF and PikH support actin-dependent uptake

PI3Ks also regulate actin-dependent membrane remodeling, so we broadened our analysis to phagocytosis and micropinocytosis. *pikF^−^* and *pikH^−^* cells showed reduced uptake of both fluorescent beads and zymosan particles, whereas *pikG^−^* cells behaved similarly to Ax2 in both phagocytosis assays (Fig. 4A,B). *pikF^−^* cells also showed reduced dextran uptake, whereas *pikG^−^* and *pikH^−^* cells did not differ significantly from Ax2 (Fig. 4C). The results for *pikG^−^ and pikF^−^* cells here were consistent with previous findings (Hoeller et al., 2013). Mixed-effects analysis of the complete uptake time courses, followed by Tukey's multiple-comparisons test, confirmed these strain-specific defects for *pikF^−^ and pikH^−^* (Fig. 4D). Consistent with a kinase-dependent role for PikH, re-expression of wild-type PikH restored phagocytic uptake in *pikH^−^* cells, whereas re-expression of kinase-dead PikH^K1586A^ failed to rescue the defect (Fig. S3A,B). Mixed-effects analysis of the uptake kinetics confirmed significant rescue by wild-type PikH but not by PikH^K1586A^ (Fig. S3B). These uptake phenotypes broadened our understanding of the physiological role of PikF and PikH, while the strongest developmental phenotypes remained associated with *pikF^−^* and *pikG^−^* cells. We therefore focused next on cAMP-stimulated phosphoinositide signaling dynamics in all three mutants.

**Fig. 4.**
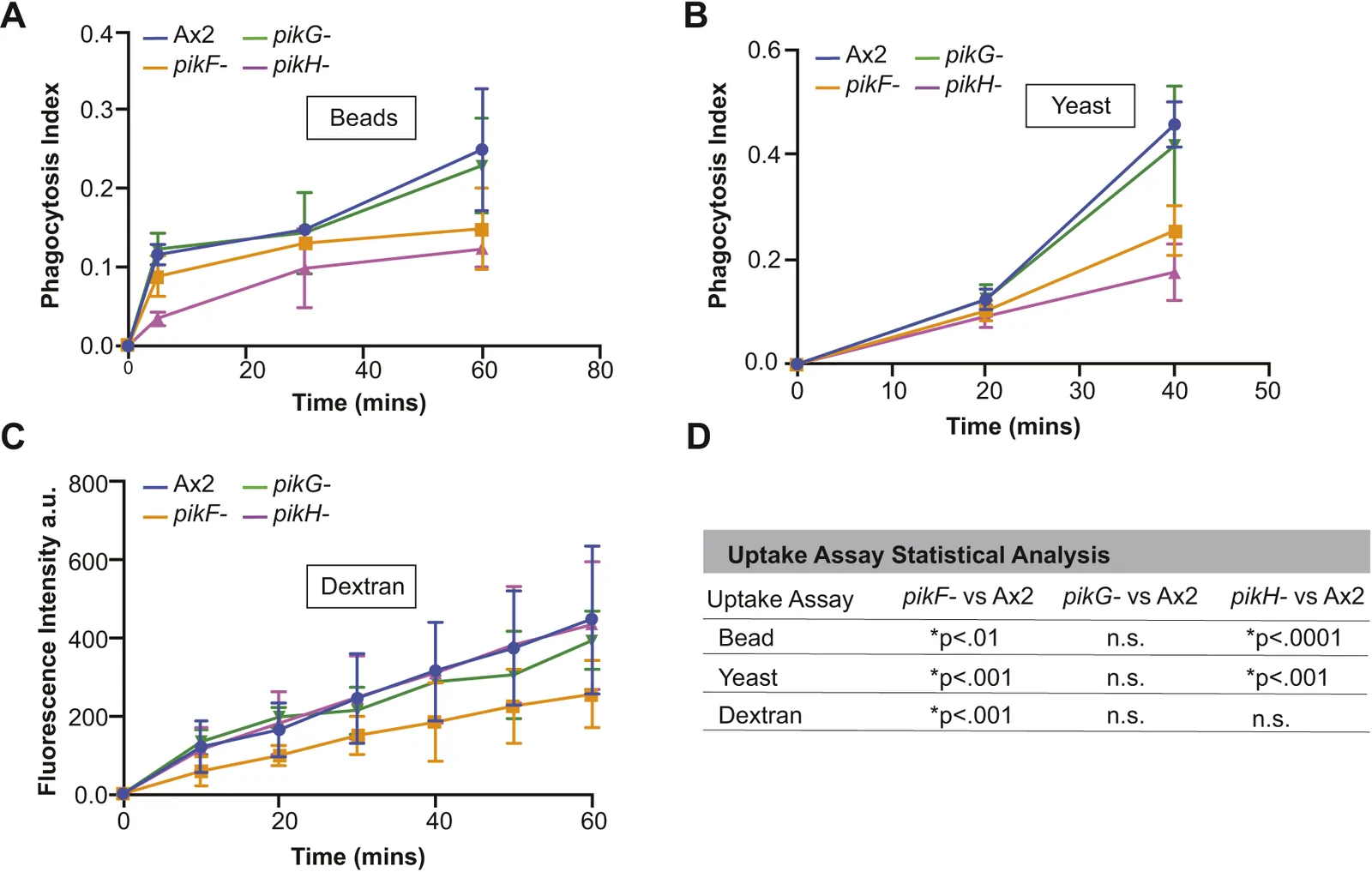
**PikF and PikH differentially regulate uptake in *Dictyostelium*.** (A) Phagocytosis index for uptake of fluorescent 4.5-μm beads by Ax2, *pikF*^−^, *pikG*^−^ and *pikH*^−^ cells, quantified as the fraction of cells containing internalized beads. *n*=60 cells per strain from three biological replicates. (B) Phagocytosis index for fluorescent zymosan uptake, quantified as in A. *n*=60 cells per strain from three biological replicates. (C) Macropinocytosis measured by fluorescent dextran uptake and quantified as the increase in intracellular fluorescence. *n*=30 cells per strain from three biological replicates. a.u., arbitrary units. (D) Statistical summary of the uptake kinetics in A–C. Time courses were analyzed using mixed-effects models with strain and time as factors, followed by Tukey's multiple-comparisons test. Comparisons of each mutant with Ax2 are shown. Exact adjusted *P*-values are reported for significant comparisons; n.s., not significant. Data in A–C are shown as mean±s.e.m.

### Loss of PikF prolongs cAMP-stimulated phosphoinositide signaling

The distinct effects of PikF, PikG and PikH on migration, development and uptake suggested that these kinases regulate actin-dependent behaviors through different signaling mechanisms. Because PI3Ks generate 3-phosphorylated phosphoinositides, we next asked whether the phenotypic differences among *pikF^−^*, *pikG^−^* and *pikH^−^* cells were accompanied by differences in PI(3,4,5)P_3_ and PI(3,4)P_2_ dynamics. Cells expressing GFP-tagged PH domain of cytosolic regulator of adenylyl cyclase [PH-CRAC–GFP, a PI(3,4,5)P_3_ biosensor] or GFP-tagged PH domain of callipygian [tPH-CynA–GFP, a PI(3,4)P2 biosensor, Swaney et al., 2015] were treated with latrunculin and stimulated uniformly with 10 μM cAMP to monitor PI(3,4,5)P_3_ and PI(3,4)P_2_ biosensor recruitment, respectively (Fig. 5). Latrunculin treatment suppressed actin-driven shape changes, allowing cAMP-dependent membrane recruitment to be quantified without changes in cell geometry confounding the analysis.

**Fig. 5.**
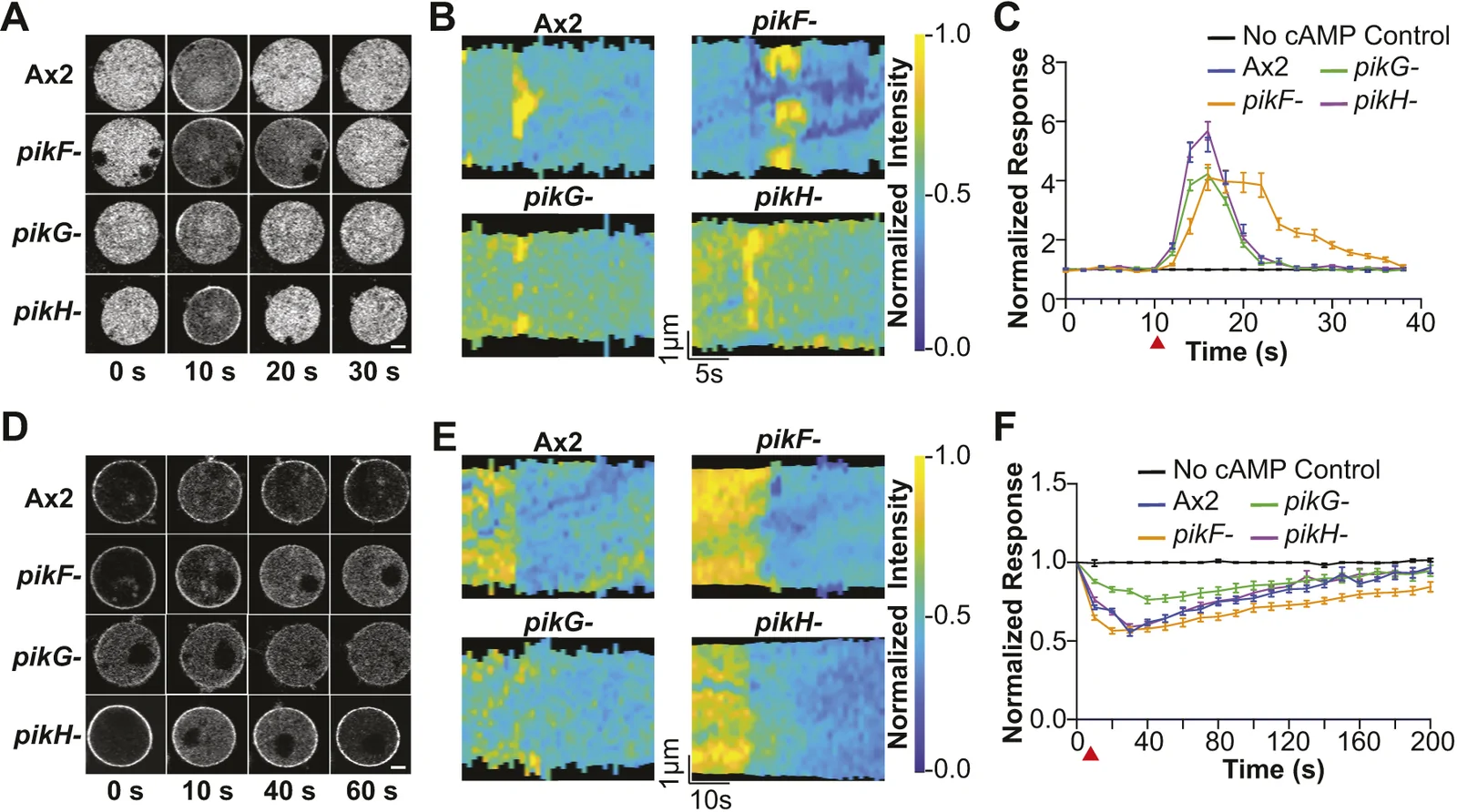
**PikF prolongs phosphoinositide signaling dynamics, whereas PikG blunts acute responses to cAMP stimulation.** (A) Representative time-course images of PH-CRAC–GFP, a PI(3,4,5)P_3_ biosensor, in latrunculin-treated Ax2, *pikF*^−^, *pikG*^−^ and *pikH*^−^ cells following uniform stimulation with 10 μM cAMP. Cells were imaged over 40 s. Scale bar: 2 μm. (B) Representative kymographs of cells shown in A. (C) Baseline-normalized membrane-to-cytosol ratio of PH-CRAC–GFP. cAMP was added at 10 s, indicated by the red arrowhead. *n*=60 cells per strain from three biological replicates. Data show mean±s.e.m. (D) Representative time-course images of tPH-CynA–GFP, a PI(3,4)P_2_ biosensor, in cells treated and stimulated as in A. Cells were imaged over 200 s. Scale bar: 2 μm. (E) Representative kymographs of cells shown in D. (F) Baseline-normalized membrane-to-cytosol ratio of tPH-CynA–GFP. cAMP was added at 10 s, indicated by the red arrowhead. *n*=60 cells per strain from three biological replicates. Data show mean±s.e.m.

In Ax2 and *pikH^−^* cells, PH-CRAC–GFP rapidly accumulated at the membrane after cAMP stimulation, appearing in time-course images and kymographs as a brief membrane translocation followed by signal decay (Fig. 5A,B). Quantification of the membrane-to-cytosol ratio confirmed similar peak responses and recovery kinetics in Ax2 and *pikH^−^* cells, with statistically indistinguishable peak amplitudes and return to the pre-stimulation baseline within approximately 20–25 s after stimulation (Fig. 5C; Fig. S4A). By contrast, *pikF^−^* cells showed prolonged PH-CRAC–GFP membrane recruitment, with a median time to return to baseline approximately 20% longer than that of Ax2 despite a similar peak response (*P*<0.01, log-rank test; Fig. 5A–C; Fig. S4A). *pikG^−^* cells displayed recovery kinetics similar to those of Ax2 but showed an approximately 20% reduction in peak response amplitude (*P*<0.05, Dunn's post hoc test), consistent with a blunted acute PI(3,4,5)P_3_ response after cAMP stimulation (Fig. 5A–C; Fig. S4A).

We observed a related pattern using tPH-CynA–GFP to monitor PI(3,4)P_2_ dynamics. In Ax2 and *pikH^−^* cells, cAMP stimulation caused a rapid decrease in membrane-associated tPH-CynA–GFP signal, visible in both the time-course images and kymographs, followed by gradual recovery toward baseline (Fig. 5D,E). Quantification of the membrane-to-cytosol ratio confirmed similar peak responses and recovery kinetics in Ax2 and *pikH^−^* cells (Fig. 5F; Fig. S4B). *pikF^−^* cells showed a comparable initial decrease in tPH-CynA–GFP signal but significantly delayed recovery to 50%, with a median recovery time approximately 25% longer than that of Ax2 (*P*<0.05, log-rank test; Fig. 5D–F; Fig. S4B). *pikG^−^* cells recovered with kinetics similar to those of Ax2 but showed an approximately 20% smaller stimulation-induced decrease in the membrane-to-cytosol ratio (*P*<0.05, Dunn's post hoc test; Fig. 5D–F; Fig. S4B). Because *pikH^−^* cells showed largely Ax2-like migration, development and cAMP-stimulated phosphoinositide responses, subsequent mechanistic experiments focused on the more pronounced *pikF^−^* and *pikG^−^* phenotypes.

### Biochemical PI(3,4,5)P_3_ measurements further support a defect in signal attenuation in *pikF^−^* cells

Mass enzyme-linked immunosorbent assay (ELISA) provided an independent biochemical measure of the PI(3,4,5)P_3_ dynamics observed with PH-CRAC–GFP. Basal levels of PI(3,4,5)P_3_, PI(3,4)P_2_ and PI3P were not significantly different across Ax2, *pikF^−^*, *pikG^−^* and *pikH^−^* cells, indicating that loss of these enzymes did not broadly alter steady-state levels of 3-phosphorylated phosphoinositides (Fig. S5A–C). We then measured cAMP-stimulated PI(3,4,5)P_3_ dynamics. In Ax2 cells, PI(3,4,5)P_3_ increased rapidly after cAMP stimulation, peaking approximately 5–8 s after stimulation and then declining back to baseline (Fig. S5D,E). *pikF^−^* cells showed a more sustained PI(3,4,5)P_3_ response, with an extended recovery phase that mirrored the prolonged PH-CRAC–GFP recruitment observed in live-cell biosensor assays (Fig. 5A–C; Fig. S4A, Fig. S5D,E). Mixed-effects analysis of the cAMP-stimulated time course identified a significant difference between Ax2 and *pikF^−^* cells over time. These biochemical measurements further support a specific defect in PI(3,4,5)P_3_ signal attenuation in *pikF^−^* cells.

### Loss of PikF increases peripheral actin activity and prolongs Ras activation

The prolonged PI(3,4,5)P_3_ response in *pikF^−^* cells, together with wide-angle protrusive behavior during chemotaxis, suggested that loss of PikF is associated with altered downstream actin dynamics. Peripheral actin activity was assessed in Ax2 and *pikF^−^* cells expressing LimE–RFP, a marker of filamentous actin assembly. Cells were imaged for 500 s, and peripheral actin patches were quantified as described in the Materials and Methods (Fig. S6A,B). *pikF^−^* cells displayed significantly more peripheral actin patches per cell than Ax2 cells, indicating elevated cortical actin activity (Fig. S6A). Representative kymographs showed the same pattern, with *pikF^−^* cells exhibiting more frequent peripheral actin patches over time than Ax2 cells (Fig. S6B).

The combination of prolonged phosphoinositide signaling and elevated peripheral actin activity pointed to Ras as a candidate contributor to the *pikF^−^* protrusive phenotype. Ras activation was monitored using GFP-tagged Ras-binding domain of human Raf1 (Raf1-RBD–GFP) as a biosensor in latrunculin-treated Ax2 and *pikF^−^* cells stimulated with uniform 10 μM cAMP (Fig. 6A,B). As with the PH-CRAC–GFP and tPH-CynA–GFP biosensor assays, Raf1-RBD–GFP recruitment was quantified as a baseline-normalized membrane-to-cytosol ratio (Fig. 6B). In Ax2 cells, Raf1-RBD–GFP rapidly accumulated at the membrane, peaked approximately 6 s after stimulation, and returned to baseline by approximately 20 s (Fig. 6A,B). In *pikF^−^* cells, Raf1-RBD–GFP showed a similar initial response but remained elevated at 24 s after stimulation, indicating prolonged Ras activation (Fig. 6A,B).

**Fig. 6.**
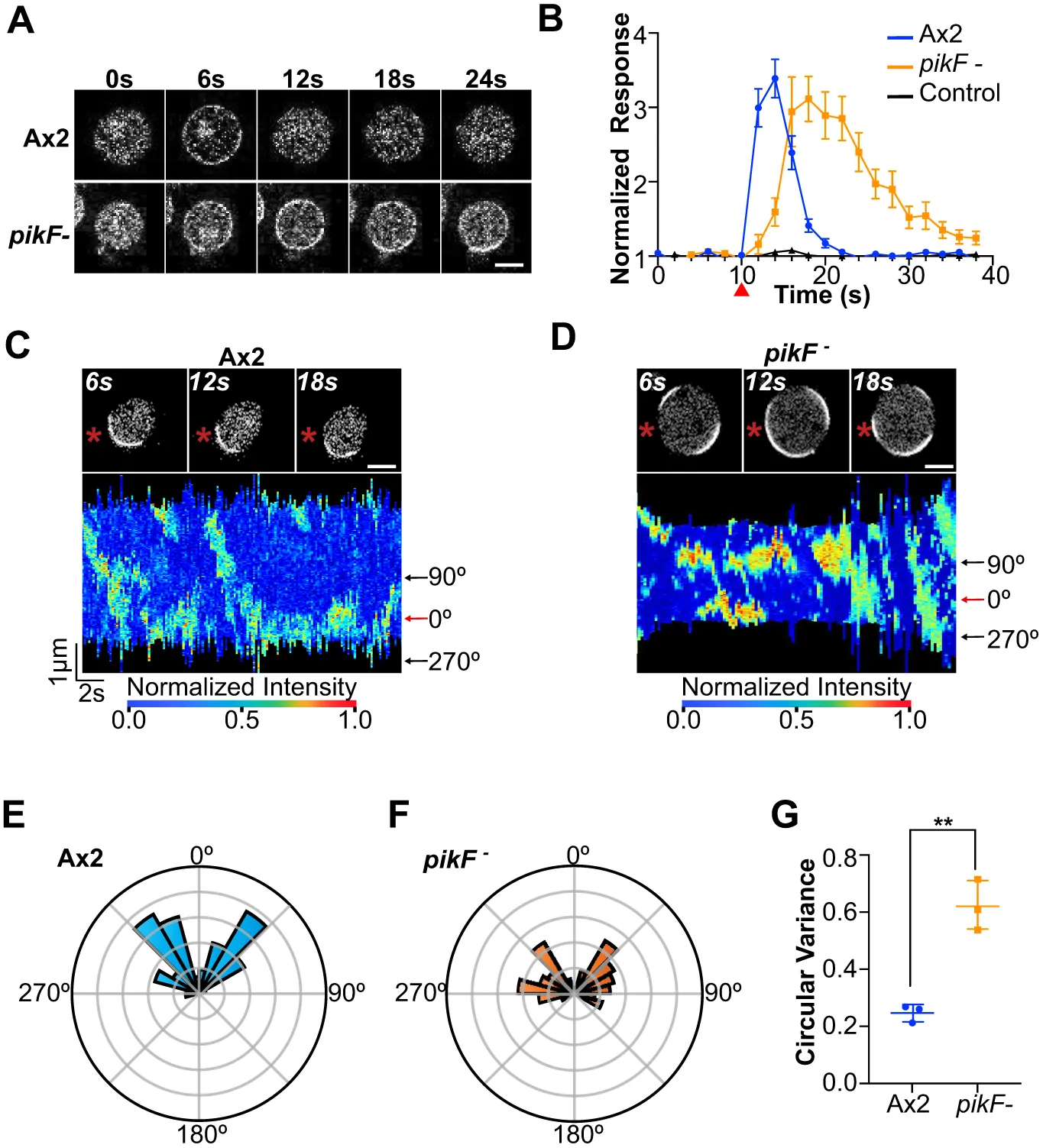
**PikF prolongs Ras activation and impairs Ras crescent positioning.** (A) Representative time-course images of Raf1-RBD–GFP in latrunculin-treated Ax2 and *pikF*^−^ cells following uniform stimulation with 10 μM cAMP. Cells were imaged every 2 s for 40 s; cAMP was added at 10 s (indicated by the red arrowhead in B). Scale bar: 10 μm. (B) Baseline-normalized membrane-to-cytosol ratio of Raf1-RBD–GFP. *n*=60 cells per strain from three biological replicates. Data show mean±s.e.m. (C,D) Representative Ras crescent assays in latrunculin-treated Ax2 (C) and *pikF*^−^ (D) cells expressing Raf1-RBD–GFP and stimulated with a microcapillary containing 10 μM cAMP, indicated by the asterisks. Corresponding kymographs are shown below. Red arrows indicate the approximate microcapillary position. Scale bars: 5 μm. (E,F) Rose plots showing the angle θ between the Ras crescent axis and the cAMP-filled microcapillary. Bars show the percentage of measurements within each angular bin; each radial increment represents five percentage points. Radial-axis labels were omitted for clarity. *n*=20 cells per strain across three biological replicates. (G) Circular variance of Ras crescent positioning calculated from E and F. Data show mean±s.d. **P<0.01 (Mann–Whitney test).

### PikF is required for spatial precision of Ras activation in cAMP gradients

We then examined whether prolonged Ras activation was accompanied by altered spatial organization of Ras activity in a cAMP gradient. Latrunculin- and caffeine-treated Raf1-RBD–GFP-expressing cells were stimulated with a 10 μM cAMP-filled microcapillary, and the angle between the central axis of the Ras crescent and the position of the needle was measured (Fig. 6C–G, Movies 5 and 6). Caffeine treatment was used to reduce basal signaling and suppress spontaneous crescent formation. In Ax2 cells, Ras crescents formed toward the cAMP source, with crescent angles clustered around the needle axis (Fig. 6C,E, Movie 5). In *pikF^−^* cells, Ras crescents were less consistently aligned with the needle and often appeared as multiple crescent-like domains around the cortex (Fig. 6D,F, Movie 6). Circular variance analysis confirmed significantly reduced precision of Ras crescent positioning in *pikF^−^* cells compared with that in Ax2 cells (Fig. 6G). As a negative control, Ax2 cells exposed to a 0 μM cAMP-filled microcapillary did not form oriented Ras crescents under otherwise identical assay conditions (Fig. S7).

### PikG is required for endogenous cAMP oscillations and ACA localization

The more severe developmental phenotype of *pikG^−^* cells suggested a distinct failure in signal relay during the aggregation stage. Unlike *pikF^−^* cells, which initiated aggregation but progressed abnormally, *pikG^−^* cells failed to form streams or mounds during development (Fig. 3A; Movie 4). This defect was also evident in buffer-control needle assays, in which Ax2 cells streamed and aggregated spontaneously. In contrast, *pikG^−^* cells failed to initiate coordinated streaming, aggregation or mound formation in the absence of an external cAMP cue (Fig. S8; Movies 7 and 8). We therefore examined whether *pikG^−^* cells were defective in the endogenous oscillatory signaling that drives aggregation-stage streaming.

To follow intracellular cAMP dynamics during pre-aggregation development, we used Flamindo2–mScarlet, a fluorescent cAMP biosensor that decreases in intensity as intracellular cAMP increases. Ax2, *pikG^−^* and *aca^−^* cells expressing Flamindo2–mScarlet were developed on non-nutrient agar and imaged during the pre-aggregation period (Fig. 7A–C; Movies 9–11). Ax2 cells formed oscillatory centers with periodic changes in Flamindo2 intensity during the hour before the onset of streaming. Autocorrelation analysis identified regular oscillatory behavior, and period measurements from the mean intensity traces showed an approximately 6 min oscillation period (Fig. 7B,C; Fig. S9A,E; Movie 9). In contrast, *aca^−^* and *pikG^−^* cells did not show regular periodic changes in Flamindo2 intensity between 3 and 4 h after starvation (Fig. 7B,C; Fig. S9B,C; Movies 10 and 11). Autocorrelation analysis did not identify significant oscillatory behavior in either strain, and sine-curve fitting yielded poorer fits for aca^−^ and *pikG^−^* traces than for Ax2 traces (Fig. S9A–D). Thus, *pikG^−^* cells resemble *aca*^−^ cells in failing to establish detectable pre-aggregation cAMP oscillations.

**Fig. 7.**
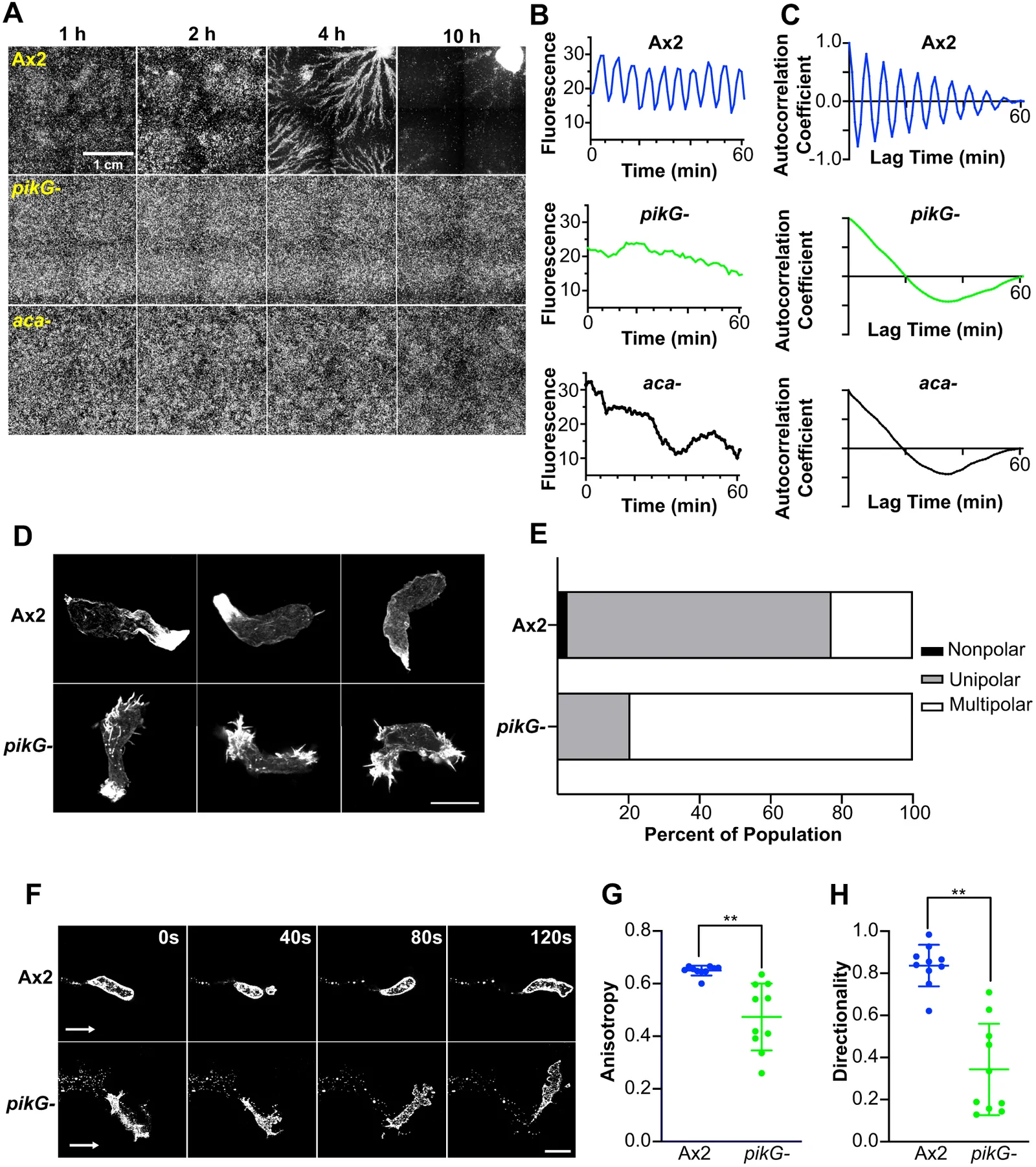
**PikG is required for pre-aggregation oscillatory signaling and ACA polarity.** (A) Representative images from developmental time courses of Ax2, *pikG*^−^ and *aca*^−^ cells expressing Flamindo2–mScarlet on non-nutrient agar. Scale bar: 1 cm. (B) Representative fluorescence intensity traces from pre-aggregation oscillatory centers. (C) Autocorrelation analysis of traces shown in B. (D) Representative fixed developed Ax2 and *pikG*^−^ cells expressing ACA–YFP. Scale bar: 5 μm. (E) ACA–YFP polarity in developed Ax2 and *pikG*^−^ cells. Cells were scored as nonpolar, unipolar or multipolar as described in the Materials and Methods. *n*=39 cells per strain. (F) Representative time-lapse images of developed Ax2 and *pikG*^−^ cells expressing ACA–YFP. Arrows indicate direction of movement. Scale bar: 10 μm. (G,H) ACA–YFP vesicle-trail anisotropy (G) and directionality (H). *n*=10 cells per strain. Data show mean±s.d. **P<0.001 (Kruskal–Wallis test).

ACA produces cAMP and becomes enriched at the rear of aggregation-stage cells (Kriebel et al., 2003), prompting us to examine ACA–YFP localization in developed Ax2 and *pikG*^−^ cells. In Ax2 cells, ACA localized asymmetrically, with many cells displaying unipolar enrichment (Fig. 7D,E). In *pikG^−^* cells, however, ACA–YFP was frequently enriched at multiple cortical sites rather than being concentrated at a single pole, consistent with the multipolar morphology of these cells and indicating disrupted ACA polarity (Fig. 7D,E).

To determine whether the multipolar ACA–YFP pattern reflected a broader defect in cellular polarity rather than a selective change in ACA localization, we examined developed Ax2 and *pikG^−^* cells expressing tPH-CynA–GFP as they migrated toward cAMP in an under-agar two-drop chemotaxis assay. Ax2 cells generally established and maintained a single front oriented toward the cAMP source. By contrast, *pikG^−^* cells frequently formed multiple spatially separated fronts during migration (Fig. S10A). Across three independent experiments, 80.0±12.0% (mean±s.d.) of Ax2 cells were unipolar compared with 18.9±8.4% of *pikG^−^* cells (adjusted *P*=0.0351; Fig. S10B). Conversely, 72.2±6.9% of *pikG^−^* cells were multipolar compared with 17.8±10.2% of Ax2 cells (adjusted *P*=0.0346). These results indicate that *pikG^−^* cells have a broader defect in establishing or maintaining a single polarity axis.

We next asked whether this altered ACA organization was accompanied by defects in the spatial pattern of ACA-containing vesicle secretion. Super-resolution time-lapse imaging at the coverslip surface revealed that both Ax2 and *pikG^−^* cells deposited ACA–YFP-positive vesicles, indicating that loss of PikG did not eliminate production or release of ACA-containing vesicle trails (Fig. 7F). However, the organization of these trails differed markedly between strains. Ax2 cells left relatively narrow, directionally organized ACA–YFP-positive trails, whereas *pikG^−^* cells deposited broader, less coherent trails with more frequent changes in direction (Fig. 7F; Movies 12 and 13). Quantification of trail anisotropy and directionality confirmed a significant (*P*<0.001) defect: *pikG^−^* trails had lower anisotropy and directionality scores than Ax2 trails, consistent with poorly coordinated spatial deposition of ACA–YFP-positive vesicles (Fig. 7G,H). Consistent with these measurements, representative time-lapse movies showed Ax2 cells moving in close succession along ACA–YFP-positive trails, including examples in which a following cell contacted the rear of a leader cell during streaming-like movement, whereas *pikG*^−^ cells interacted with ACA–YFP-positive trails without forming comparably coordinated streams (Movies 12 and 13). Thus, PikG is not strictly required for ACA–YFP-positive vesicle deposition, but it is required for the spatial organization of ACA-containing relay structures during aggregation-stage signaling.

### PikF and PikG developmental functions require conserved catalytic lysines

To determine whether the developmental defects in *pikF^−^* and *pikG^−^* cells require the predicted kinase activities of PikF and PikG, we analyzed rescue strains expressing either wild-type PikF or PikG, or kinase-dead alleles carrying mutations in the predicted catalytic lysine residues: PikF^K1119A^ and PikG^K1415A^. Development was assayed on non-nutrient agar over 48 h using the same endpoints measured in Fig. 3, including time to mound emergence and the timing of fruiting body formation (Fig. 8A–C).

**Fig. 8.**
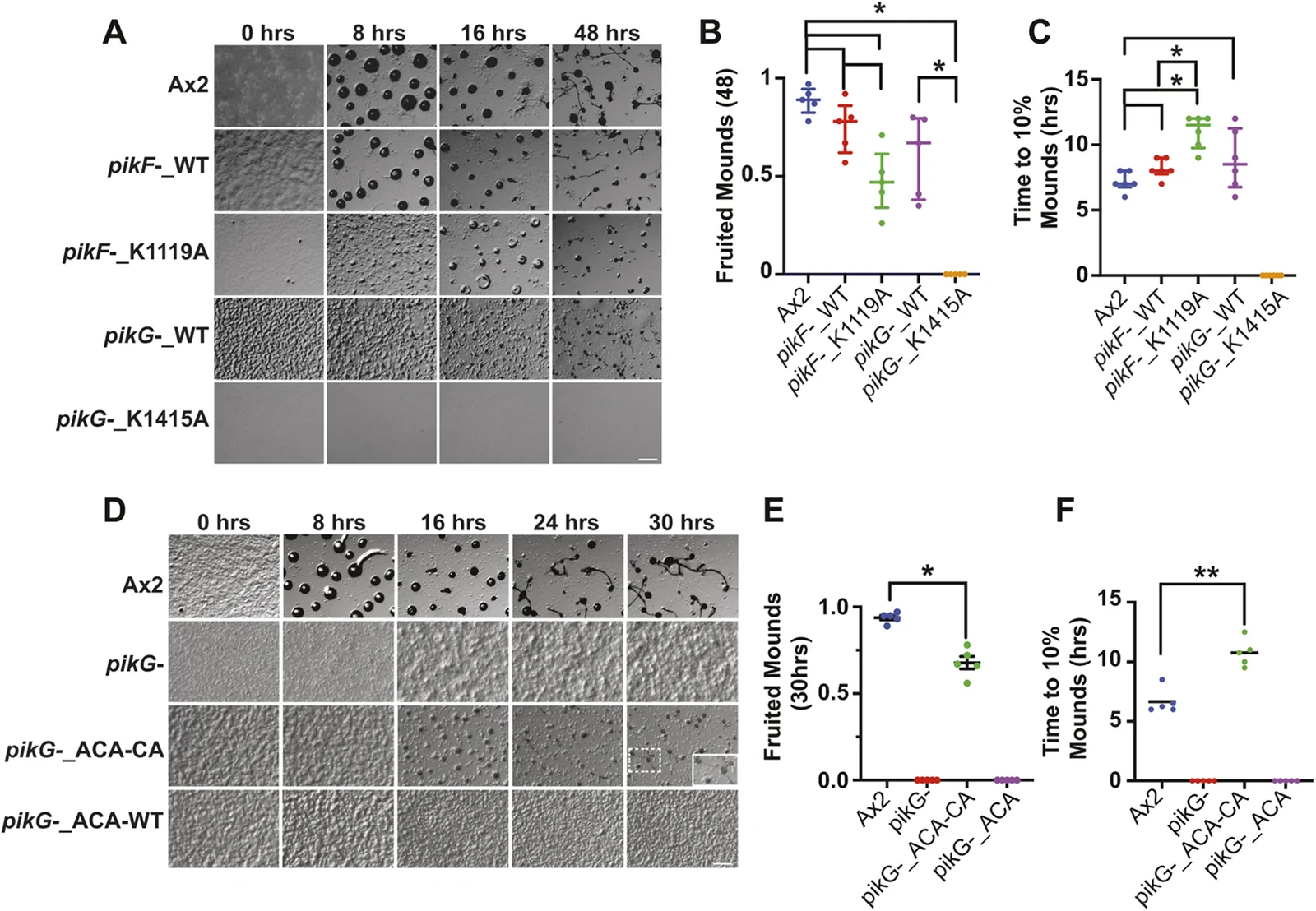
**PikF and PikG kinase activity is required for development, whereas constitutively active ACA bypasses the *pikG*^−^ developmental defect.** (A) Representative developmental time courses of the indicated strains. *pikF*^−^ and *pikG*^−^ cells expressed wild-type PikF or PikG, respectively, or the kinase-dead alleles PikF^K1119A^ and PikG^K1415A^. Images were acquired over 48 h at 7.3× magnification. *n*=5 independent experiments. Scale bar: 500 μm. (B) Fraction of mounds reaching the fruiting body stage by 48 h. Approximately 30 mounds were scored per plate. Data show mean±s.e.m. *n*=5 biological replicates. (C) Time to emergence of 10% of total mounds. Data show mean±s.e.m. *n*=5 biological replicates. (D) Representative 30-h developmental time courses of Ax2 cells, *pikG*^−^ cells, *pikG*^−^ cells expressing ACA-CA–YFP and *pikG*^−^ cells expressing wild-type ACA–YFP. The dashed rectangle indicates the region shown at higher magnification in the adjacent solid-bordered inset. Images were acquired at 7.3× magnification. *n*=5 independent experiments. Scale bar: 500 μm. (E) Fraction of mounds reaching the fruiting body stage by 30 h. Approximately 30 mounds were scored per plate. Data show mean±s.e.m. *n*=5 biological replicates. (F) Time to emergence of 10% of total mounds. Data show mean±s.e.m. *n*=5 biological replicates. Data in B, C, E and F were analyzed using mixed-effects models with condition as a fixed effect and biological replicate as a random effect, followed by Tukey's multiple-comparisons test. *P<0.05; **P<0.005.

Re-expression of wild-type PikF restored development in *pikF^−^* cells, producing mounds and fruiting bodies similar to those of Ax2 by 48 h, whereas *pikF^−^* cells expressing PikF^K1119A^ remained delayed and abnormal, resembling the knockout phenotype (Fig. 8A–C). Similarly, wild-type PikG restored aggregation and fruiting body formation in *pikG^−^* cells, whereas *pikG^−^* cells expressing PikG^K1415A^ remained aggregation defective (Fig. 8A–C). Quantification of mound emergence timing and the fraction of mounds reaching the fruiting body stage confirmed that wild-type PikF and PikG restored developmental progression, whereas the corresponding kinase-dead alleles did not (Fig. 8B,C). Thus, *PikF-* and *PikG-*dependent development requires the conserved catalytic lysines necessary for PI3K activity.

### Constitutive ACA activity partially bypasses the *pikG*^−^ developmental defect

We next tested whether increasing ACA activity could bypass the *pikG*^−^ aggregation defect. Ax2 cells, *pikG^−^* cells, *pikG^−^* cells expressing constitutively active ACA^L394S^–YFP (ACA-CA–YFP),and *pikG^−^* cells expressing wild-type ACA–YFP were developed on non-nutrient agar. Whereas *pikG^−^* cells and *pikG^−^* cells expressing ACA–YFP failed to form mounds or fruiting bodies, expression of ACA-CA–YFP restored aggregation and permitted progression to fruiting body formation (Fig. 8D–F; Movie 14). ACA-CA–YFP also accelerated mound emergence relative to *pikG^−^* cells and *pikG^−^* cells expressing wild-type ACA–YFP (Fig. 8F).

By 24 h, a significantly smaller fraction of mounds formed by *pikG^−^* cells expressing ACA-CA–YFP had reached the fruiting body stage than that in Ax2 (*P*<0.01; Fig. S11A), and the resulting fruiting bodies were significantly shorter than those formed by Ax2 cells (*P*<0.0001; Fig. S11B). Thus, constitutive ACA activity bypasses the early aggregation block caused by loss of PikG but does not fully restore the timing or morphology of later development.

Because ACA-CA could bypass the aggregation defect, we next asked whether *pikG^−^* cells retained the developmental differentiation and relay machinery required to respond to increased ACA activity. Ax2 and *pikG^−^* cells were collected before development and at 2, 4, 6 and 8 h after development was initiated, the abundance of ACA, the cAMP receptor cAR1 and the cell adhesion protein csA was examined by immunoblotting (Fig. S12A,E). Densitometric analysis showed that *pikG^−^* cells retained developmental expression of ACA, cAR1 and csA throughout the 8 h time course (Fig. S12B–D). Together with the results of the partial rescue by ACA-CA, these findings place PikG more specifically in the productive activation of ACA and the spatial organization of cAMP relay.

## DISCUSSION

### Atypical PI3Ks expand the PI3K signaling toolkit in *Dictyostelium*

*Dictyostelium* PI3K signaling has been studied largely through canonical class I-like enzymes and their PI(3,4,5)P_3_ output, yet several PI3K-dependent behaviors are not fully accounted for within that framework. This study identifies distinct cellular roles for three atypical PI3K-family enzymes. PikF constrains Ras–phosphoinositide–actin signaling during chemotaxis, PikG supports the cAMP relay that initiates aggregation, and PikH contributes to phagocytic uptake. Together, these findings indicate that PI3K-family function is distributed across related enzymes that regulate separable outputs in protrusion, uptake, relay and multicellular development.

Two unresolved areas in the field motivated this expanded view of *Dictyostelium* PI3K signaling. The strongest evidence that PI3K-dependent behavior extends beyond canonical class I PI(3,4,5)P_3_ production comes from studies investigating PI(3,4)P_2_. PI(3,4)P_2_-positive membrane domains persist after inhibitor-mediated depletion of PI(3,4,5)P_3_ and remain detectable at rear membranes and macropinosome-associated compartments in PI3K1 and PI3K2 double-null cells, suggesting that PI(3,4)P_2_ organization cannot be explained solely as a PikA- or PikB-dependent PI(3,4,5)P_3_ turnover product (Li et al., 2021). A second, more indirect tension comes from studies of cAMP relay. Broad-spectrum pharmacological PI3K inhibition by LY294002 strongly impairs ACA activation and adaptation, whereas genetic loss of the major chemoattractant-responsive class I PI3Ks PikA and PikB permits an initial ACA activation peak but disrupts adaptation (Comer and Parent, 2006). This mismatch suggests that ACA regulation involves PI3K-dependent inputs not captured by PikA/PikB loss alone. This interpretation remains provisional because LY294002 has off-target effects, including effects on TOR signaling, and because *pi3kA^−^* and *pi3kB^−^* double-null cells retain additional PI3K-family enzymes. In this context, the failure of *pikG^−^* cells to generate endogenous cAMP oscillations or initiate productive aggregation identifies PikG as a genetic entry point into a PI3K-dependent relay function not readily explained by canonical class I PI(3,4,5)P_3_ production alone.

### PikF attenuates Ras–phosphoinositide signaling and constrains protrusive activity

PikF plays a critical role in signal attenuation downstream of chemotactic stimulation. Unlike canonical class I PI3Ks, which promote early cAMP-induced phosphoinositide signaling, PikF limits the duration and spatial spread of Ras–phosphoinositide–actin signaling after stimulation. This interpretation is supported by prolonged Ras activation, extended PH-CRAC–GFP recruitment, sustained PI(3,4,5)P_3_ measured by PIP mass ELISA, elevated peripheral actin activity, and reduced directional precision during chemotaxis in *pikF^−^* cells (Fig. 2G,H, Fig. 5A–C, Fig. 6; Fig. S5D,E, Fig. S6). Together, these phenotypes place PikF at the level of signal attenuation rather than signal initiation.

A compelling model is that PikF helps couple PI(3,4)P_2_-associated membrane dynamics to a refractory state that restrains Ras activity. This model fits the mutually inhibitory Ras–PI(3,4)P_2_ polarity framework, in which Ras activity marks front-state signaling, whereas PI(3,4)P_2_ is associated with rear-state identity, protrusion suppression and recruitment of PI(3,4)P_2_-responsive Ras GTPase-activating proteins that locally restrain Ras activity (Li et al., 2018). The ‘reverse-fountain’ model further suggests that PI(3,4)P_2_-enriched macropinosomes give rise to vesicles that deliver PI(3,4)P_2_ to the rear membrane, helping to maintain a back-to-front polarity system (Li et al., 2021). In this context, the *pikF^−^* phenotype is consistent with defective coupling of PI(3,4)P_2_-associated membrane dynamics to Ras attenuation: Ras and PI(3,4,5)P_3_ signaling persist, actin activity increases, and cortical signaling becomes less temporally and spatially constrained.

PikF is an attractive candidate for this role because *pikF^−^* cells show delayed PI(3,4)P_2_ biosensor recovery together with prolonged Ras and PI(3,4,5)P_3_ signaling after cAMP stimulation (Fig. 5, Fig. 6A,B; Fig. S4, Fig. S5D,E). The activation-loop features and sequence relationships described in Fig. 1 are also compatible with divergence from canonical class I PI3Ks and with altered phosphoinositide substrate engagement. These data do not establish that PikF directly generates PI(3,4)P_2_ or defines the relevant PI(3,4)P_2_-associated refractory pool. An alternative possibility is that PikF indirectly restricts the activity or persistence of the canonical cAMP-responsive PI3Ks PikA and PikB through another signaling or trafficking mechanism. The present data distinguish neither model, but they support the more general conclusion that PikF promotes attenuation of Ras–phosphoinositide signaling after chemotactic stimulation.

The physiological consequences of PikF loss are evident across migration, uptake and development (Figs 2–4). During random migration, failure to constrain Ras-driven actin activity would be expected to increase lateral protrusions and reduce persistence. During chemotaxis, the same failure would permit protrusive responses away from the cAMP source, producing the wide-angle steps and meandering paths observed in *pikF^−^* cells. This represents a defect in chemotactic precision rather than an inability to detect or migrate toward cAMP. The distinction might also help reconcile the phenotype of the individual *pikF^−^* mutant with the retained chemotaxis reported for cells lacking PikA, PikB, PikC, PikF and PikG together (Hoeller and Kay, 2007). Simultaneous loss of PI3Ks that promote canonical PI(3,4,5)P_3_ signaling and one that contributes to signal attenuation could produce a different net phenotype from loss of PikF alone due to combined pleiotropic effects in the quintuple PI3K knockout (Hoeller and Kay, 2007).

The reduced macropinocytosis observed in *pikF^−^* cells is consistent with earlier work examining uptake-associated functions of these PI3K-family members and confirms that PikF contributes to actin-dependent membrane remodeling beyond chemotaxis (Hoeller and Kay, 2007; Hoeller et al., 2013). Macropinocytosis and large-particle uptake require spatially organized Ras–PI3K–PI(3,4,5)P_3_ signaling and coordinated cup closure, and PI(3,4)P_2_ has been implicated during later cup stages (King and Kay, 2019). Impaired signal attenuation or closure could therefore reduce uptake efficiency without abolishing cup formation. Similar defects in the control of protrusion and membrane remodeling provide a plausible explanation for delayed mound formation and smaller aggregates, because streaming requires repeated, directionally refined responses to relay cues. The later slug and fruiting body abnormalities might reflect cumulative defects in aggregation and tissue organization or additional functions of PikF during morphogenesis.

### PikG supports ACA-dependent cAMP relay during aggregation

Whereas PikF regulates how cells attenuate and spatially constrain chemotactic signaling, PikG supports the paracrine relay system required for streaming and aggregation. *pikG^−^* cells fail to stream or aggregate and do not generate detectable Flamindo2 oscillations during pre-aggregation development (Fig. 3A, Fig. 7A–C). Because Flamindo2 reports intracellular cAMP dynamics, this failure indicates that *pikG^−^* cells do not enter the periodic cAMP signaling state normally associated with relay-competent aggregation. In wild-type cells, cAMP stimulation activates a Ras–PI3K module that generates PI(3,4,5)P_3_ and recruits CRAC, promoting ACA activation, intracellular cAMP production, secretion and relay to neighboring cells (Dormann et al., 2002; Comer and Parent, 2006). The absence of detectable oscillations in *pikG^−^* cells, despite their ability to migrate toward an imposed cAMP source, therefore points to a failure of the endogenous relay circuit rather than a general inability to sense cAMP.

The ACA-CA rescue places PikG within the pathway controlling productive ACA-dependent relay. Constitutively active ACA bypassed the early aggregation block in *pikG^−^* cells and permitted mound and fruiting body formation, whereas expression of wild-type ACA did not restore development (Fig. 8D–F; Fig. S11; Movie 14). This rescue is unlikely to reflect correction of a broad failure in developmental differentiation because *pikG^−^* cells retained developmental expression of ACA, cAR1 and CsaA (Fig. S12). Together, these findings place the *pikG^−^* defect upstream of or at the level of productive ACA activation without establishing that PikG directly regulates ACA.

The rescue was incomplete; *pikG^−^* cells expressing ACA-CA progressed more slowly than Ax2 cells and produced shorter fruiting bodies (Fig. S11). PikG therefore acts close enough to ACA activation that constitutive cyclase activity can overcome the initial relay defect, while also contributing to spatial or developmental functions that ACA-CA cannot fully replace. One such function might be the establishment of a stable polarity axis. ACA–YFP localization was frequently multipolar in *pikG^−^* cells, and an independent under-agar assay showed that migrating *pikG^−^* cells often formed multiple spatially separated fronts while moving toward cAMP (Fig. 7D,E; Fig. S10). The multipolar ACA pattern therefore occurs within a broader defect in establishing or maintaining cellular polarity rather than representing an isolated failure of ACA localization.

The ACA–YFP trail data further suggest that this polarity defect alters the spatial organization of relay structures. ACA becomes enriched at the rear of polarized cells, and ACA-containing vesicles can synthesize and release cAMP (Kriebel et al., 2003, 2018). Both Ax2 and *pikG^−^* cells produced detectable ACA–YFP-positive trails, but trails deposited by *pikG^−^* cells were broader and less anisotropic than those produced by Ax2 cells (Fig. 7F–H; Movies 12 and 13). These measurements do not establish equivalent vesicle abundance or secretion between strains. Instead, they indicate that ACA-associated structures are deployed with reduced spatial coherence in the absence of PikG. Multipolar organization and disordered trail deposition could weaken the rear-directed spatial information that supports head-to-tail coupling during streaming.

The mechanism linking PikG to ACA activity and polarity remains unclear. The modest reduction in acute PH-CRAC–GFP and tPH-CynA–GFP responses might contribute to impaired relay, although the ability of *pikG^−^* cells to migrate toward an imposed cAMP gradient argues against a general failure of cAMP sensing (Fig. 2G,H, Fig. 5A–F; Fig. S4). PikG could influence ACA activation through upstream signaling, regulate trafficking or spatial organization of relay components, contribute to relay-circuit resetting, or affect a spatially restricted phosphoinositide pool not resolved by the acute whole-cell biosensor measurements. Identifying the *PikG-*generated lipid and determining whether it controls ACA activation, localization, or population-level coupling will be important next steps.

Together, the PikF and PikG phenotypes separate two kinase-dependent requirements for aggregation-stage development. PikF regulates the attenuation and spatial precision of intracellular protrusive signaling, whereas PikG supports the ACA-dependent relay architecture that generates coherent local cAMP cues. The kinase-dead rescue experiments indicate that both functions require their conserved catalytic lysines, linking these distinct developmental roles to PikF and PikG kinase-domain activity (Fig. 8). This distinction explains why *pikF^−^* cells initiate aggregation but form smaller mounds and abnormal later structures, whereas *pikG^−^* cells fail earlier, before productive streaming and aggregation.

### PikH defines an uptake-associated branch of atypical PI3K function

PikH separates uptake-associated atypical PI3K function from the developmental roles of PikF and PikG. Unlike *pikF^−^* and *pikG^−^* cells, *pikH^−^* cells resembled Ax2 in random migration, chemotaxis, development and acute cAMP-stimulated PI(3,4,5)P_3_ and PI(3,4)P_2_ dynamics (Figs 2, 3 and 5). The absence of a pronounced migration or relay phenotype argues that PikH does not contribute substantially to the acute plasma membrane signaling events measured after cAMP stimulation. Instead, *pikH^−^* cells showed reduced phagocytic uptake, whereas *pikG^−^* cells behaved similarly to Ax2 across the uptake assays (Fig. 4). PikH therefore defines a separable uptake-associated module within the atypical PI3K family.

Wild-type PikH, but not PikH^K1586A^, rescued zymosan uptake, supporting a kinase-domain-dependent role for PikH in efficient phagocytosis (Fig. S3). Given the sequence relationship of PikH to Vps34 or class III-like kinases, PikH might act at phagosomal, endosomal or trafficking-associated membranes rather than in the acute plasma membrane cAMP responses measured here. Direct localization and lipid product measurements will be required to test this model.

### Future directions

An important next step is to connect each atypical PI3K to its lipid product and site of action. The kinase-dead rescue experiments show that conserved catalytic lysines are required for *PikF-* and *PikG-*dependent development and PikH-dependent uptake (Fig. 8; Fig. S3), but they do not identify the relevant phosphoinositide products. Direct biochemical analysis, acute recruitment of kinase domains to defined membranes, live lipid biosensors and combinatorial genetics will be needed to determine which 3-phosphoinositides these enzymes generate and where those signals act. A broader unresolved question is how cell-autonomous polarity circuits are coupled to extracellular relay systems during collective migration. *Dictyostelium* aggregation requires cells to interpret cAMP gradients while also producing, polarizing and releasing cAMP cues that guide their neighbors. The separation of the PikF and PikG phenotypes provides a genetic entry point into this interface: PikF primarily affects the attenuation and spatial precision of intracellular protrusive signaling, whereas PikG affects the ACA-dependent relay architecture that allows cells to generate coherent local cues. This distinction might reflect a more general principle in amoeboid migration, where intracellular polarity and paracrine amplification must be coordinated to convert individual cell movement into collective guidance (Artemenko et al., 2014; Subramanian et al., 2017).

Together, our results argue that atypical PI3K-family enzymes divide PI3K function into separable cell biological modules. PikF constrains Ras-driven protrusive signaling, PikG supports ACA-dependent cAMP relay and its spatial organization, and PikH supports efficient phagocytic uptake. This division of labor provides a framework for understanding how phosphoinositide kinases convert local membrane signals into coordinated changes in protrusion, uptake, relay and multicellular development.

## MATERIALS AND METHODS

### Cell lines and culture

Wild-type *Dictyostelium discoideum* Ax2 cells were obtained from the DictyBase Stock Center, with Ax2 derived from DBS0235521 (Fey et al., 2013). The *pikF^−^*, *pikG^−^* and *pikH^−^* strains were generated in the Rob Kay laboratory (MRC Laboratory of Molecular Biology, Cambridge, UK) by homologous recombination with insertion of a blasticidin S resistance cassette, as described previously (Hoeller and Kay, 2007), into the isogenic DBS0235521 background. Cells were cultured axenically in HL5 medium (Formedium, UK) supplemented with vitamins and trace elements, streptomycin and ampicillin at 21.7°C on tissue-culture-treated plastic and maintained at 40–70% confluency. All live-cell experiments were performed at 21–22°C.

Cells were transformed by electroporation using an adapted DictyBase protocol (http://dictybase.org/techniques/transformation/knecht_electroporation_protocol.html) and selected in HL5 medium containing 20 μg/ml G418, 50 μg/ml hygromycin B or both, as appropriate.

Vegetative cells expressing fluorescent constructs were incubated in KK2 buffer, containing 20 mM KH_2_PO_4_ and 20 mM K_2_HPO_4_, pH 6.4, for up to 60 min before imaging to reduce HL5-associated autofluorescence and photosensitivity.

For differentiation, cells were washed twice in development buffer (DB), starved in suspension at 2×10^7^ cells/ml for 1 h, and pulsed with 100 nM cAMP every 6 min for 4–5 h. DB contained 5 mM Na_2_HPO_4_, 5 mM KH_2_PO_4_, 2 mM MgSO_4_ and 0.2 mM CaCl_2_.

### Plasmids and rescue construct generation

pDM1203-Flamindo2-linker-mScarlet was a gift from Allyson Sgro (Howard Hughes Medical Institute, Janelia Research Campus, Ashburn, VA, USA). The ACA–YFP construct was a gift from Carole Parent (University of Michigan, Ann Arbor, MI, USA). The ACA-CA–YFP construct was synthesized by GenScript by placing the open reading frame of ACA with the L394S mutation into the p88D1 backbone (Parent and Devreotes, 1996). PH-CRAC–GFP, tPH-CynA–GFP and Raf1-RBD–GFP were gifts from the Peter Devreotes laboratory (Johns Hopkins University School of Medicine, Baltimore, MD, USA). LimE–RFP was obtained from the Douglas Robinson laboratory (Johns Hopkins University School of Medicine, Baltimore, MD, USA). pDM314 and pDM317 vector backbones were obtained from DictyBase. Full-length coding sequences for *PIKF* (DDB_G0268548), *PIKG* (DDB_G0282625) and *PIKH* (DDB_G0291093) were synthesized and cloned by GenScript. Initial cloning was performed in the pDM314 backbone, and rescue constructs were subsequently generated in the GFP-tagging vector pDM317 (Veltman et al., 2009). Wild-type PikF, PikG and PikH rescue constructs were generated alongside kinase-dead variants carrying catalytic lysine-to-alanine substitutions: PikF^K1119A^, PikG^K1415A^ and PikH^K1586A^. All constructs were sequence verified.

### Sequence alignment and guide-tree visualization

Full-length PI3K protein sequences were obtained from DictyBase or UniProt and used for comparative sequence analysis. *D. discoideum* sequences included the class I PI3Ks PikA (DdPIK1; DDB_G0278727), PikB (DdPIK2; DDB_G0283081) and PikC (DdPIK3; DDB_G0275011), the class III/Vps34-like kinase PikE (DdPIK5; DDB_G0289601), and the atypical PI3K-family proteins PikF (DDB_G0268548), PikG (DDB_G0282625) and PikH (DDB_G0291093). Human comparator sequences included class I PI3Kα (PIK3CA; UniProt P42336), PI3Kβ (PIK3CB; UniProt P42338) and PI3Kγ (PIK3CG; UniProt P48736), class II PI3K-C2α (PIK3C2A; UniProt O00443), PI3K-C2β (PIK3C2B; UniProt O00750) and PI3K-C2γ (PIK3C2G; UniProt O75747), and class III VPS34 (PIK3C3; UniProt Q8NEB9). Protein sequences were aligned using the Clustal Omega web server (https://www.ebi.ac.uk/jdispatcher/msa/clustalo) hosted by the European Molecular Biology Laboratory-European Bioinformatics Institute. The Clustal Omega guide tree was exported and visualized using FigTree (https://tree.bio.ed.ac.uk/software/figtree/).

### Random migration assay

Vegetative cells were harvested during exponential growth, centrifuged at 300 ***g*** for 4 min and resuspended in KK2 buffer at 1×10^7^ cells/ml. Cells were plated in the center of one-well chambered coverglass dishes, allowed to adhere briefly and maintained in KK2 buffer. Random migration was recorded by phase-contrast imaging every 15 s for 1 h on a Nikon Eclipse Ti2-E microscope using a 10× 0.55 NA Plan Achromat objective. Random migration was analyzed in FIJI/ImageJ using the MTrackJ plugin (https://imagescience.org/meijering/software/mtrackj/). Individual cell centroids were manually tracked at 120 s intervals over the imaging period. Cell trajectories were used to calculate total path length, net displacement, migration speed and directional persistence, defined as net displacement divided by total path length.

### Micropipette chemotaxis assay

Developed cells were plated in DB at approximately 5×10^5^ cells/ml in one-well coverslip chambers (Nunc Lab-Tek). Glass capillaries (BF100-58-10; Sutter Instruments) were pulled to a 0.8–1.5 μm tip and filled with 10 μM cAMP. cAMP gradients were generated by allowing a slow leak from the capillary under 10–20 hPa backpressure using a FemtoJet 4i microinjector (5252000021, Calibre Scientific, San Diego, CA, USA). Chemotaxis toward the micropipette was imaged by phase contrast on a Nikon Eclipse Ti2-E microscope using either a 10× 0.55 NA or 40× oil 1.4 NA objective. For buffer-control assays, micropipettes containing buffer without cAMP were positioned adjacent to developed cells under otherwise identical imaging conditions.

Chemotactic alignment was quantified in FIJI/ImageJ. For each time interval, we calculated the angular deviation θ between the ideal path vector, defined as the straight-line direction toward the chemoattractant source, and the actual step vector, defined by the cell displacement between consecutive frames. Angular deviations were summarized using circular variance, calculated as 1−R, where R is the mean resultant vector length of the step-angle distribution. Lower circular variance indicates that sequential cell steps were consistently aligned with the ideal path, whereas higher circular variance indicates more variable step directions relative to the ideal path.

### Development on non-nutrient agar

Developmental progression was assayed on 1% non-nutrient KK2 agar. For each strain, 1×10^7^ cells were harvested during exponential growth, centrifuged at 300 ***g*** for 4 min at 22°C, and washed three times with KK2 buffer. Cells were resuspended in 1 ml KK2 buffer, plated as a single central spot and allowed to dry before imaging in a humidified chamber. Time-lapse images were acquired every 10 min on a Leica M165 FC stereomicroscope at 7.3× magnification. Standard developmental and PikF/PikG kinase-rescue experiments were imaged for 48 h. Experiments comparing Ax2 cells, *pikG^−^* cells, *pikG^−^* cells expressing ACA-CA–YFP and *pikG^−^* cells expressing wild-type ACA–YFP were imaged for 30 h under otherwise identical conditions.

Mound emergence and progression to fruiting body formation were scored from time-lapse image sequences using FIJI/ImageJ. Mounds were identified manually as discrete multicellular aggregates. Time to 10% mound emergence was defined as the first time point at which 10% of the total mounds observed within that experiment had formed. Approximately 30 mounds per plate were selected for developmental-stage scoring.

Developmental endpoints in Fig. 8 were analyzed using mixed-effects models with strain or rescue construct as a fixed effect and biological replicate as a random effect, followed by Tukey's multiple-comparisons test. Adjusted *P*-values are reported in the figure.

### Phagocytosis assay

For bead phagocytosis assays, 4.5 μm blue fluorescent carboxylate microspheres (Polysciences) were washed in KK2 buffer containing 5% BSA and added to cells at 50 beads per cell. Phagocytosis was stopped at the indicated time points by addition of ice-cold KK2 buffer containing 5% BSA, and cells were washed to remove uningested beads.

For zymosan phagocytosis assays, cells were harvested during exponential growth, washed twice in KK2 buffer, resuspended in KK2 and shaken for 60 min before addition of TRITC-labeled zymosan particles (Thermo Fisher Scientific). Phagocytosis was stopped at the indicated time points by addition of ice-cold KK2, and cells were washed to remove uningested particles before imaging.

For both assays, cells were imaged by brightfield and epifluorescence microscopy on a Nikon Eclipse Ti2-E microscope using a 40× 1.4 NA objective. Internalized particles were identified from paired brightfield and fluorescence images.

Uptake time courses were analyzed in GraphPad Prism using mixed-effects models with strain and time as factors. Models were followed by Tukey's multiple-comparisons test, with comparisons of each mutant strain with Ax2 reported in Fig. 4D. Adjusted *P*-values are reported for significant comparisons, and comparisons that were not significant after correction are designated as n.s.

### Macropinocytosis assay

Cells were washed twice in KK2 buffer and starved for 30 min with shaking. Cells were plated in Nunc Lab-Tek II eight-well chambered coverglass slides and allowed to adhere briefly before addition of Alexa Fluor 647–dextran (D22914, Invitrogen). Dextran uptake was imaged every 1 min for 1 h on a Zeiss LSM 980 laser scanning confocal microscope with Airyscan 2 using a 40× 1.4 NA objective.

Macropinocytosis was quantified in FIJI/ImageJ by measuring intracellular Alexa Fluor 647–dextran fluorescence intensity over time. Mean fluorescence intensity was plotted over time, and statistical analysis was performed in GraphPad Prism. Macropinocytic uptake time courses were analyzed in GraphPad Prism as described in the phagocytosis assay.

### Uniform cAMP stimulation assay

Cells were developed as described above, plated at 5×10^4^–1×10^5^ cells per well in eight-well chambered coverglass slides (Nunc Lab-Tek), and allowed to adhere for 10 min before imaging. To minimize stimulus-induced shape changes, cells were treated with 5 μM latrunculin A (428021, Millipore Sigma) for 5 min. Cells were imaged on a Zeiss LSM 980 laser scanning confocal microscope with Airyscan 2 using a 40× 1.4 NA objective and stimulated with 10 μM cAMP during image acquisition. Biosensor redistribution was imaged every 2 s before and after stimulation. This assay was used to quantify cAMP-stimulated dynamics of PH-CRAC–GFP, tPH-CynA–GFP and Raf1-RBD–GFP.

Biosensor redistribution was quantified in FIJI/ImageJ as the membrane-to-cytosol fluorescence ratio. Membrane and cytosolic regions were defined from binary masks, background-subtracted fluorescence was measured in each region, and membrane intensity was divided by cytosolic intensity at each time point. Ratios were normalized to the pre-stimulation baseline, defined as the average of five pre-stimulation frames, where indicated. Peak response was defined as the maximum absolute deviation from baseline after cAMP addition. For PH-CRAC–GFP, recovery time was defined as the first post-peak time point at which the ratio returned to baseline. For tPH-CynA–GFP, time to 50% recovery was defined as the first post-peak time point at which the signal recovered halfway from peak response toward baseline. Recovery times were reported relative to cAMP stimulation rather than the start of image acquisition. Recovery distributions were analyzed by Kaplan–Meier survival analysis with log-rank comparisons. Peak amplitudes were compared by Kruskal–Wallis test with Dunn's multiple-comparisons correction.

### Lipid extraction and PIP Mass ELISA

Developed cells were collected, resuspended in DB at 5×10^7^ cells/ml, and stimulated with 10 μM cAMP for the indicated times or treated with DB for unstimulated controls. Stimulations were stopped by addition of ice-cold 0.7 M trichloroacetic acid.

Lipids were isolated by sequential acidic extraction. Cell pellets were washed in 5% trichloroacetic acid containing 1 mM EDTA, neutral lipids were removed with methanol:chloroform, and acidic lipids were extracted with methanol:chloroform:HCl. Organic phases were collected, dried by a SpeedVac, and stored at −20°C until analysis.

PI(3,4,5)P_3_, PI(3,4)P_2_ and PI3P levels were measured using Echelon PIP Mass ELISA kits according to the manufacturer's instructions. Dried lipid extracts were resuspended in the supplied assay buffer, sonicated and assayed in duplicate alongside purified PIP standards. Absorbance was measured at 450 nm using a SpectraMax plate reader, and lipid abundance was calculated from standard curves. Lipid abundance was normalized to cell number for each extraction. For time-course stimulation experiments, fold change at each time point was calculated relative to the unstimulated 0 s value from the same independent extraction.

Five independent lipid extractions were analyzed for the cAMP-stimulated PI(3,4,5)P_3_ time course. Samples from each extraction were assayed in duplicate, and fold change at each time point was calculated relative to the unstimulated value for the same strain and extraction. Time-course data were analyzed in GraphPad Prism using a mixed-effects model with strain, time and strain×time as fixed effects and independent extraction as a random effect. Dunnett's multiple-comparisons test was used to compare each mutant strain with Ax2 at each time point. Adjusted *P*-values are reported in Fig. S5E.

### Peripheral actin imaging

Vegetative Ax2 and *pikF^−^* cells expressing LimE–RFP were plated in chambered coverglass dishes and imaged for 500 s on a Zeiss LSM 980 laser scanning confocal microscope with Airyscan 2 using a 40× 1.4 NA objective to monitor cortical actin dynamics. Peripheral LimE–RFP patches were quantified from time-lapse images in FIJI/ImageJ. Cortical fluorescence enrichments were scored over time, and the number of peripheral actin patches per cell was recorded.

### Ras crescent assay

Raf1-RBD–GFP-expressing cells were developed as described above. Cells were resuspended in KK2, plated in chambered coverglass dishes, and treated with 5 μM latrunculin A and 1 mM caffeine before stimulation. Cells were stimulated with a micropipette containing either 10 μM cAMP or 0 μM cAMP control positioned adjacent to the cells. Raf1-RBD–GFP dynamics were imaged every 2 s on a Nikon Eclipse Ti2-E microscope equipped with a C2 confocal module using a 63× 1.4 NA objective.

Ras crescent orientation was measured in FIJI/ImageJ to quantify how closely Ras activation was oriented toward the cAMP source. For each frame, a reference line representing the most direct path to the needle was drawn from the cell centroid to the cAMP-filled micropipette tip. Each discrete Raf1-RBD–GFP-enriched cortical crescent was identified, and its position was defined by the crescent centroid. The angle φ between each crescent centroid and the reference line to the needle was measured. When multiple crescents were present in a single cell, each crescent was measured separately and included as a crescent-level angular measurement.

### Flamindo2 oscillatory response assay

Ax2, *pikG^−^* and *aca*^−^ cells expressing Flamindo2–mScarlet were harvested during exponential growth, washed twice in KK2 buffer and plated on ultrathin non-nutrient agar at 5×10^6^ cells/ml. Cells were developed under humidified, sealed imaging conditions and imaged on a Nikon Eclipse Ti2-E microscope at 100× magnification. Images were acquired every 60 s for 8 h.

For Ax2 cells, fields containing aggregation centers were analyzed beginning 60 min before visible streaming. Because *pikG^−^* and *aca*^−^ cells did not form aggregation centers, fields were selected randomly by an observer masked to experimental condition, and analysis began 4 h after starvation.

Mean Flamindo2–mScarlet fluorescence intensity was measured over time in FIJI/ImageJ using the Time Series Analyzer plugin. Intensity traces were fitted in GraphPad Prism using an oscillatory function to estimate amplitude and period, with goodness of fit assessed by R^2^. Oscillation periods were also estimated using a custom Python-based autocorrelation analysis (https://github.com/maedwards86-a11y/Autocorrelation-analysis/). Fluorescence traces were mean-centered, autocorrelated and normalized to the zero-lag value. The oscillation period was defined as the time lag of the first non-zero autocorrelation peak. Traces without a detectable autocorrelation peak were classified as non-oscillatory.

### ACA–YFP polarity assay

Developed Ax2 and *pikG^−^* cells expressing ACA–YFP were plated on 13 mm borosilicate coverslips and fixed in 4% paraformaldehyde in PBS. Cells were imaged on a Zeiss LSM 980 laser scanning confocal microscope with Airyscan 2 using a 60× 1.4 NA objective. ACA–YFP polarity was quantified from fluorescence intensity profiles along the cell perimeter. An ACA–YFP pole was defined operationally as a cortical enrichment that spanned at least 10% of the total cell perimeter, was separated from neighboring poles by at least 10% of the perimeter, remained at least threefold above the minimum perimeter intensity, and reached at least 50% of the maximum perimeter intensity.

### Under-agar two-drop chemotaxis and cellular polarity assay

Ax2 and *pikG*^−^ cells expressing tPH-CynA–GFP were developed as described above. Developed cells were resuspended in KK2 at 7×10^6^ cells/ml, and a 5 μl drop was plated in a Nunc Lab-Tek II two-well chambered coverglass dish and allowed to adhere. Thin films of 1.5% KK2 agar were prepared in 6-cm tissue-culture dishes by adding a minimal volume of molten agar and rotating the dish until the agar formed a continuous thin layer. Sections of the agar film were placed over the adherent cells to gently compress them against the coverglass.

A 5 μl drop of 10 μM cAMP was placed 5–10 mm from the edge of the spread cells to establish an under-agar chemoattractant gradient. Cells migrating toward the cAMP source were imaged every 10 s on a Zeiss LSM 980 laser scanning confocal microscope with Airyscan 2 using a 40× 1.4 NA objective.

Cells were classified using the same unipolar, multipolar and nonpolar categories used for the ACA–YFP polarity analysis. Cells were scored as unipolar when they maintained one dominant cellular front or polarity axis, multipolar when they displayed two or more spatially separated fronts, and nonpolar when no stable front could be identified. The proportion of cells in each category was calculated separately for each biological replicate. Thirty cells per strain were scored in each of three independent experiments. The proportions of unipolar and multipolar cells were compared between paired Ax2 and pikG^−^ experiments using paired two-tailed *t*-tests with Holm–Šídák correction for the two planned comparisons. Nonpolar cells were not included in the statistical comparisons.

### ACA–YFP vesicle trail directionality and anisotropy assay

Developed Ax2 and *pikG^−^* cells expressing ACA–YFP were plated at 5×10^4^ cells/well in 500 μl DB in Nunc Lab-Tek II chambered coverglass slides. Cells were imaged every 10 s for 10 min on a Zeiss LSM 980 laser scanning confocal microscope with Airyscan 2 using a 40× 1.4 NA objective. Vesicle *x-y* coordinates were recorded in order of secretion. Vesicle trail directionality is a persistence measure calculated as net displacement divided by total path length. Vesicle trail anisotropy was calculated from the variance in vesicle position along the *x*- and *y*-axes (VarX and VarY) and the *x-y* covariance (CovXY). Eigenvalues were calculated as

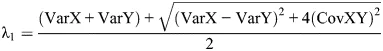
and

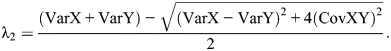


These were used in the anisotropy equation: 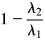, where values closest to 1 represent the most anisotropic trails and values closest to 0 represent the most isotropic trails.

### Membrane kymograph generation

Where membrane kymographs are shown, cell masks were generated from fluorescence images in FIJI/ImageJ and used to define cell boundaries for kymograph generation with Membrane Kymograph Generator (Banerjee et al., 2026).

### Developmental immunoblotting and densitometry

Ax2 and *pikG^−^* cells were developed as described above. Samples were collected before development and at 2, 4, 6 and 8 h after the initiation of development. Whole-cell lysates were prepared at each time point and analyzed by SDS-PAGE and immunoblotting using antibodies against ACA (a gift from Peter Devreotes, 1:1000), cAR1 (a gift from Peter Devreotes, 1:100) and csA (Developmental Studies Hybridoma Bank, 33-294-17, 1:1000). The anti-cAR1 antibody was pre-cleared against fixed vegetative cells (Parent and Devreotes, 1995; Hereld et al., 1994). α-Tubulin was detected using an antibody (Developmental Studies Hybridoma Bank, 12G10, 1:2000) in the corresponding samples as a loading control.

Band intensities were quantified by densitometry. For each lane, the background-corrected intensity of the target protein band was divided by the intensity of the corresponding α-tubulin band. To place independent experiments on a common scale while retaining differences between Ax2 and *pikG^−^* cells, each target-to-tubulin ratio was divided by the Ax2 target-to-tubulin ratio at 8 h from the same biological replicate and multiplied by 100. The Ax2 8 h value was therefore defined as 100% for each protein and replicate. Normalized values are reported as mean±s.d. from three independent biological replicates.

### Statistical analysis

Statistical analyses were performed in GraphPad Prism. Statistical tests were selected based on the experimental design and are indicated in the corresponding figure legends. For comparisons among multiple strains, Kruskal–Wallis tests with Dunn's multiple-comparisons correction or mixed-effects models were used as indicated. Circular data were analyzed using circular variance. *P*-values less than 0.05 were considered statistically significant. Sample sizes, replicate numbers, definitions of *n* and error bars are reported in the figure legends.

## Supplementary Material

10.1242/joces.265041_sup1 Supplementary information

## References

[JCS265041C1] Andrew, N. and Insall, R. H. (2007). Chemotaxis in shallow gradients is mediated independently of PtdIns 3-kinase by biased choices between random protrusions. *Nat. Cell Biol.* 9, 193-200. 10.1038/ncb1536 17220879

[JCS265041C2] Artemenko, Y., Lampert, T. J. and Devreotes, P. N. (2014). Moving towards a paradigm: common mechanisms of chemotactic signaling in Dictyostelium and mammalian leukocytes. *Cell. Mol. Life Sci.* 71, 3711-3747. 10.1007/s00018-014-1638-8 PMC4162842 24846395

[JCS265041C3] Balla, T. (2013). Phosphoinositides: tiny lipids with giant impact on cell regulation. *Physiol. Rev.* 93, 1019-1137. 10.1152/physrev.00028.2012 PMC3962547 23899561

[JCS265041C43] Banerjee, T., Abubaker-Sharif, B., Devreotes, P. N. and Iglesias, P. A. (2026). Membrane Kymograph Generator: a cross-platform GUI software for automated generation and analysis of kymographs along dynamic cell boundaries. *Bioinformatics* 42, btag400. 10.1093/bioinformatics/btag400 PMC13310011 42316814

[JCS265041C4] Brown, J. R. and Auger, K. R. (2011). Phylogenomics of phosphoinositide lipid kinases: perspectives on the evolution of second messenger signaling and drug discovery. *BMC Evol. Biol.* 11, 4. 10.1186/1471-2148-11-4 PMC3024228 21208444

[JCS265041C5] Charest, P. G. and Firtel, R. A. (2006). Feedback signaling controls leading-edge formation during chemotaxis. *Curr. Opin. Genet. Dev.* 16, 339-347. 10.1016/j.gde.2006.06.016 16806895

[JCS265041C6] Comer, F. I. and Parent, C. A. (2006). Phosphoinositide 3-kinase activity controls the chemoattractant-mediated activation and adaptation of adenylyl cyclase. *Mol. Biol. Cell* 17, 357-366. 10.1091/mbc.e05-08-0781 PMC1345673 16267269

[JCS265041C7] Devreotes, P. N., Bhattacharya, S., Edwards, M., Iglesias, P. A., Lampert, T. and Miao, Y. (2017). Excitable signal transduction networks in directed cell migration. *Annu. Rev. Cell Dev. Biol.* 33, 103-125. 10.1146/annurev-cellbio-100616-060739 PMC5792054 28793794

[JCS265041C8] Dormann, D. and Weijer, C. J. (2003). Chemotactic cell movement during development. *Curr. Opin. Genet. Dev.* 13, 358-364. 10.1016/s0959-437x(03)00087-x 12888008

[JCS265041C9] Dormann, D., Weijer, G., Parent, C. A., Devreotes, P. N. and Weijer, C. J. (2002). Visualizing PI3 kinase-mediated cell-cell signaling during Dictyostelium development. *Curr. Biol.* 12, 1178-1188. 10.1016/s0960-9822(02)00950-8 12176327

[JCS265041C10] Engelman, J. A., Luo, J. and Cantley, L. C. (2006). The evolution of phosphatidylinositol 3-kinases as regulators of growth and metabolism. *Nat. Rev. Genet.* 7, 606-619. 10.1038/nrg1879 16847462

[JCS265041C11] Ferguson, G. J., Milne, L., Kulkarni, S., Sasaki, T., Walker, S., Andrews, S., Crabbe, T., Finan, P., Jones, G., Jackson, S. et al. (2007). PI(3)Kγ has an important context-dependent role in neutrophil chemokinesis. *Nat. Cell Biol.* 9, 86-91. 10.1038/ncb1517 17173040

[JCS265041C44] Fey, P., Dodson, R. J., Basu, S. and Chisholm, R. L. (2013). One stop shop for everything Dictyostelium: dictyBase and the Dicty Stock Center in 2012. *Methods. Mol. Biol.* 983, 59-92. 10.1007/978-1-62703-302-2_4 PMC3762881 23494302

[JCS265041C12] Friedl, P. and Wolf, K. (2010). Plasticity of cell migration: a multiscale tuning model. *J. Cell Biol.* 188, 11-19. 10.1083/jcb.200909003 PMC2812848 19951899

[JCS265041C13] Funamoto, S., Meili, R., Lee, S., Parry, L. and Firtel, R. A. (2002). Spatial and temporal regulation of 3-phosphoinositides by PI 3-kinase and PTEN mediates chemotaxis. *Cell* 109, 611-623. 10.1016/S0092-8674(02)00755-9 12062104

[JCS265041C14] Garcia, G. L. and Parent, C. A. (2008). Signal relay during chemotaxis. *J. Microsc.* 231, 529-534. 10.1111/j.1365-2818.2008.02066.x PMC2745292 18755009

[JCS265041C15] Gulluni, F., De Santis, M. C., Margaria, J. P., Martini, M. and Hirsch, E. (2019). Class II PI3K functions in cell biology and disease. *Trends Cell Biol.* 29, 339-359. 10.1016/j.tcb.2019.01.001 30691999

[JCS265041C16] Hashimura, H., Morimoto, Y. V., Yasui, M. and Ueda, M. (2019). Collective cell migration of Dictyostelium without cAMP oscillations at multicellular stages. *Commun. Biol.* 2, 34. 10.1038/s42003-018-0273-6 PMC6345914 30701199

[JCS265041C17] Hawkins, P. T. and Stephens, L. R. (2016). Emerging evidence of signalling roles for PI(3,4)P2 in class I and II PI3K-regulated pathways. *Biochem. Soc. Trans.* 44, 307-314. 10.1042/BST20150248 26862220

[JCS265041C48] Hereld, D., Vaughan, R., Kim, J. Y., Borleis, J. and Devreotes, P. (1994). Localization of ligand-induced phosphorylation sites to serine clusters in the C-terminal domain of the Dictyostelium cAMP receptor, cAR1. *J. Biol. Chem.* 269, 7036-7044. 10.1016/S0021-9258(17)37479-3 8120068

[JCS265041C18] Hoeller, O. and Kay, R. R. (2007). Chemotaxis in the absence of PIP3 gradients. *Curr. Biol.*, 17, 813-817. 10.1016/j.cub.2007.04.004 17462897

[JCS265041C19] Hoeller, O., Bolourani, P., Clark, J., Stephens, L. R., Hawkins, P. T., Weiner, O. D., Weeks, G. and Kay, R. R. (2013). Two distinct functions for PI3-kinases in macropinocytosis. *J. Cell Sci.* 126, 4296-4307. 10.1242/jcs.134015 PMC3772393 23843627

[JCS265041C20] Iijima, M. and Devreotes, P. (2002). Tumor suppressor PTEN mediates sensing of chemoattractant gradients. *Cell* 109, 599-610. 10.1016/S0092-8674(02)00745-6 12062103

[JCS265041C21] Kimmel, A. R. and Parent, C. A. (2003). The signal to move: D. discoideum go orienteering. *Science* 300, 1525-1527. 10.1126/science.1085439 12791977

[JCS265041C45] King, J. S. and Kay, R. R. (2019). The origins and evolution of macropinocytosis. *Philos. Trans. R. Soc. Lond. B. Biol. Sci.* 374, 20180158. 10.1098/rstb.2018.0158 PMC6304743 30967007

[JCS265041C22] Kriebel, P. W., Barr, V. A. and Parent, C. A. (2003). Adenylyl cyclase localization regulates streaming during chemotaxis. *Cell* 112, 549-560. 10.1016/S0092-8674(03)00081-3 12600317

[JCS265041C23] Kriebel, P. W., Majumdar, R., Jenkins, L. M., Senoo, H., Wang, W., Ammu, S., Chen, S., Narayan, K., Iijima, M. and Parent, C. A. (2018). Extracellular vesicles direct migration by synthesizing and releasing chemotactic signals. *J. Cell Biol.* 217, 2891-2910. 10.1083/jcb.201710170 PMC6080930 29884750

[JCS265041C24] Lam, P., Yoo, S. K., Green, J. M. and Huttenlocher, A. (2012). The SH2-domain-containing inositol 5-phosphatase (SHIP) limits neutrophil motility and wound recruitment in zebrafish. *J. Cell Sci.* 125, 4973-4978. 10.1242/jcs.106625 PMC3533387 22946052

[JCS265041C25] Li, X., Edwards, M., Swaney, K. F., Singh, N., Bhattacharya, S., Borleis, J., Long, Y., Iglesias, P. A., Chen, J. and Devreotes, P. N. (2018). Mutually inhibitory Ras-PI(3,4)P(2) feedback loops mediate cell migration. *Proc. Natl. Acad. Sci. USA* 115, E9125-E9134. 10.1073/pnas.1809039115 PMC6166812 30194235

[JCS265041C26] Li, X., Pal, D. S., Biswas, D., Iglesias, P. A. and Devreotes, P. N. (2021). Reverse fountain flow of phosphatidylinositol-3,4-bisphosphate polarizes migrating cells. *EMBO J.* 40, e105094. 10.15252/embj.2020105094 PMC7883298 33586225

[JCS265041C27] Loomis, W. F. (2014). Cell signaling during development of Dictyostelium. *Dev. Biol.* 391, 1-16. 10.1016/j.ydbio.2014.04.001 PMC4075484 24726820

[JCS265041C28] Loovers, H. M., Kortholt, A., de Groote, H., Whitty, L., Nussbaum, R. L. and van Haastert, P. J. M. (2007). Regulation of phagocytosis in Dictyostelium by the inositol 5-phosphatase OCRL homolog Dd5P4. *Traffic* 8, 618-628. 10.1111/j.1600-0854.2007.00546.x 17343681

[JCS265041C29] Nishio, M., Watanabe, K.-, Sasaki, J., Taya, C., Takasuga, S., Iizuka, R., Balla, T., Yamazaki, M., Watanabe, H., Itoh, R. et al. (2007). Control of cell polarity and motility by the PtdIns(3,4,5)P3 phosphatase SHIP1. *Nat. Cell Biol.* 9, 36-44. 10.1038/ncb1515 17173042

[JCS265041C30] Pal, D. S., Li, X., Banerjee, T., Miao, Y. and Devreotes, P. N. (2019). The excitable signal transduction networks: movers and shapers of eukaryotic cell migration. *Int. J. Dev. Biol.* 63, 407-416. 10.1387/ijdb.190265pd PMC6956983 31840779

[JCS265041C49] Parent, C. A. and Devreotes, P. N. (1995). Isolation of inactive and G protein-resistant adenylyl cyclase mutants using random mutagenesis. *J. Biol. Chem.* 270, 22693-22696. 10.1074/jbc.270.39.22693 7559391

[JCS265041C31] Parent, C. A. and Devreotes, P. N. (1996). Constitutively active adenylyl cyclase mutant requires neither G proteins nor cytosolic regulators. *J. Biol. Chem.*, 271, 18333-18336. 10.1074/jbc.271.31.18333 8702473

[JCS265041C32] Pirola, L., Zvelebil, M. J., Bulgarelli-Leva, G., Van Obberghen, E., Waterfield, M. D. and Wymann, M. P. (2001). Activation loop sequences confer substrate specificity to phosphoinositide 3-kinase α (PI3Kα). *J. Biol. Chem.* 276, 21544-21554. 10.1074/jbc.M011330200 11278889

[JCS265041C33] Posor, Y., Eichhorn-Gruenig, M., Puchkov, D., Schöneberg, J., Ullrich, A., Lampe, A., Müller, R., Zarbakhsh, S., Gulluni, F., Hirsch, E. et al. (2013). Spatiotemporal control of endocytosis by phosphatidylinositol-3,4-bisphosphate. *Nature* 499, 233-237. 10.1038/nature12360 23823722

[JCS265041C34] Postma, M., Roelofs, J., Goedhart, J., Loovers, H. M., Visser, A. J. W. G. and Van Haastert, P. J. M. (2004). Sensitization of Dictyostelium chemotaxis by phosphoinositide-3-kinase-mediated self-organizing signalling patches. *J. Cell Sci.* 117, 2925-2935. 10.1242/jcs.01143 15161938

[JCS265041C35] Ridley, A. J., Schwartz, M. A., Burridge, K., Firtel, R. A., Ginsberg, M. H., Borisy, G., Parsons, J. T. and Horwitz, A. R. (2003). Cell migration: integrating signals from front to back. *Science* 302, 1704-1709. 10.1126/science.1092053 14657486

[JCS265041C36] Subramanian, B. C., Majumdar, R. and Parent, C. A. (2017). The role of the LTB 4 -BLT1 axis in chemotactic gradient sensing and directed leukocyte migration. *Semin. Immunol.* 33, 16-29. 10.1016/j.smim.2017.07.002 PMC8288505 29042024

[JCS265041C37] Swaney, K. F., Huang, C.-H. and Devreotes, P. N. (2010). Eukaryotic chemotaxis: a network of signaling pathways controls motility, directional sensing, and polarity. *Annu. Rev. Biophys.* 39, 265-289. 10.1146/annurev.biophys.093008.131228 PMC4364543 20192768

[JCS265041C38] Swaney, K. F., Borleis, J., Iglesias, P. A. and Devreotes, P. N. (2015). Novel protein Callipygian defines the back of migrating cells. *Proc. Natl. Acad. Sci. U.S.A.* 112, E3845-E3854. 10.1073/pnas.1509098112 PMC4517219 26130809

[JCS265041C46] Veltman, D. M., Keizer-Gunnink, I. and Haastert, P. J. (2009). An extrachromosomal, inducible expression system for Dictyostelium discoideum. *Plasmid* 61, 119-125. 10.1016/j.plasmid.2008.11.002 19046986

[JCS265041C39] Wallroth, A. and Haucke, V. (2018). Phosphoinositide conversion in endocytosis and the endolysosomal system. *J. Biol. Chem.* 293, 1526-1535. 10.1074/jbc.R117.000629 PMC5798284 29282290

[JCS265041C40] Weijer, C. J. (2004). Dictyostelium morphogenesis. *Curr. Opin. Genet. Dev.* 14, 392-398. 10.1016/j.gde.2004.06.006 15261655

[JCS265041C41] Yamada, K. M. and Sixt, M. (2019). Mechanisms of 3D cell migration. *Nat. Rev. Mol. Cell Biol.* 20, 738-752. 10.1038/s41580-019-0172-9 31582855

[JCS265041C42] Zhou, K., Takegawa, K., Emr, S. D. and Firtel, R. A. (1995). A phosphatidylinositol (PI) kinase gene family in Dictyostelium discoideum: biological roles of putative mammalian p110 and yeast Vps34p PI 3-kinase homologs during growth and development. *Mol. Cell. Biol.* 15, 5645-5656. 10.1128/mcb.15.10.5645 PMC230815 7565716

