# Supplementary material for: Atypical PI3Ks coordinate chemotaxis, signaling dynamics and multicellular development in Dictyostelium
Source: J Cell Sci. 2026 Oct 2;139(18):jcs265041. doi: 10.1242/jcs.265041 (PMC13676897; doi:10.1242/jcs.265041)
Supplement: Supplementary file 1 [file joces-139-265041-s1.pdf]

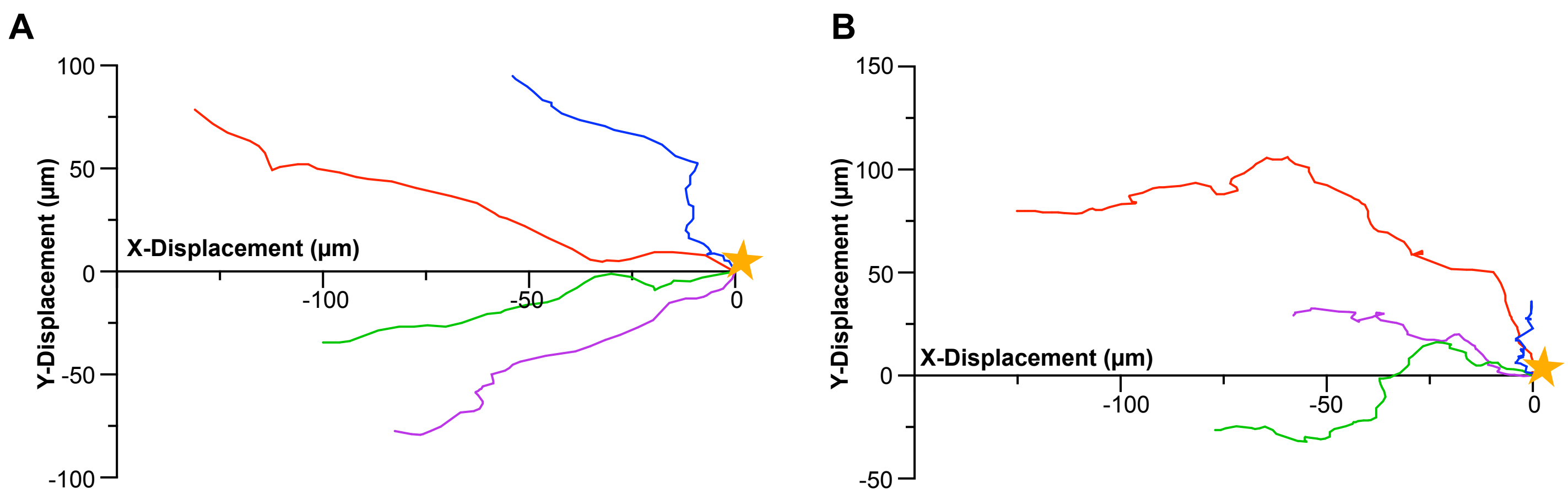


**Fig. S1. Representative directed migration trajectories of Ax2 cells and *pikF*⁻ cells.**
**(A)** Four representative Ax2 cell trajectories from the directed cell migration assay. **(B)** Four representative *pikF*⁻ cell trajectories from the same assay.
These trajectories are from the same cells analyzed in Fig. 2G. The gold star indicates the position of the microcapillary needle.

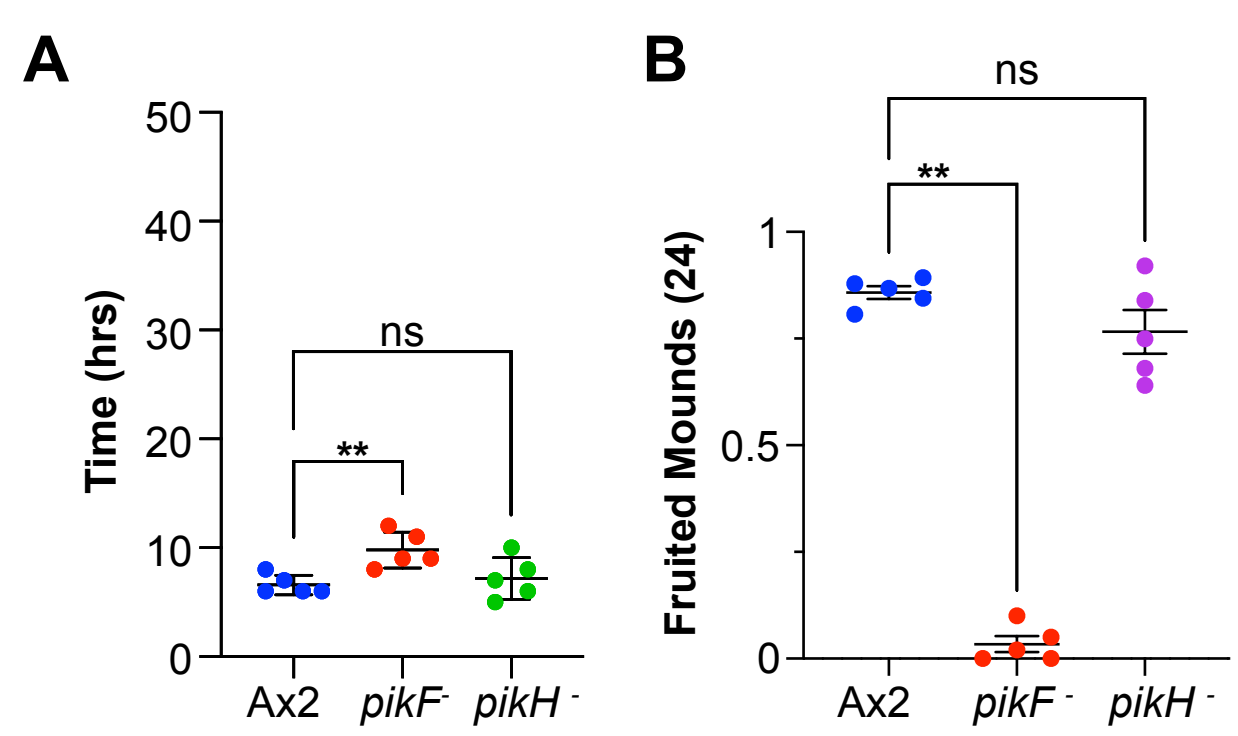


**Fig. S2. Quantification of developmental timing in Ax2 and atypical PI3K mutants.**
**(A)** Time to emergence of 10% of total mounds formed by Ax2 cells, *pikF*⁻ cells, *pikG*⁻ cells, and *pikH*⁻ cells developing on non-nutrient agar. Five biological replicates were analyzed.
**(B)** Fraction of mounds reaching the fruiting-body stage by 24 h. Five biological replicates were analyzed, with approximately 30 mounds scored per plate. Bars indicate mean ± s.e.m. Statistical significance was assessed by Kruskal-Wallis test; *P < 0.001 where indicated.

Journal of Cell Science • Supplementary information

A

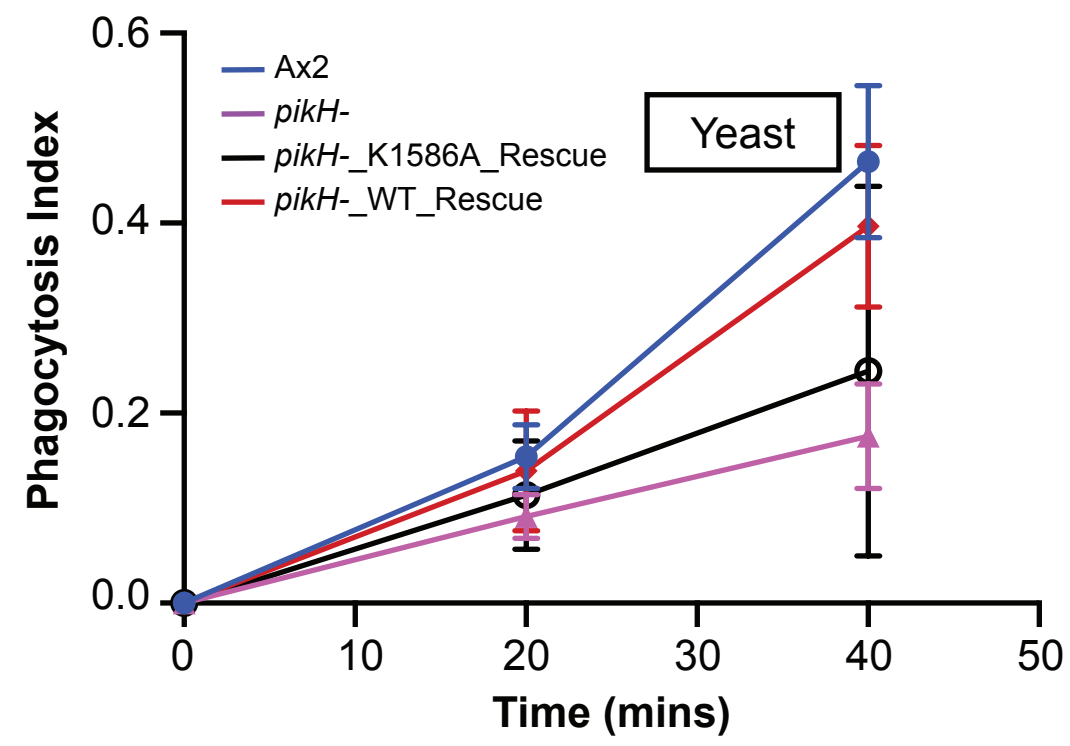


B

| ***pikH-* Kinase Dead Uptake Assay** | | | | |
|---|---|---|---|---|
| *pikH-* vs Ax2 | *pikH- vs* *pikH-*_WT Rescue | *pikH-_K1586A-* vs *pikH-*_WT Rescue | *pikH-_K1586A-* vs *Ax2* | *pikH-_K1586A-* vs *pikH-* |
| *p<.0001 | *p<.01 | *p<.01 | *p<.001 | n.s. |

**Fig. S3. Wild-type, but not kinase-dead, PikH rescues the phagocytic uptake defect in *pikH⁻* cells.**

**(A)** Phagocytosis index for uptake of fluorescent *Saccharomyces cerevisiae* particles by the indicated strains. Re-expression of wild-type PikH restores uptake in *pikH*$^-$ cells, whereas the kinase-dead PikH^K1586A allele fails to rescue the uptake defect. Bars indicate mean ± s.e.m. from two biological replicates.

**(B)** Statistical summary of the uptake kinetics shown in A. Time courses were analyzed using a mixed-effects model with strain, time and strain × time as fixed effects and biological replicate as a random effect, followed by Dunnett's multiple-comparisons test comparing Ax2 cells and each rescue strain with *pikH*$^-$cells. Adjusted P value thresholds are indicated; n.s., not significant.

| **Table2. Biosensor Stimulations Analysis** | | | | |
|---|---|---|---|---|
| **A** | YFP-PH-CRAC | | **B** KIKGR-PH-CynA | |
| Strains | Peak Response a.u. | Return to Baseline (s) | Peak Response a.u. | Return to Baseline (s) |
| Ax2 | 6.02 ± 1.23 | 24.60 ± 3.50 | 0.547 ± 0.093 | 91.0 ± 21.8 |
| *pikF-* | 5.88 ± 2.12 | 29.95 ± 6.24 ** | 0.557 ± 0.082 | 128.0 ± 15.0 ** |
| *pikG-* | 4.57 ± 0.94 * | 25.91 ± 3.45 | 0.702 ± 0.111 * | 102.8 ± 20.5 |
| *pikH-* | 6.14 ± 1.51 | 24.02 ± 3.51 | 0.577 ± 0.070 | 84.3 ± 16.6 |

**Fig. S4. Quantification of PH-CRAC-GFP and tPH-CynA-GFP responses shown in Fig. 5**.

**(A)** Quantification of PH-CRAC-GFP peak response and time to return to pre-stimulation baseline in Ax2 cells, *pikF*$^-$cells, *pikG*$^-$cells, and *pikH*$^-$ cells following uniform cAMP stimulation, based on the data shown in Fig. 5C.

**(B)** Quantification of tPH-CynA-GFP peak response and time to 50% recovery in Ax2 cells, *pikF*$^-$cells, *pikG*$^-$cells, and *pikH*$^-$cells following uniform cAMP stimulation, based on the data shown in Fig. 5F. n = 60 cells per strain from three biological replicates, with 20 cells analyzed per replicate. Recovery times in S4 are reported as time after cAMP stimulation, whereas traces in Fig. 5C,F are plotted on the full acquisition timeline with cAMP added at 10 s. Peak amplitudes were compared by Kruskal–Wallis test with Dunn's post hoc correction. PH-CRAC-GFP recovery was analyzed as time to return to pre-stimulation baseline. tPH-CynA-GFP recovery was analyzed as time to 50% recovery, defined as the first post-peak time point at which the signal had recovered halfway from its peak response toward the pre-stimulation baseline. Recovery-time distributions were analyzed using Kaplan–Meier survival analysis with log-rank comparison. *P < 0.05 where indicated.

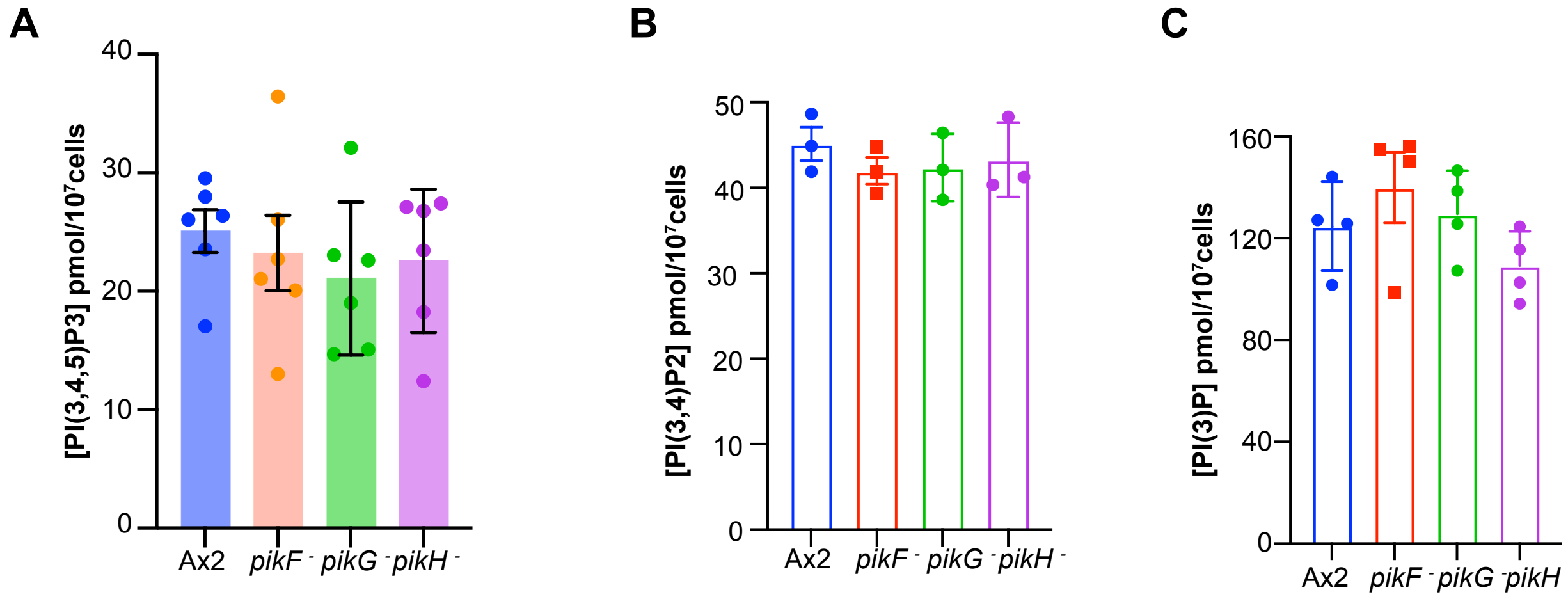


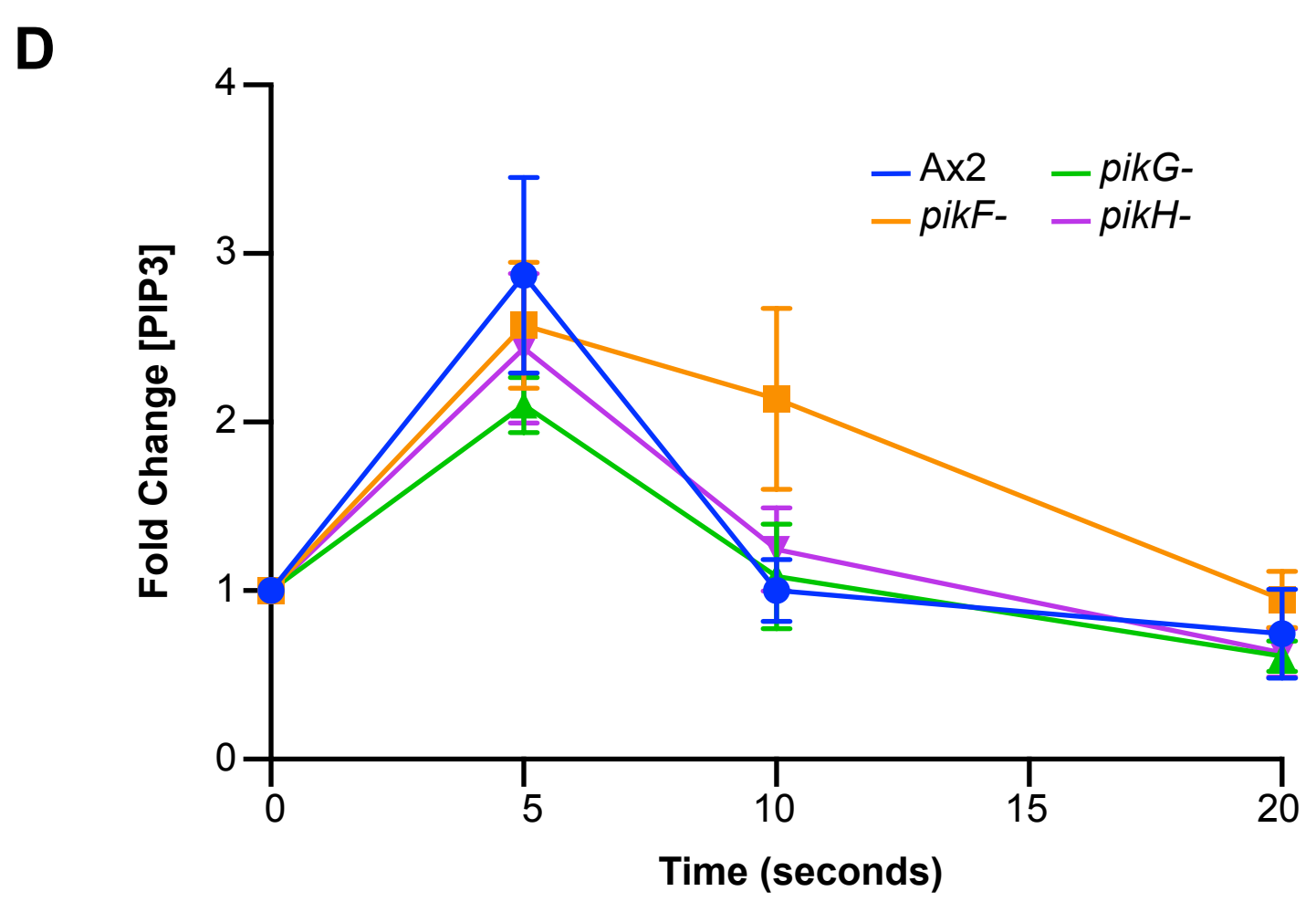


E

| cAMP _PIP3 Stimulations Analysis | | |
|---|---|---|
| Comparison vs Ax2 | Time Course | Response at 10s |
| *pikF-* | *p < 0.03 | *p < 0.05 |
| *pikG-* | n.s. | n.s. |
| *pikH-* | n.s. | n.s. |

**Fig. S5. Basal phosphoinositide levels and cAMP-stimulated PI(3,4,5)$P_3$ dynamics in Ax2 cells and PI3K mutant cells.**

**(A)** Basal PI(3,4,5)$P_3$ levels in developed Ax2 cells, *pikF*⁻ cells, *pikG*⁻ cells, and *pikH*⁻ cells, normalized to cell number and quantified by MASS ELISA. Mean ± s.e.m.; n = 6 independent lipid extractions.

**(B)** Basal PI(3,4)$P_2$ levels in developed Ax2 cells, *pikF*⁻ cells, *pikG*⁻ cells, and *pikH*⁻ cells, normalized to cell number and quantified by MASS ELISA. Mean ± s.e.m.; n = 4 independent lipid extractions.

**(C)** Basal PI3P levels in developed Ax2 cells, *pikF*⁻ cells, *pikG*⁻ cells, and *pikH*⁻ cells, normalized to cell number and quantified by MASS ELISA. Mean ± s.e.m.; n = 3 independent lipid extractions. For A–C, basal lipid levels were not significantly different across strains by Kruskal–Wallis test with Dunn's post hoc correction.

**(D)** Relative change in PI(3,4,5)$P_3$ following cAMP stimulation in developed Ax2 cells, *pikF*⁻ cells, *pikG*⁻ cells, and *pikH*⁻ cells, quantified by MASS ELISA. Fold change was calculated relative to the unstimulated value for each strain within each independent extraction. Mean ± s.e.m.; n = 3 independent lipid extractions. Time-course data were analyzed by mixed-effects model; *pikF*⁻ cells differed significantly from Ax2 cells over time, $P < 0.03$.

**(E)** Statistical summary of the PI(3,4,5)$P_3$ time course shown in D. Data were analyzed using a mixed-effects model with strain, time, and strain × time as fixed effects and extraction as a random effect, followed by Dunnett's multiple-comparisons test comparing each mutant with Ax2 at each time point. PI(3,4,5)$P_3$ was significantly elevated in *pikF*⁻ cells relative to Ax2 cells at 10 s; *pikG*⁻ cells and *pikH*⁻ cells did not differ significantly from Ax2. Adjusted P value thresholds are indicated in the table; n.s., not significant.

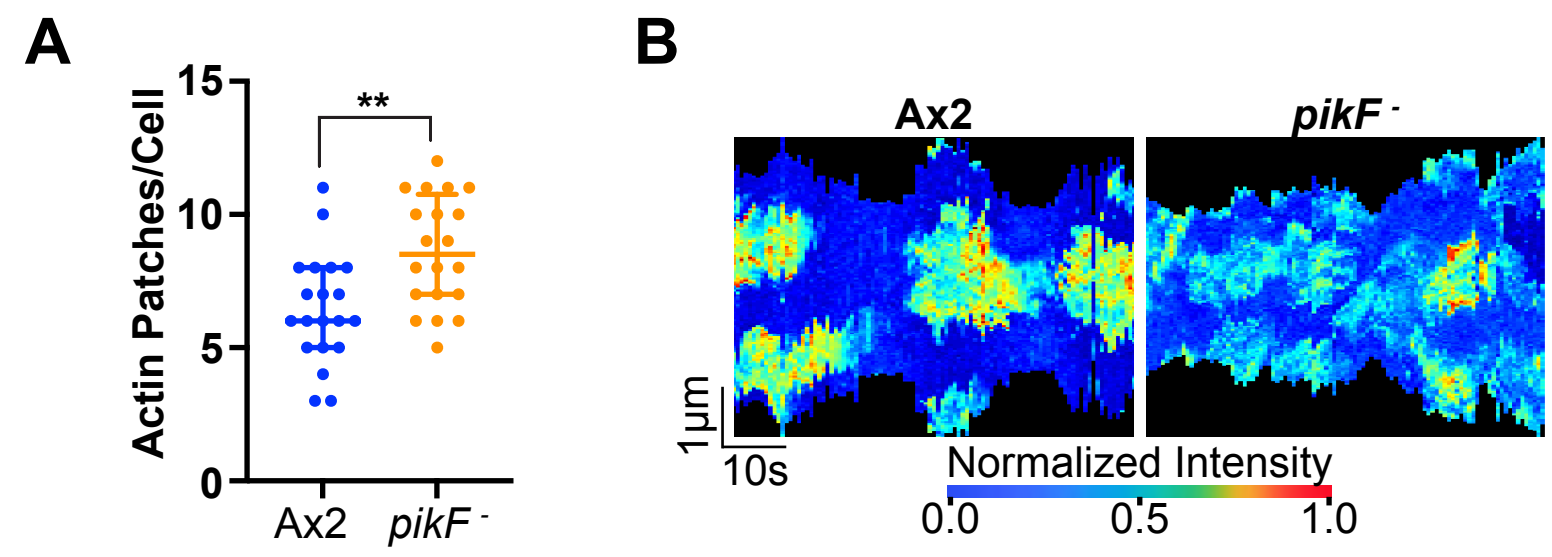


**Fig. S6. *pikF⁻* cells show increased peripheral actin activity.**

**(A)** Quantification of peripheral actin patches in Ax2 cells and *pikF⁻* cells expressing LimE-RFP. Cells were imaged for 500 s, and peripheral actin patches were scored as described in Methods. n = 20 cells per strain across three biological replicates.

**(B)** Representative kymographs of cells quantified in A showing the distribution of peripheral actin patches over time. Statistical significance was assessed by Mann-Whitney test; $**P < 0.001$.

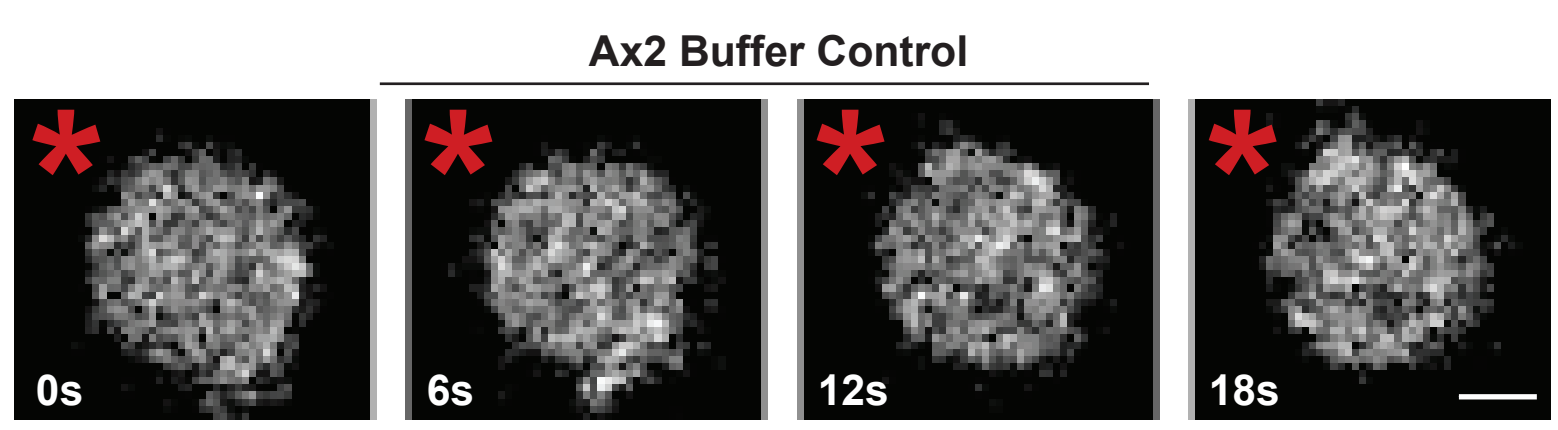


**Fig. S7. Negative control for Ras crescent assay.**

Representative Ras crescent assays in latrunculin-treated Ax2 cells expressing RBD-GFP and exposed to a 0 µM cAMP-filled microcapillary (asterisk). Red asterisk indicates the approximate needle position. Conditions are otherwise as in Fig. 5C,D. Scale bar: 5 µm.

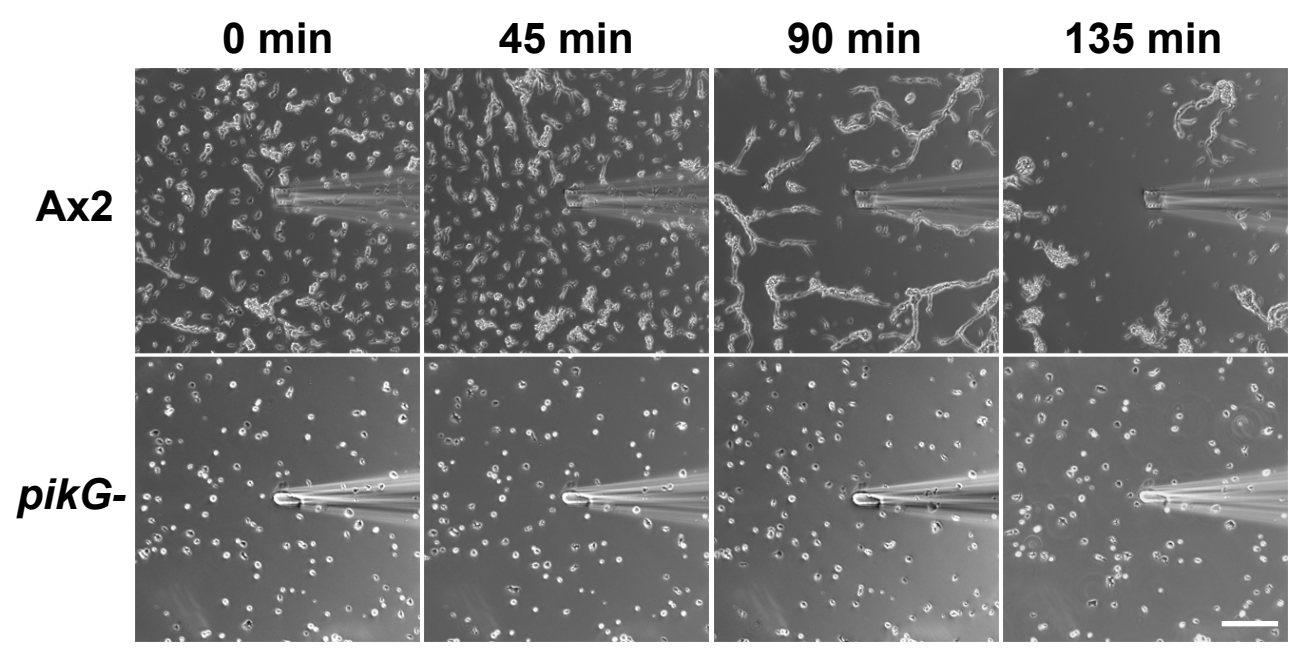


**Fig S8. *pikG*⁻cells fail to stream in the absence of an external cAMP stimulus.**
Time-lapse montages of Ax2 cells and *pikG*⁻cells developed under identical conditions and exposed to a buffer-containing microcapillary as a control for the directed migration assay. Ax2 cells exhibited spontaneous streaming and aggregation, whereas *pikG*⁻cells failed to initiate coordinated streaming, aggregation or mound formation. Representative of n=3 independent experiments. Scale bar: 100 µm.

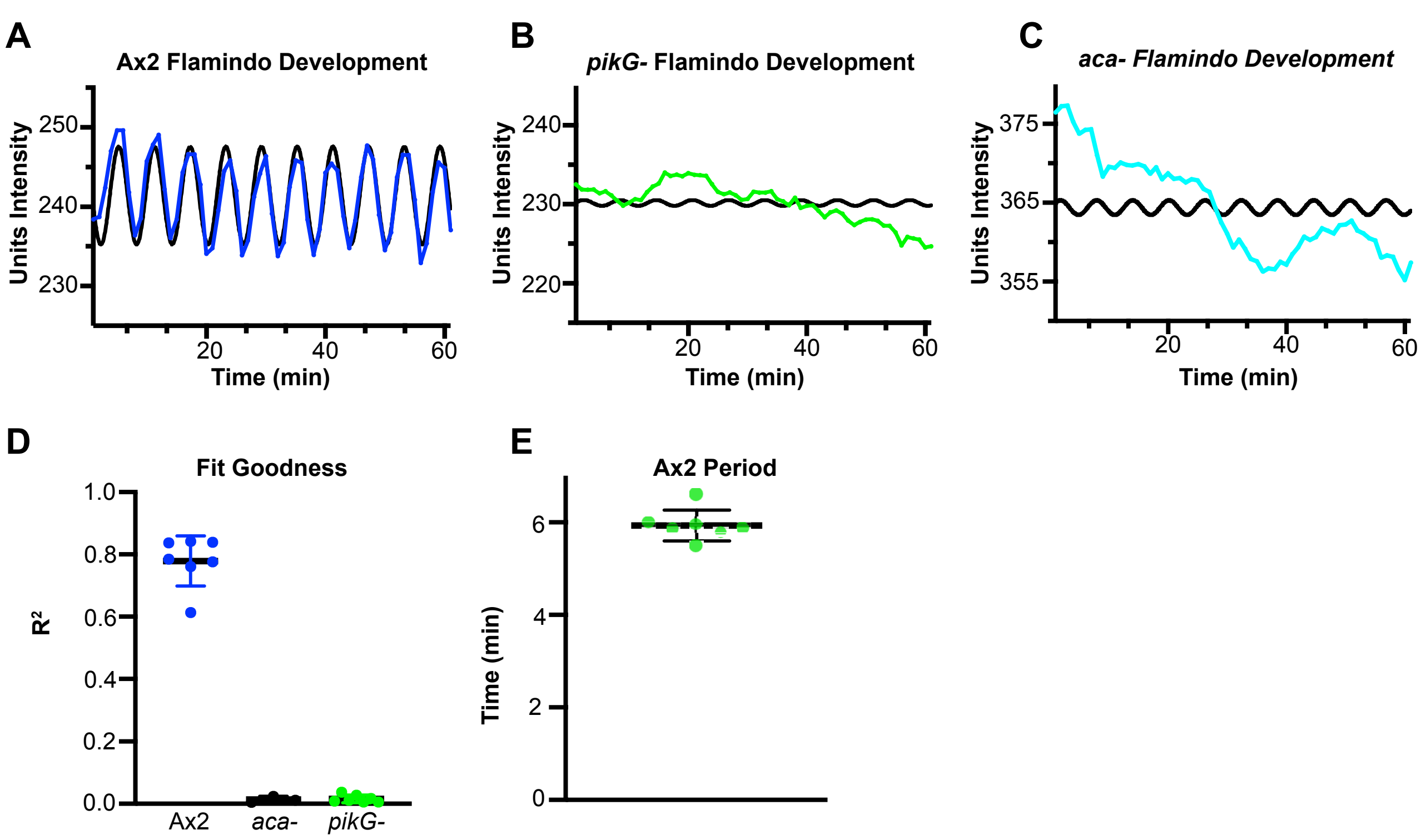


**Fig. S9. Sine curve fitting analysis of Flamindo2 oscillations.**
**(A–C)** Mean intensity traces from representative oscillatory centers in Ax2 cells **(A)**, aca⁻ cells **(B)** and *pikG*⁻cells **(C)**, shown with fitted sine curves (black). Ax2 traces are from the hour before streaming; aca⁻ and *pikG*⁻traces are from 3–4 h after starvation.
**(D)** Goodness-of-fit ($R^2$) values for sine curve fits across Ax2 cells (n=7), aca ⁻ cells (n=3) and *pikG*⁻(n=7) replicates. Error bars indicate median ± s.d.
**(E)** Scatter plot of oscillation periods in Ax2 cells (n=7), calculated from peaks in the mean intensity traces. Error bars indicate median ± s.d.

Journal of Cell Science • Supplementary information

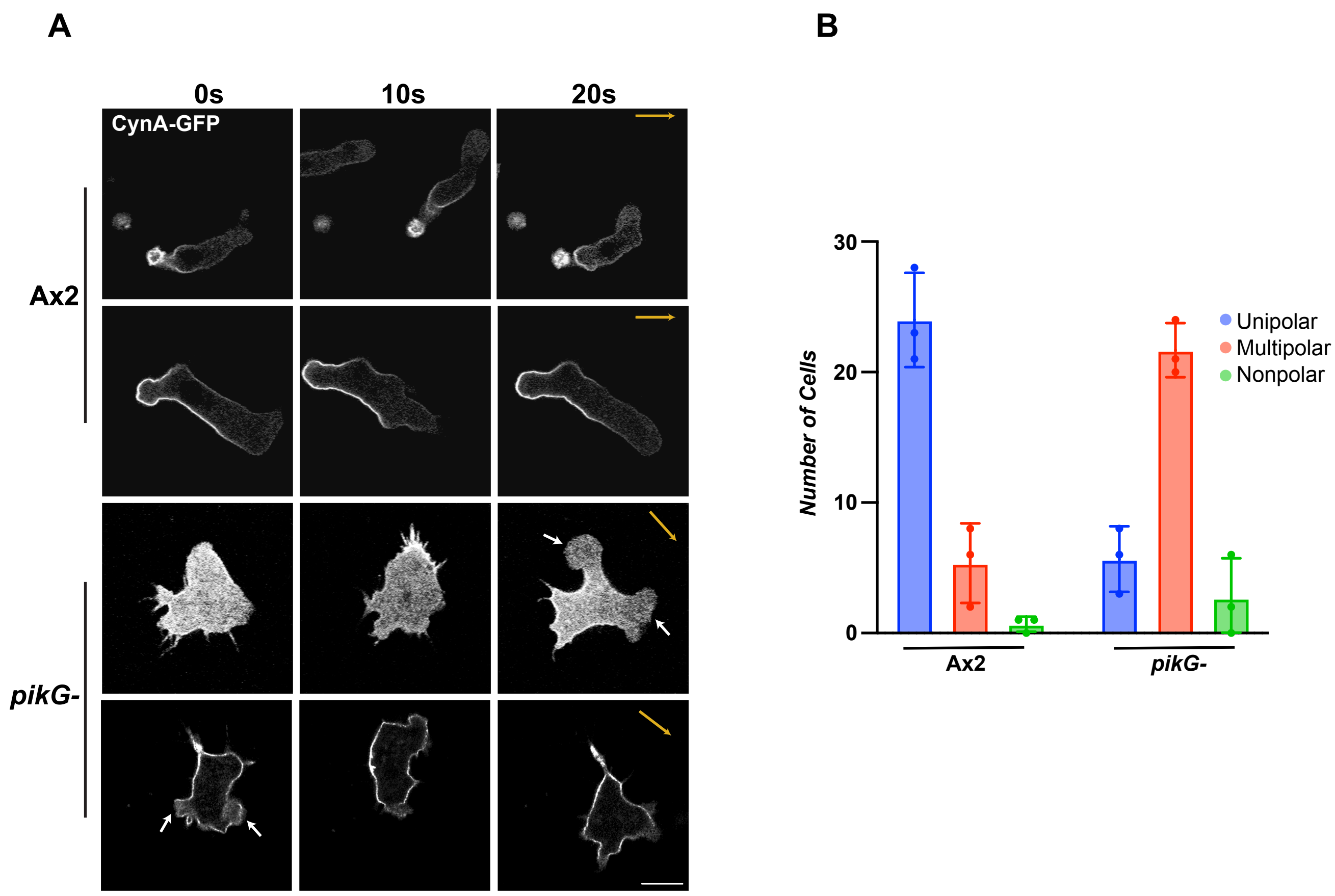


**Fig. S10. *PikG⁻* cells display increased multipolarity during chemotaxis.**
**(A)** Representative time-lapse images of two Ax2 cells and two *pikG⁻* cells migrating toward a cAMP gradient under agar in a two-drop chemotaxis assay. Images were acquired at 10-s intervals, with three consecutive time points shown for each cell. White arrows indicate the fronts of migrating cells, and gold arrows indicate the direction of the cAMP gradient. Scale bar: 2 µm.
**(B)** Cells were classified as unipolar, multipolar or nonpolar as described for Fig. 7, and the number of cells in each category was plotted for each strain. Data show mean ± s.d. from three independent experiments, with 30 cells scored per strain in each experiment. Statistical comparisons were performed on the proportion of cells in each category within each experiment. Ax2 cells and *pikG⁻* cells were compared using paired two-tailed t-tests with Holm–Šídák correction for the two planned comparisons. The proportion of unipolar cells was significantly lower in *pikG⁻* cells than in Ax2 cells (adjusted P=0.0351), whereas the proportion of multipolar cells was significantly higher in *pikG⁻* cells (adjusted P=0.0349). Nonpolar cells were not included in the statistical comparisons.

Journal of Cell Science • Supplementary information

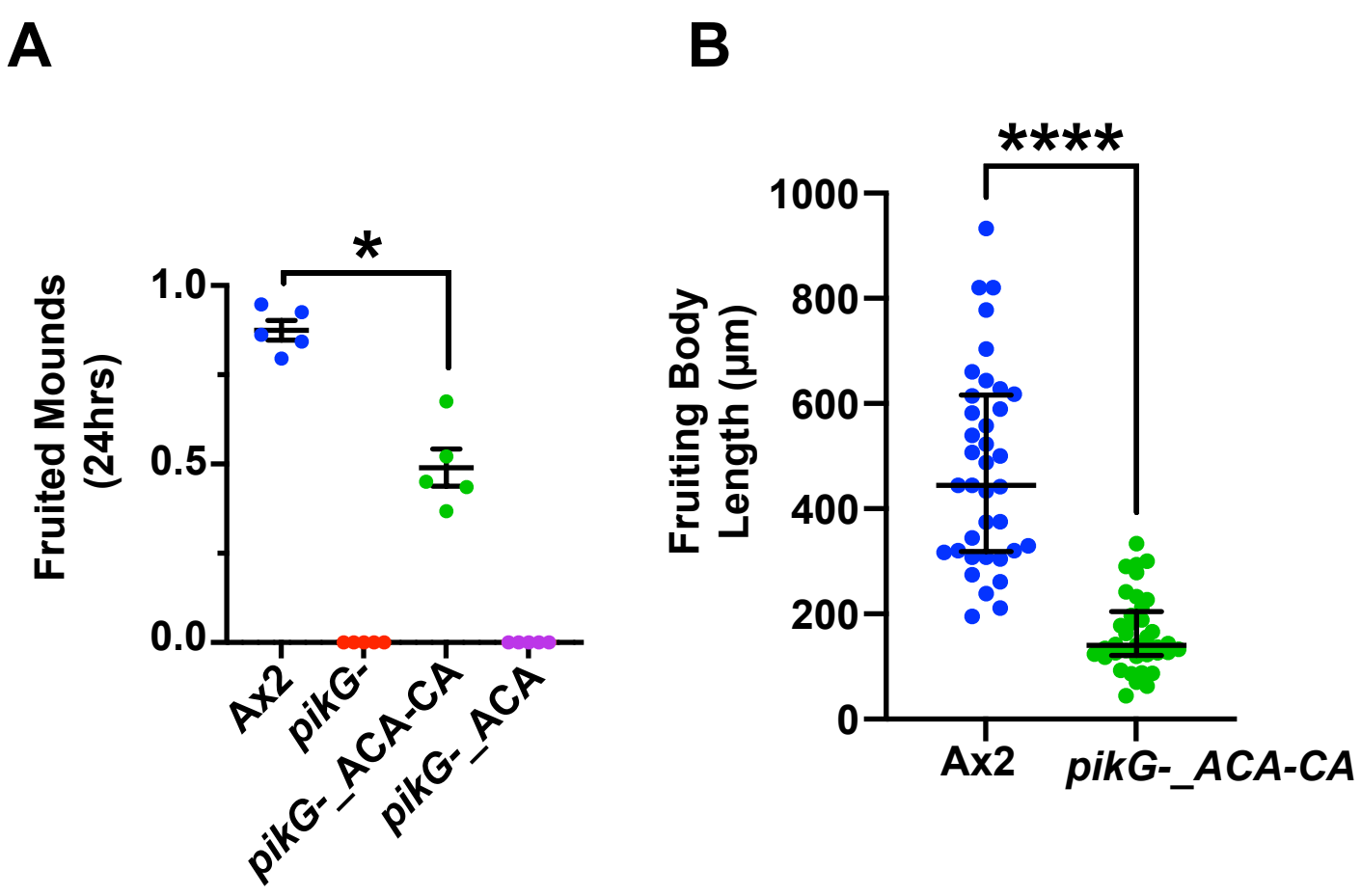


**Fig. S11. Constitutively active ACA partially rescues development in *pikG*⁻cells.**
**(A)** Fraction of mounds reaching the fruiting-body stage by 24 h in Ax2 cells, *pikG*⁻cells, *pikG*⁻ cells expressing ACA-CA-YFP, and *pikG*⁻ cells expressing ACA-YFP. Development and scoring were performed as described for Fig. 8. Approximately 30 mounds were scored per plate across five biological replicates. Data are plotted as mean ± s.e.m. Statistical significance was assessed by mixed-effects analysis. *p <.01
**(B)** Fruiting-body length in Ax2 cells and *pikG*⁻cells expressing ACA-CA-YFP, measured in FIJI/ImageJ. Approximately 100 fruiting bodies were analyzed across three biological replicates. Data are plotted as mean and interquartile range. Statistical significance was assessed using a two-tailed Mann–Whitney test; ****P<0.0001.

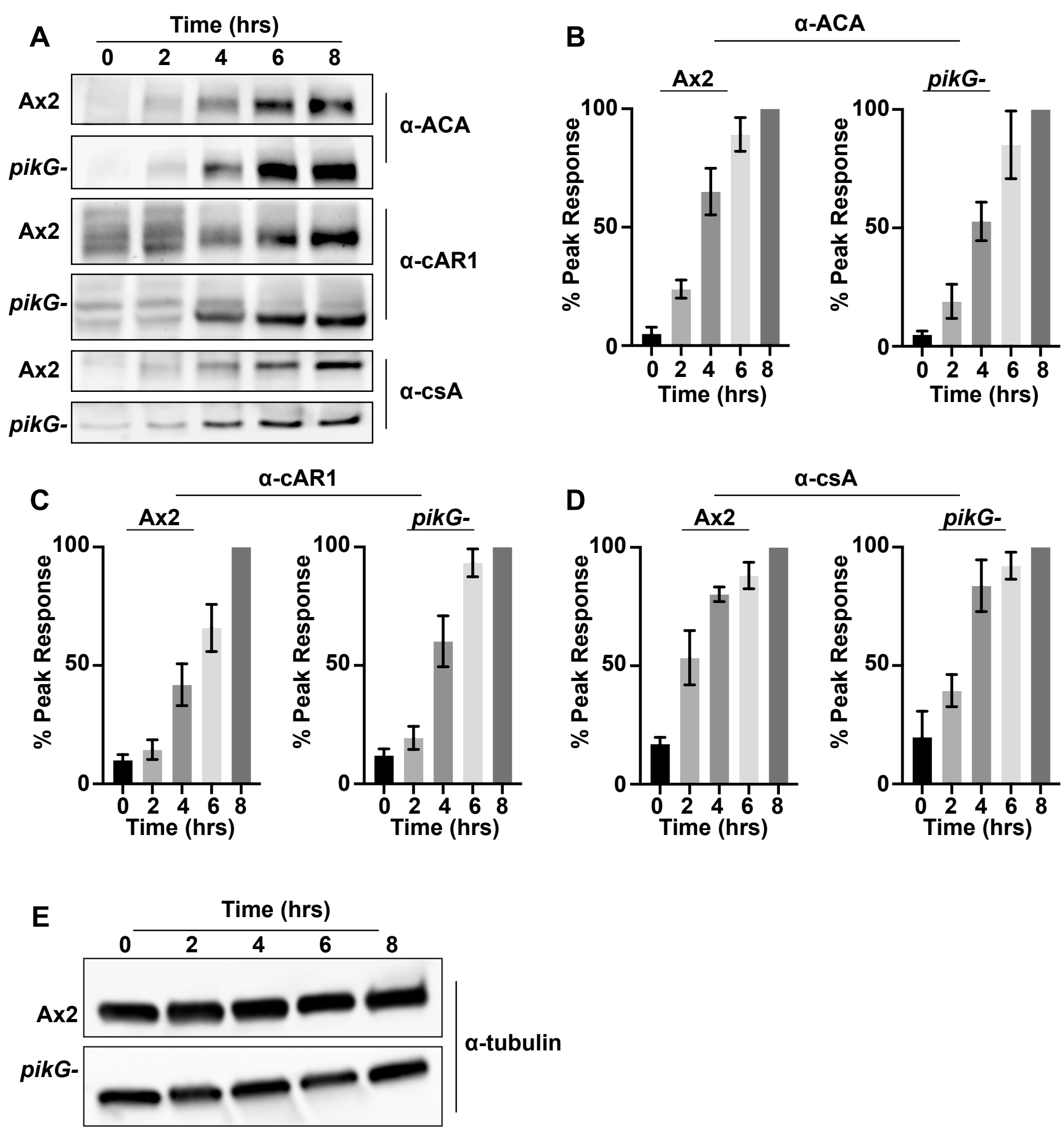


**Fig. S12. Developmental expression of ACA-pathway and aggregation-stage proteins in Ax2 cells and *pikG*$^-$ cells.**

**(A)** Representative immunoblots of ACA, cAR1 and csA in Ax2 cells and *pikG*$^-$ cells collected before development and at the indicated times after the initiation of development. Cells were developed as described in the Methods, and lysates were analyzed using antibodies against ACA, cAR1 and csA.

**(B–D)** Densitometric quantification of ACA (B), cAR1 (C) and csA (D). For each sample, target-band intensity was divided by the corresponding α-tubulin loading-control intensity. Within each biological replicate, the resulting ratios were expressed as a percentage of the Ax2 value at 8 h, which was set to 100% for each protein. Data are plotted as mean ± s.d. from three independent biological replicates.

**(E)** Corresponding α-tubulin loading-control immunoblots for the samples and developmental time points shown in A. Immunoblots are representative of three independent biological replicates.

Journal of Cell Science • Supplementary information

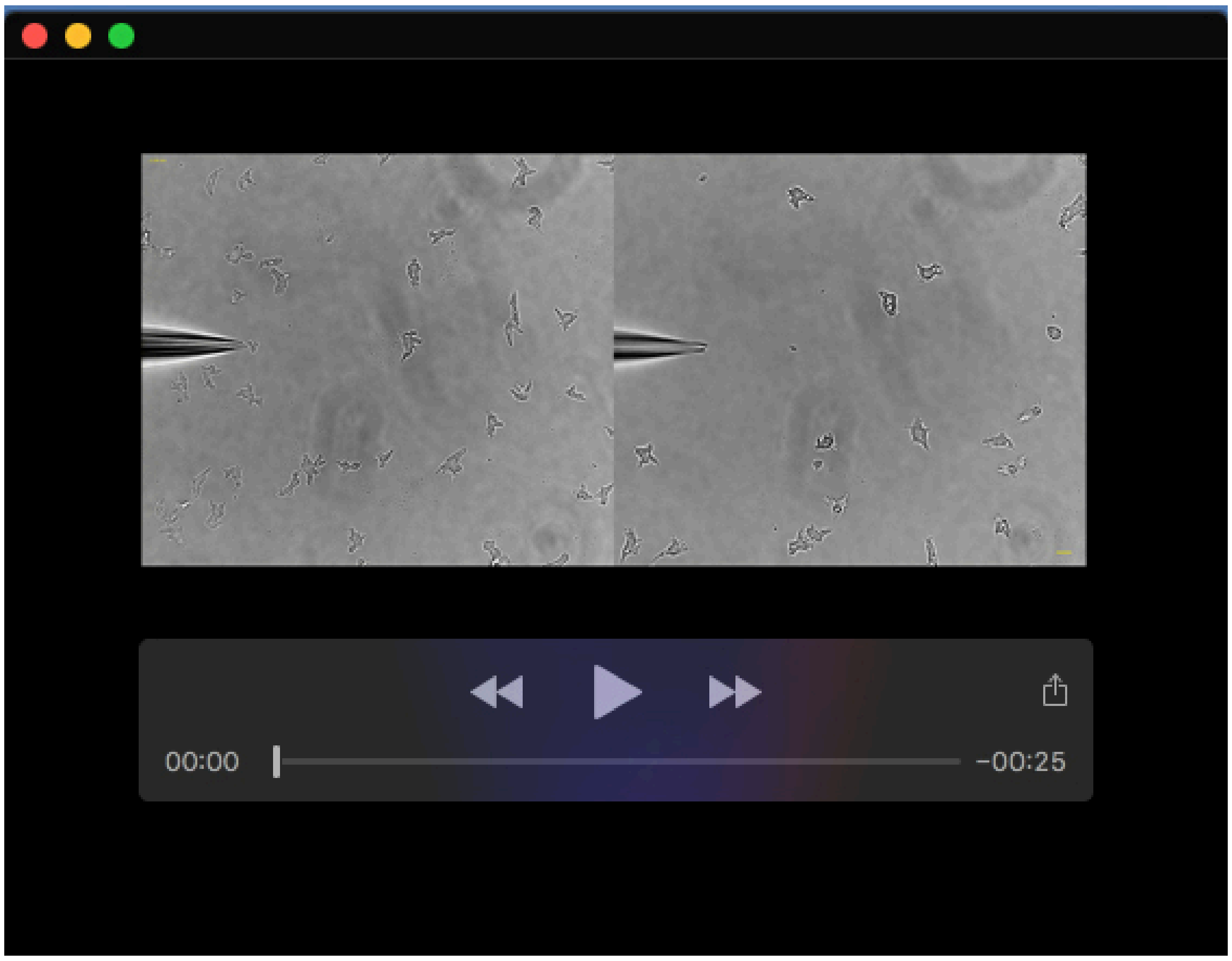


**Movie 1. Micropipette chemotaxis assay of developed Ax2 and *pikF*⁻ cells.**

Time-lapse movie of developed cells migrating toward a micropipette containing 10 µM cAMP. Ax2 cells are shown on the left and *pikF*⁻ cells on the right. Images were acquired on a Nikon Eclipse Ti2-E microscope using a 40×/1.4 NA oil-immersion objective at 6 frames per second and are played back at 25 frames per second. Scale bar, 4 µm.

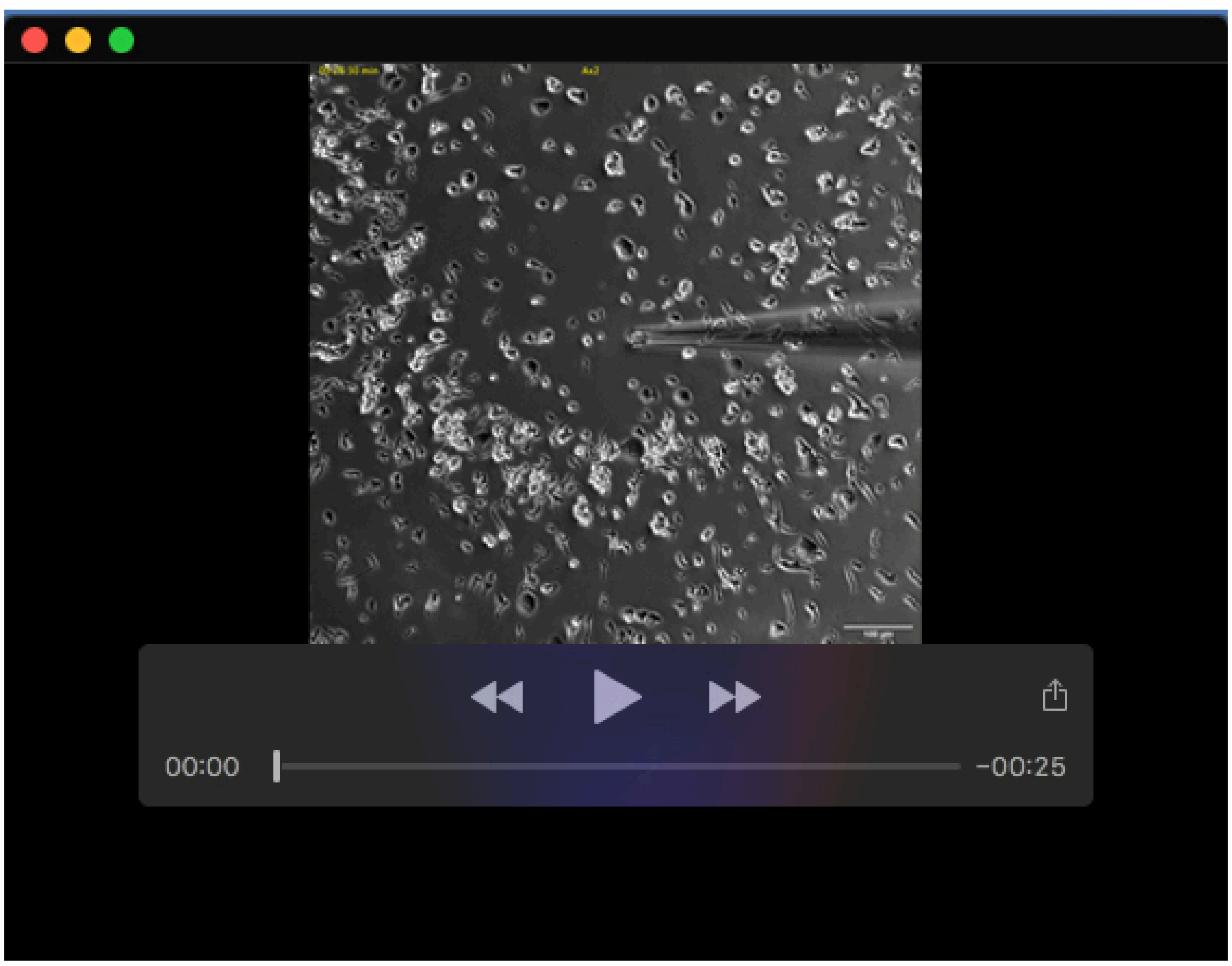


**Movie 2. Micropipette chemotaxis assay of developed Ax2 cells.**

Time-lapse movie of developed Ax2 cells migrating toward a micropipette containing 10 µM cAMP. Images were acquired on a Nikon Eclipse Ti2-E microscope using a 40×/1.4 NA oil-immersion objective at 6 frames per second and are played back at 10 frames per second. Scale bar, 4 µm.

Journal of Cell Science • Supplementary information

J. Cell Sci.: doi:10.1242/jcs.265041: Supplementary information

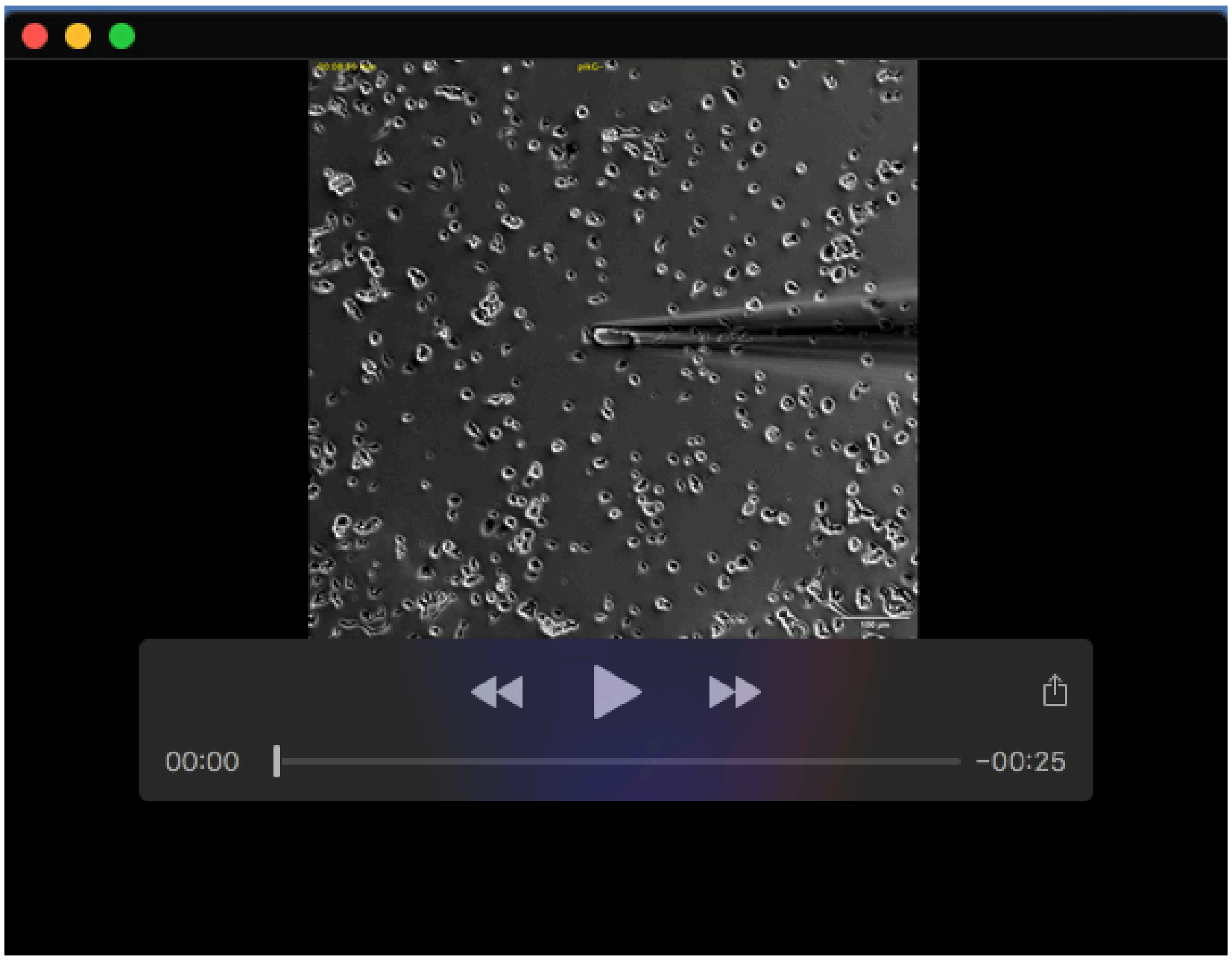


**Movie 3. Micropipette chemotaxis assay of developed *pikG*$^-$ cells.**
Time-lapse movie of developed *pikG*$^-$ cells migrating toward a micropipette containing 10 μM cAMP. Images were acquired on a Nikon Eclipse Ti2-E microscope using a 40×/1.4 NA oil-immersion objective at 6 frames per second and are played back at 10 frames per second. Scale bar, 4 μm.

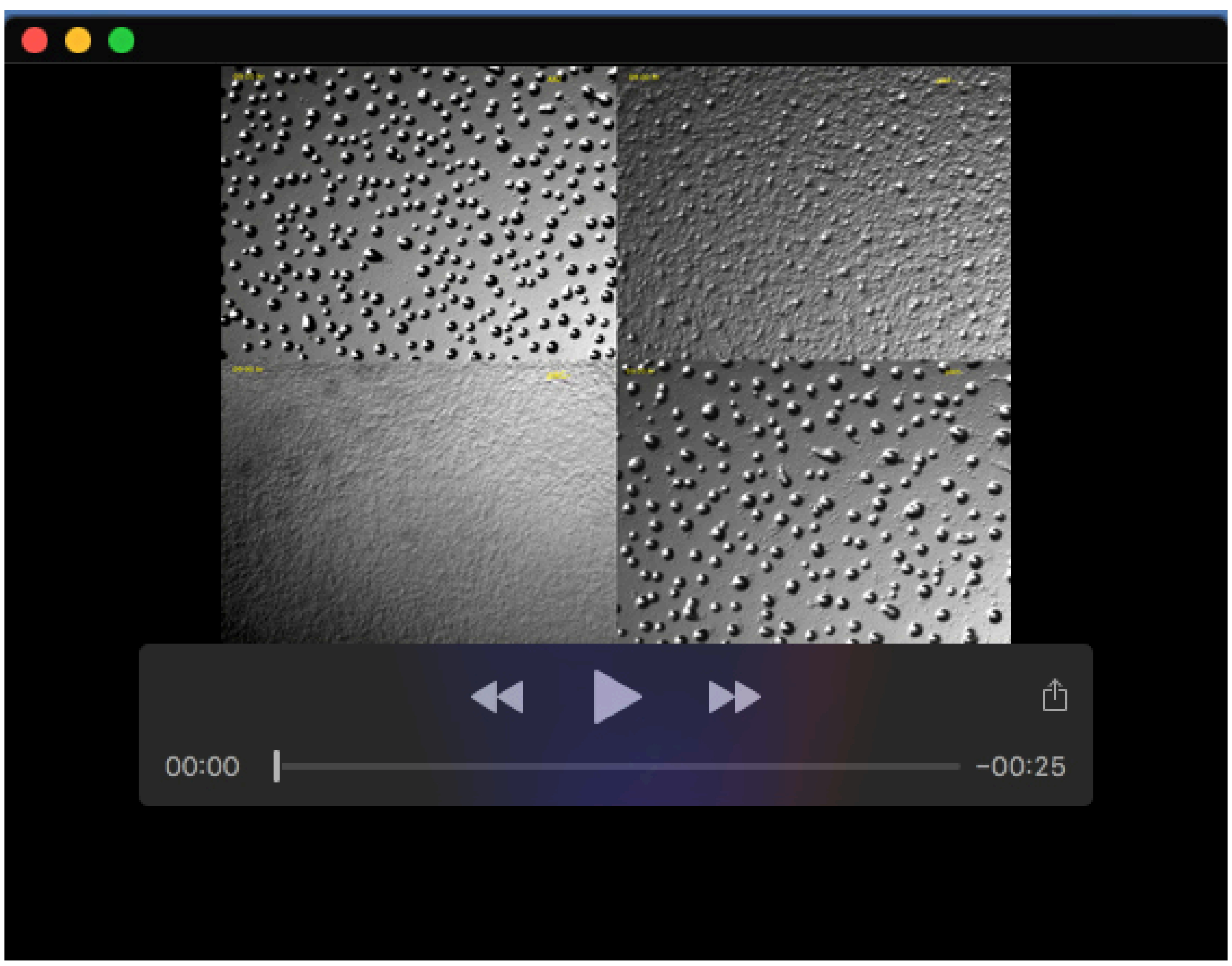


**Movie 4. Development on non-nutrient agar.**

Time-lapse movie showing development of the indicated *Dictyostelium* strains on 1% non-nutrient KK2 agar. Strains are labeled in the movie. Images were acquired every 10 min for 48 h at 7.3x magnification and are played back at 15 frames per second. Scale bar, 500 μm.

Journal of Cell Science • Supplementary information

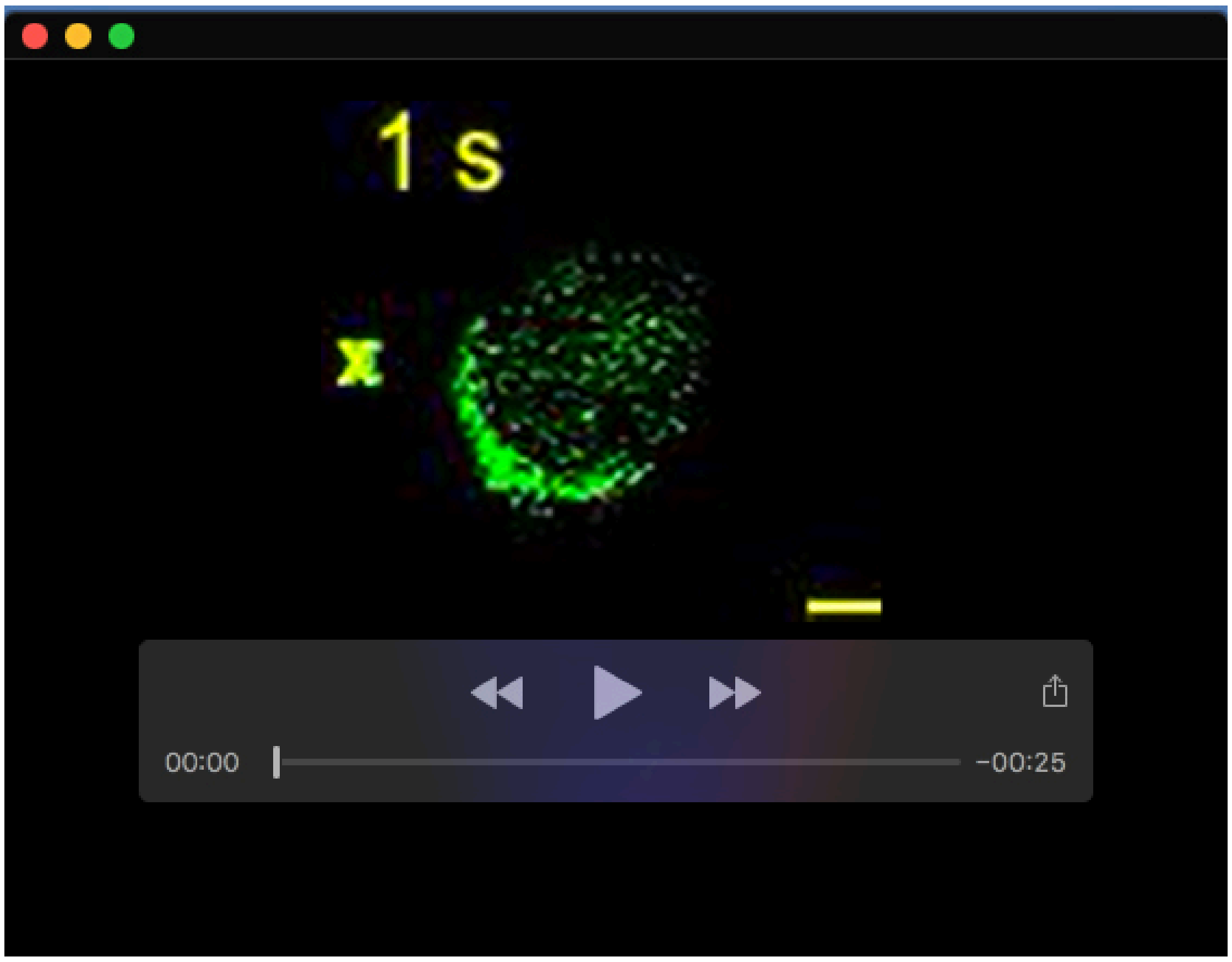


**Movie 5. Ras crescent assay in developed Ax2 cells expressing Raf1-RBD-GFP.**
Time-lapse movie of developed Ax2 cells expressing Raf1-RBD-GFP in response to a micropipette containing 10 μM cAMP. The position of the needle is indicated by an asterisk. Images were acquired every 6 s on a Nikon Eclipse Ti2-E microscope equipped with a C2 confocal module using a 63×/1.4 NA objective and are played back at 10 frames per second. Scale bar, 2 μm.

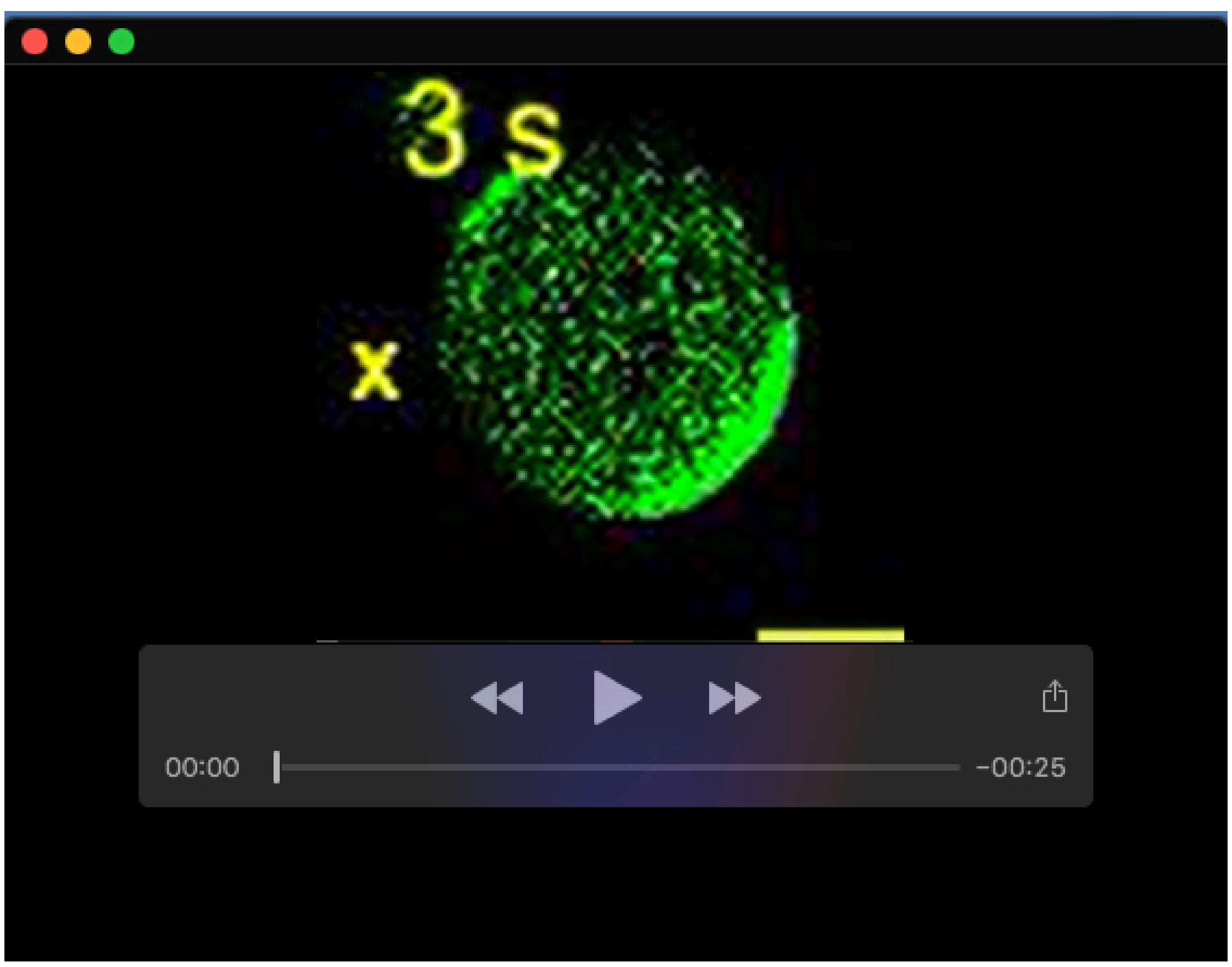


**Movie 6. Ras crescent assay in developed *pikF⁻* cells expressing Raf1-RBD-GFP.**
Time-lapse movie of developed *pikF⁻* cells expressing Raf1-RBD-GFP in response to a micropipette containing 10 μM cAMP. The position of the needle is indicated by an asterisk. Images were acquired every 6 s on a Nikon Eclipse Ti2-E microscope equipped with a C2 confocal module using a 63×/1.4 NA objective and are played back at 10 frames per second. Scale bar, 2 μm.

Journal of Cell Science • Supplementary information

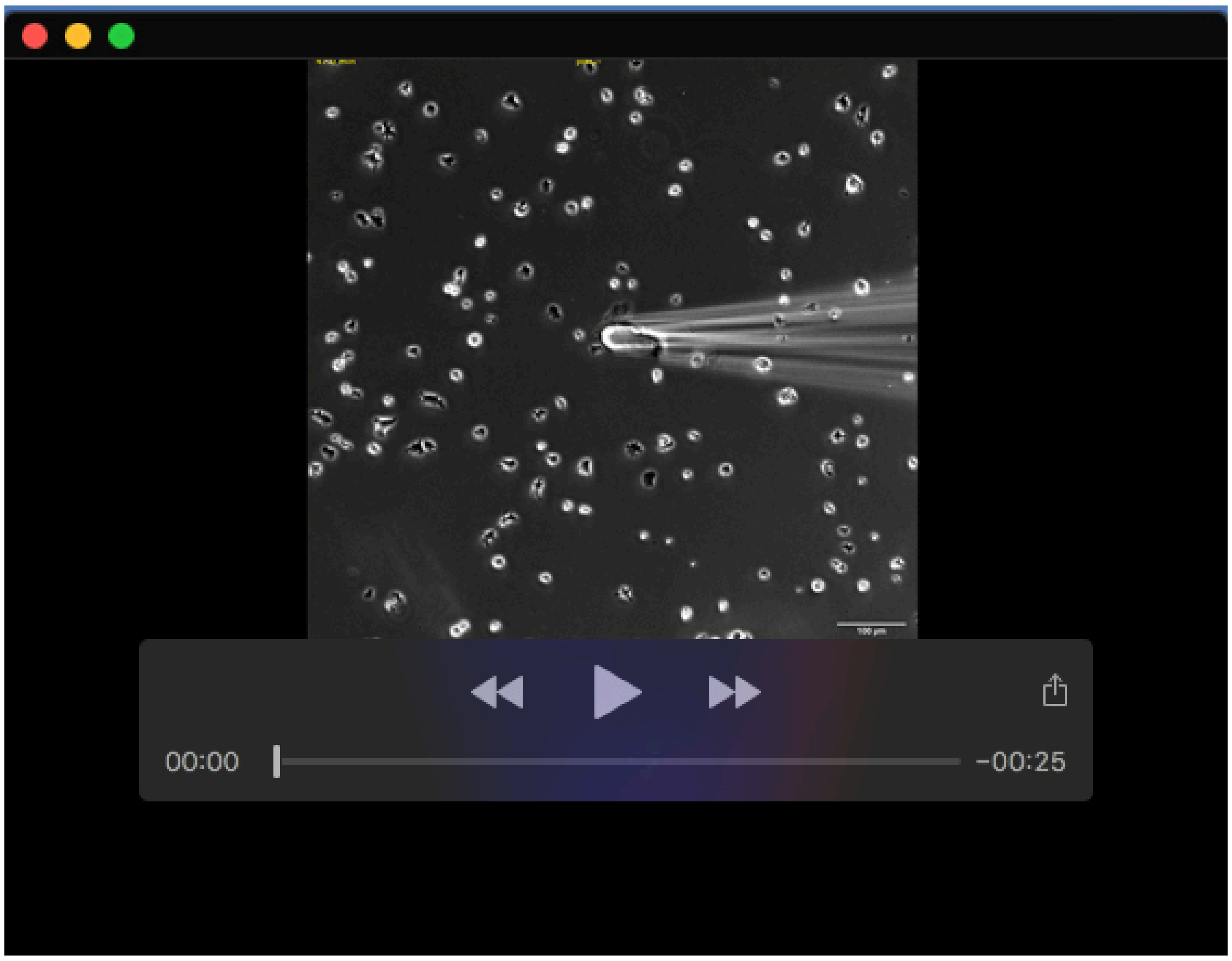


**Movie 7. Buffer-control micropipette assay of developed Ax2 cells.**
Time-lapse movie of developed Ax2 cells exposed to a micropipette containing 0 μM cAMP. Images were acquired on a Nikon Eclipse Ti2-E microscope using a 10×/0.55 NA objective at 6 frames per second and are played back at 10 frames per second.

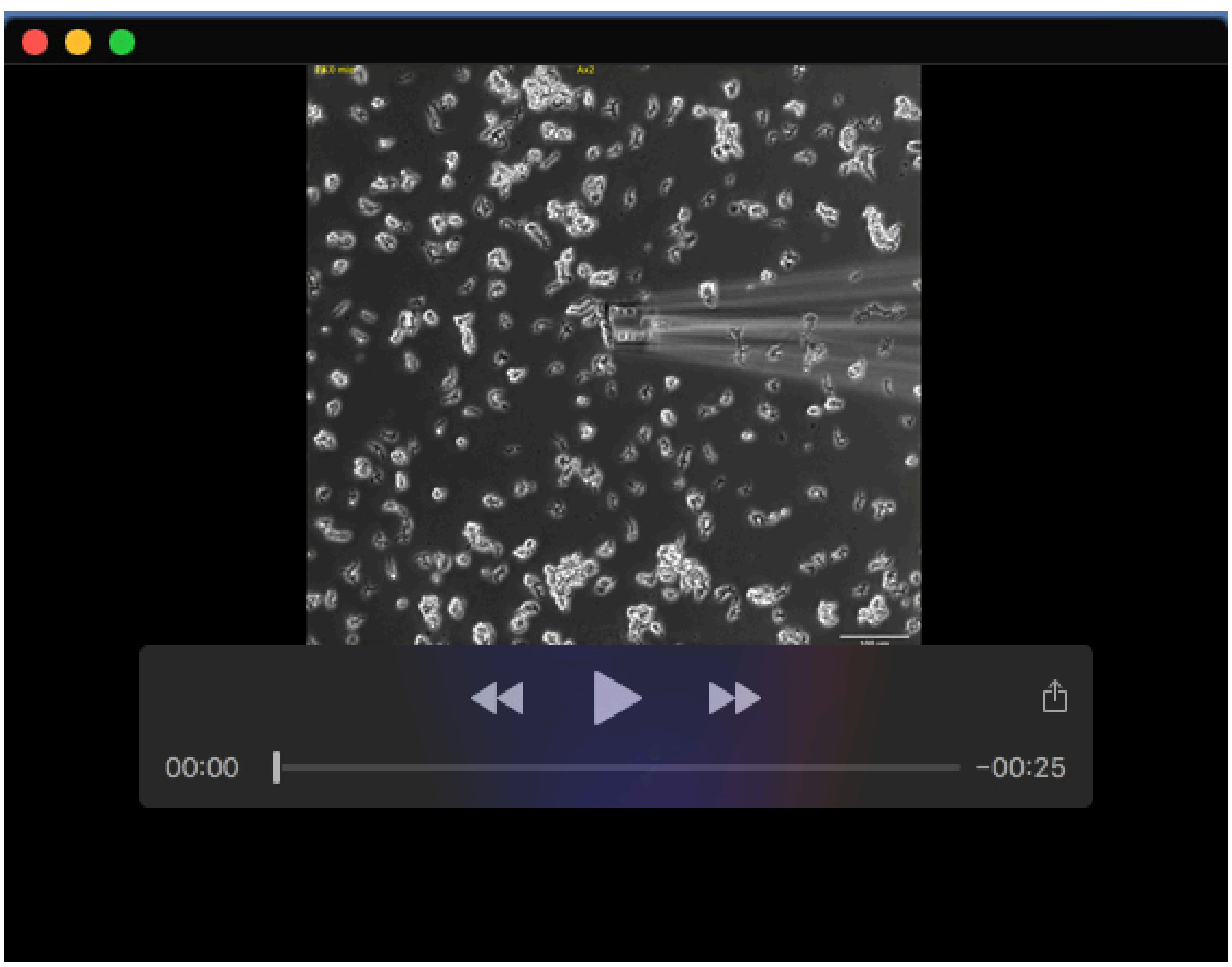


**Movie 8. Buffer-control micropipette assay of developed *pikG*⁻ cells.**
Time-lapse movie of developed *pikG*⁻ cells exposed to a micropipette containing 0 μM cAMP. Images were acquired on a Nikon Eclipse Ti2-E microscope using a 10×/0.55 NA objective at 6 frames per second and are played back at 10 frames per second.

Journal of Cell Science • Supplementary information

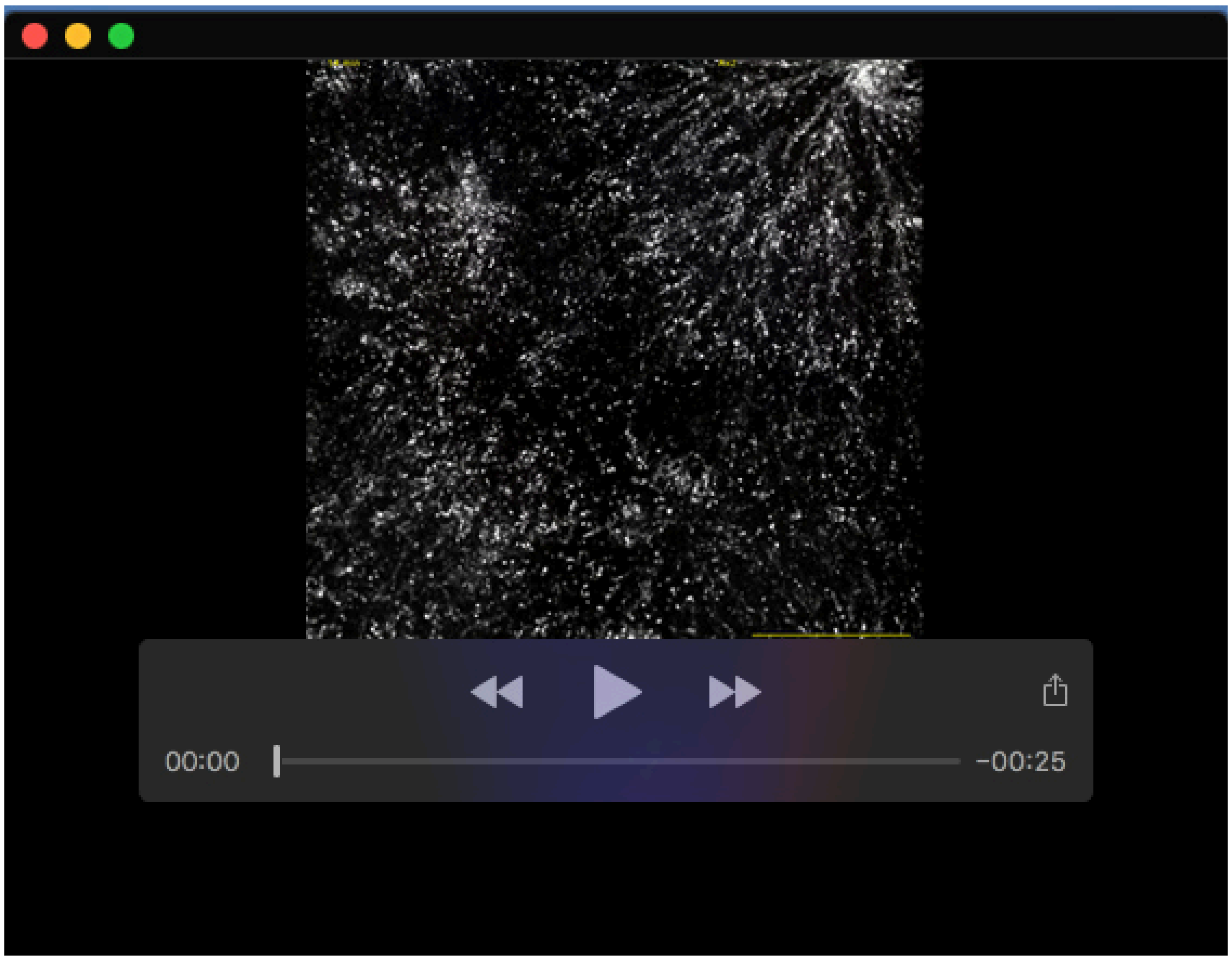


**Movie 9. Time-lapse imaging of developed Ax2 cells expressing Flamindo2-mScarlet.**
Time-lapse movie of developed Ax2 cells expressing Flamindo2-mScarlet. Images were acquired every 60 s for 8 h on a Nikon Eclipse Ti2-E microscope at 5× magnification. Scale bar, 500 μm.

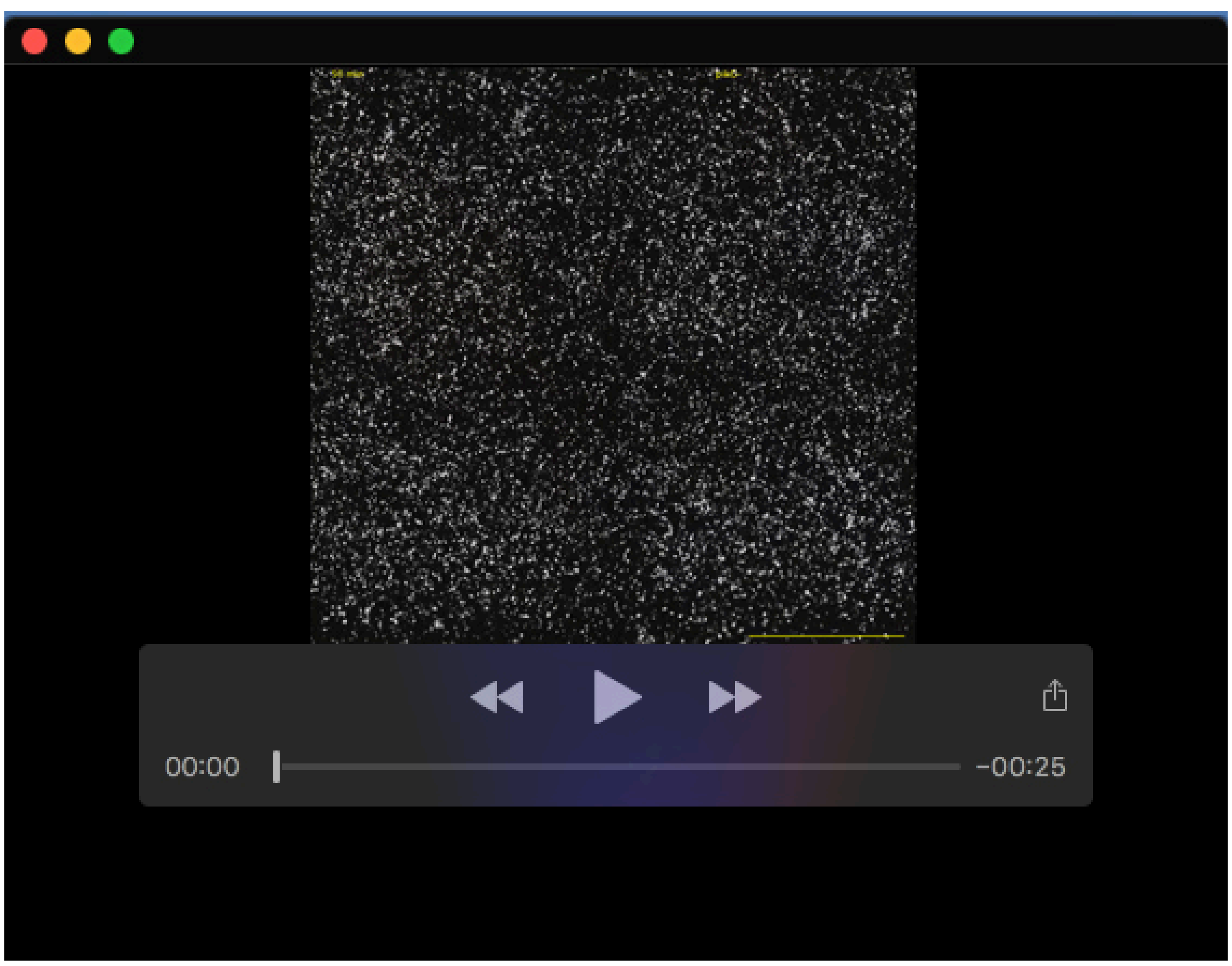


**Movie 10. Time-lapse imaging of developed *pikG*$^{-}$ cells expressing Flamindo2-mScarlet.**
Time-lapse movie of developed *pikG*$^{-}$ cells expressing Flamindo2-mScarlet. Images were acquired every 60 s for 8 h on a Nikon Eclipse Ti2-E microscope at 5× magnification. Scale bar, 500 μm.

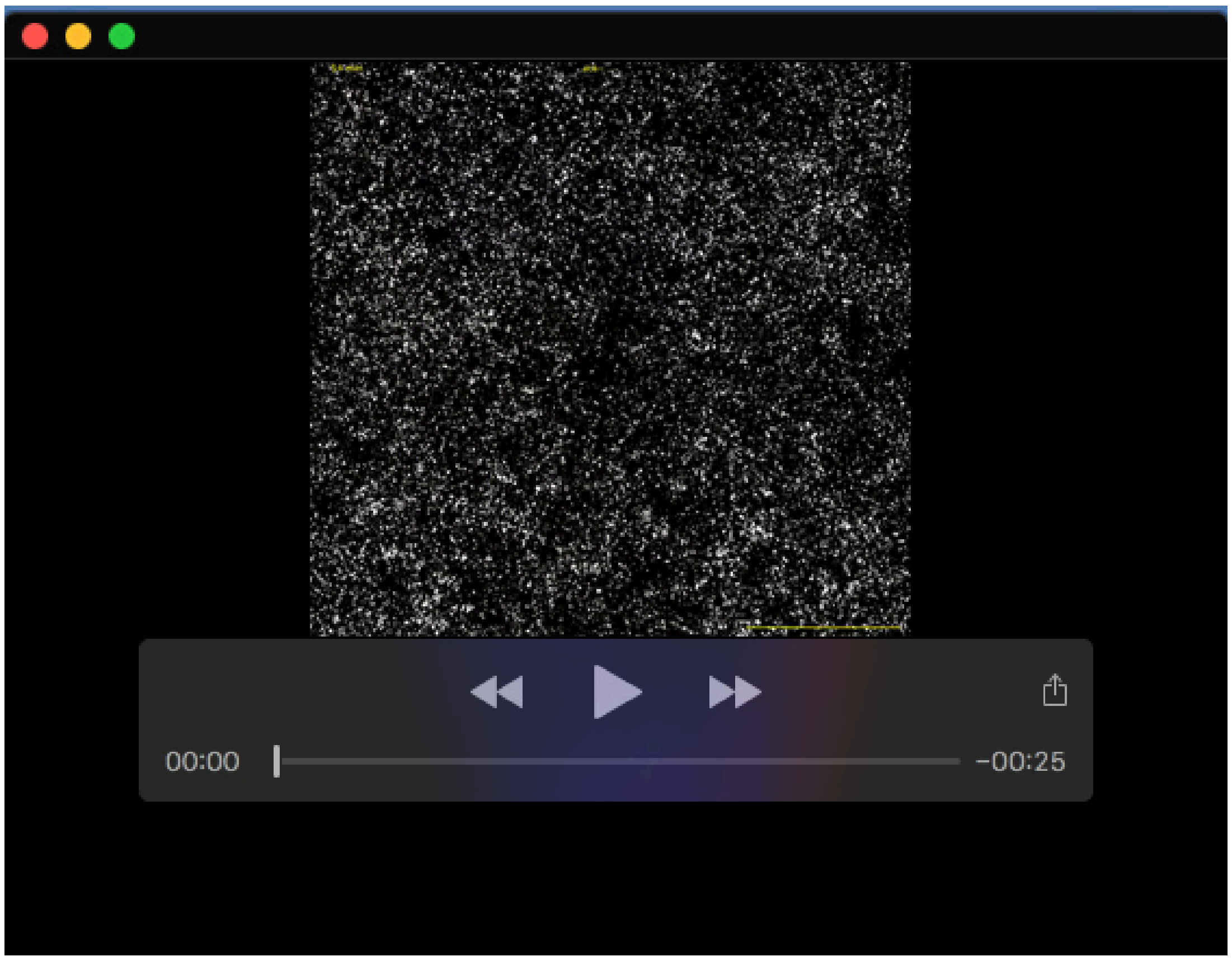


**Movie 11. Time-lapse imaging of developed aca⁻ cells expressing Flamindo2-mScarlet.**
ime-lapse movie of developed aca⁻ cells expressing Flamindo2-mScarlet. Images were acquired every 60 s for 8 h on a Nikon Eclipse Ti2-E microscope at 5× magnification. Scale bar, 500 µm.

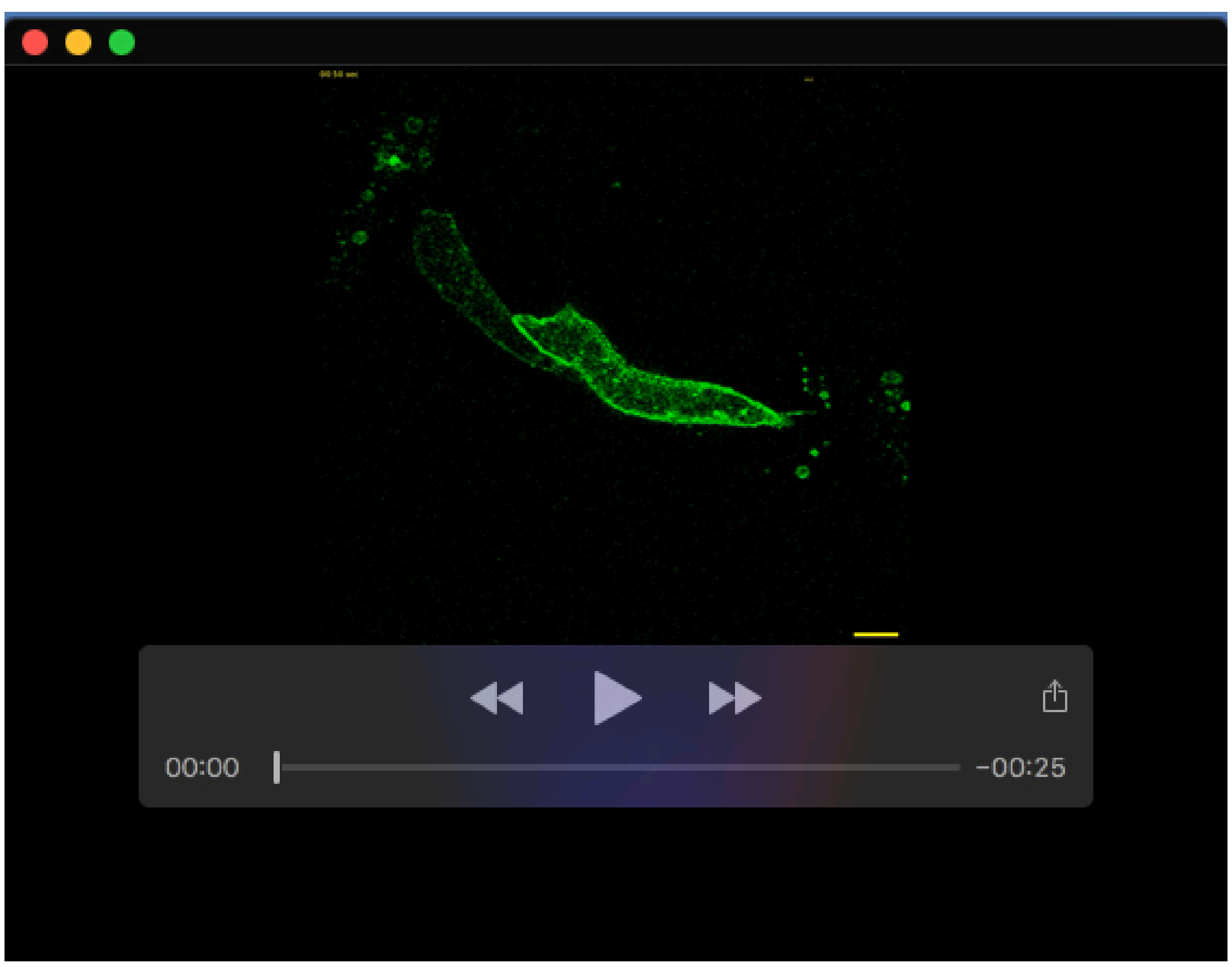


**Movie 12. Streaming behavior of developed Ax2 cells expressing ACA-YFP.**
Time-lapse movie of developed Ax2 cells expressing ACA-YFP. Images were acquired every 10 s on a Zeiss LSM 980 with Airyscan using a 63×/1.40 NA oil-immersion objective and are played back at 5 frames per second. Scale bar, 5 µm.

Journal of Cell Science • Supplementary information

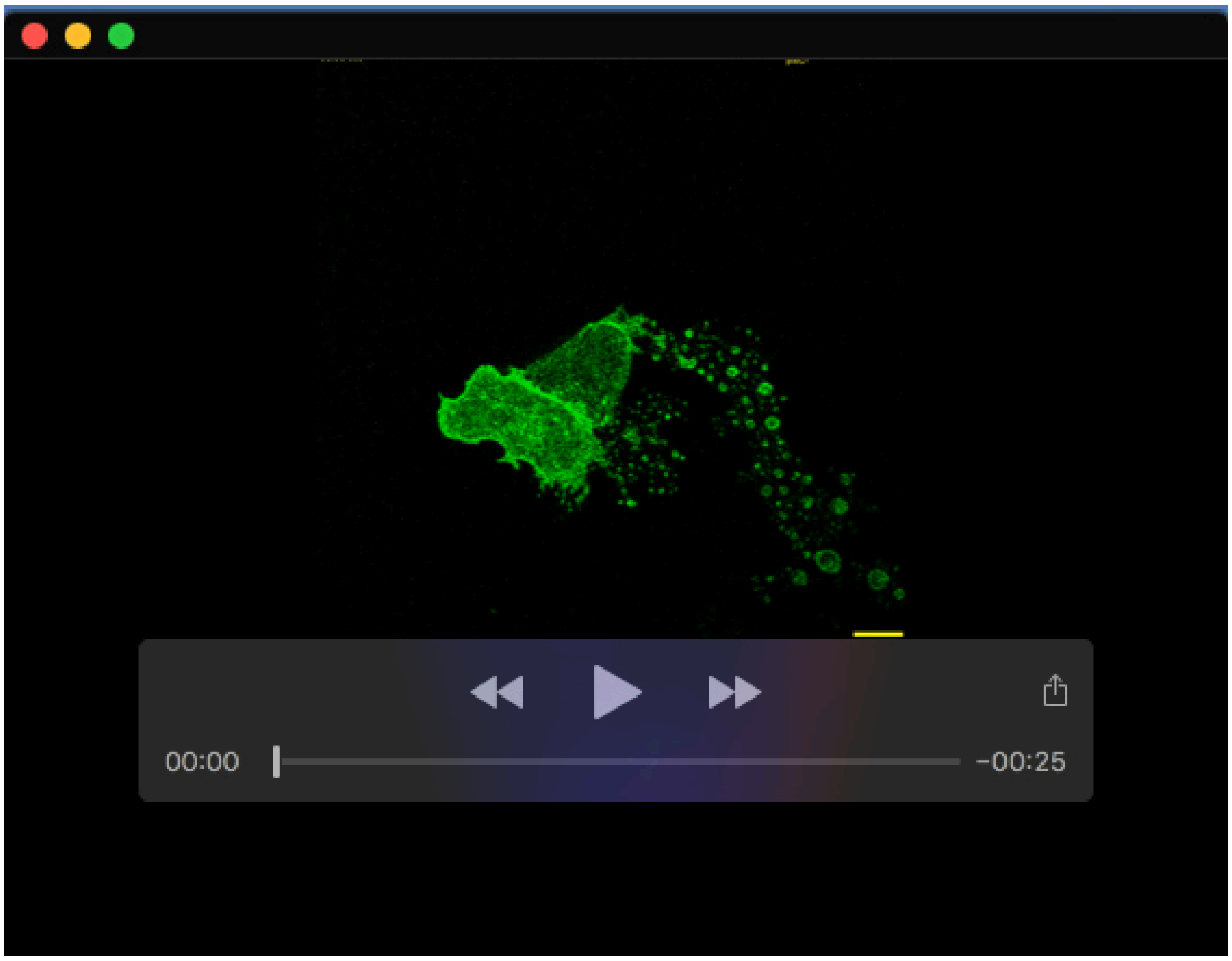


**Movie 13. Streaming behavior of developed *pikG*⁻ cells expressing ACA-YFP.**
Time-lapse movie of developed *pikG*⁻ cells expressing ACA-YFP. Images were acquired every 10 s on a Zeiss LSM 980 with Airyscan using a 63×/1.40 NA oil-immersion objective and are played back at 5 frames per second. Scale bar, 5 µm.

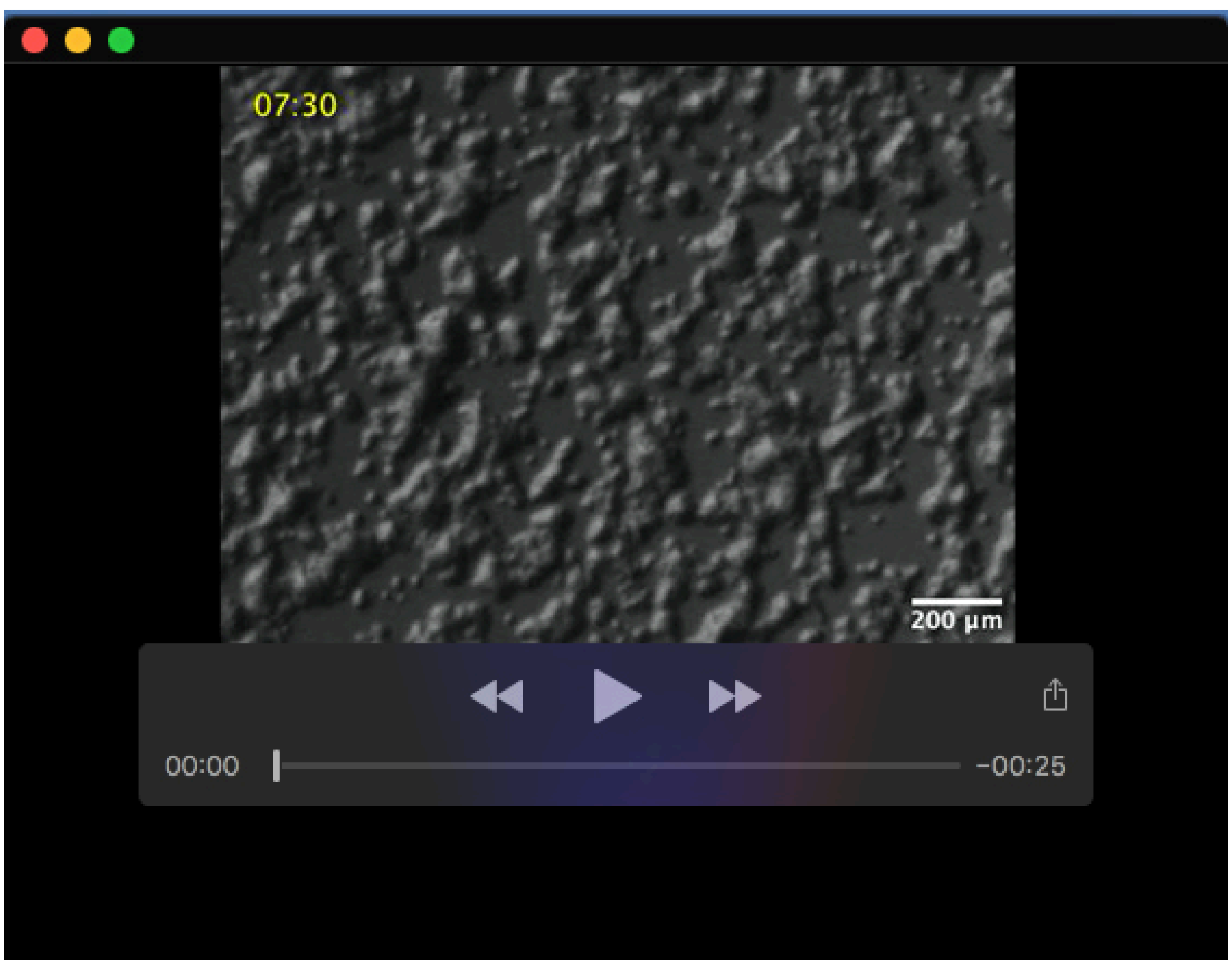


**Movie 14. ACA-CA rescue of pikG⁻ development.**

Time-lapse movie showing development of Ax2, pikG⁻, pikG⁻ cells expressing ACA-YFP, and pikG⁻ cells expressing ACA-CA-YFP on 1% non-nutrient KK2 agar. Strains are labeled in the movie. Images were acquired every 10 min for 48 h at 7.3× magnification and are played back at 15 frames per second. Scale bar, 500 µm.

Journal of Cell Science • Supplementary information
